# Supplementary material for: Zika virus remodels and hijacks IGF2BP2 ribonucleoprotein complex to promote viral replication organelle biogenesis
Source: eLife. 2024 Nov 20;13:RP94347. doi: 10.7554/eLife.94347 (PMC11578589; doi:10.7554/eLife.94347)

Figure 2A

Anti-IGF2BP2

1- Mock shNT
2- Mock shIGF2BP2
3- ZIKV shNT
4- ZIKV shIGF2BP2

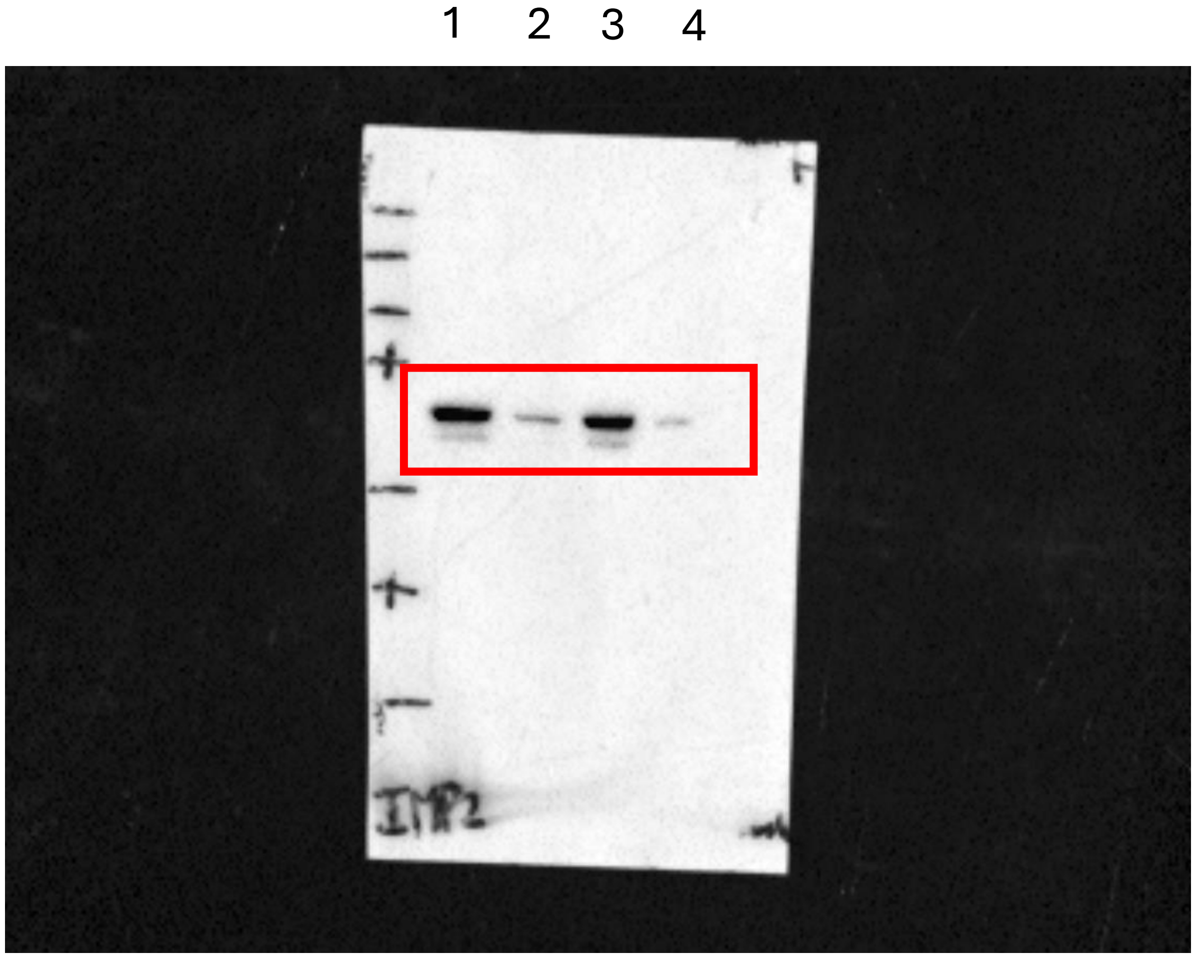

Anti-NS3

1- Mock shNT
2- Mock shIGF2BP2
3- ZIKV shNT
4- ZIKV shIGF2BP2

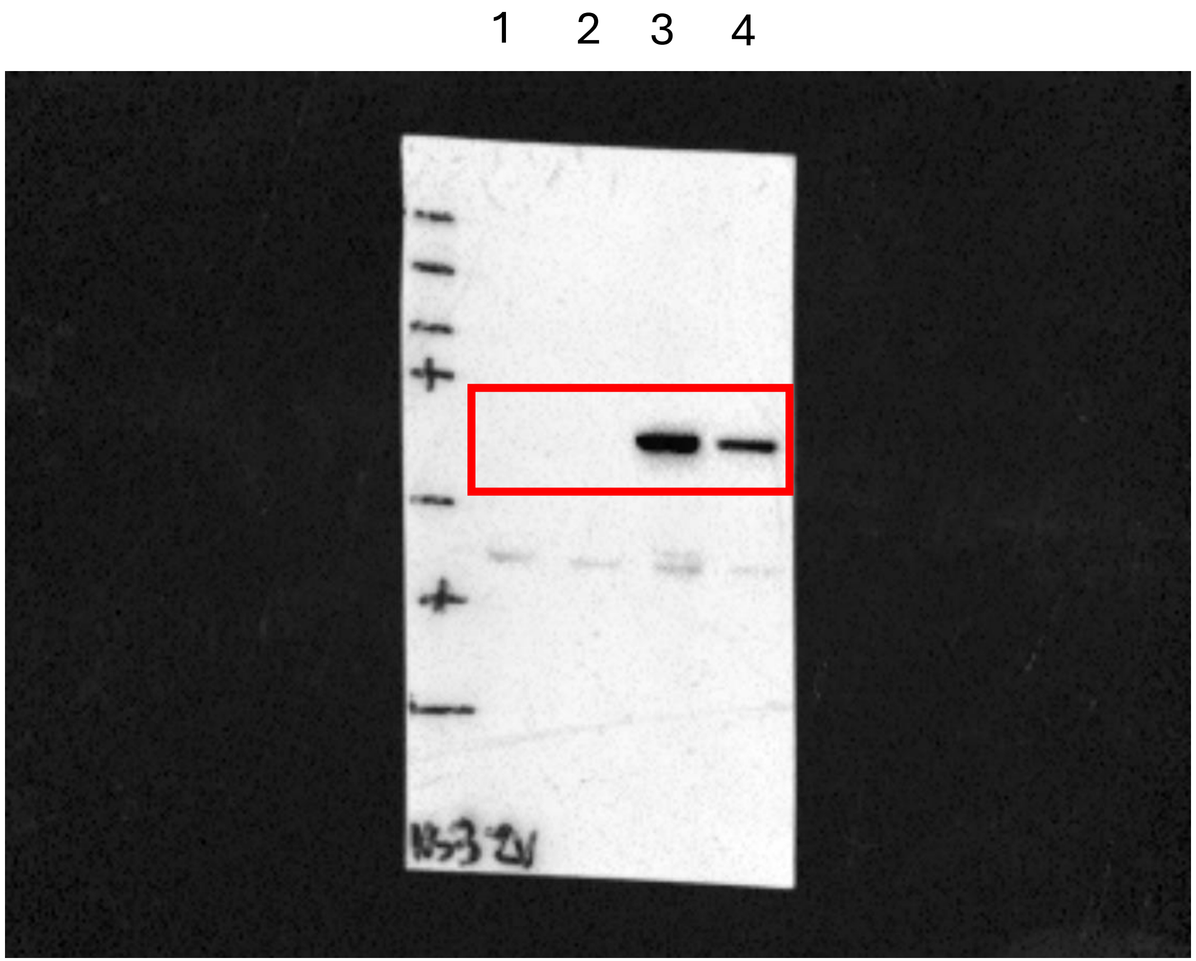

Anti-NS4A ZIKV

1- Mock shNT
2- Mock shIGF2BP2
3- ZIKV shNT
4- ZIKV shIGF2BP2

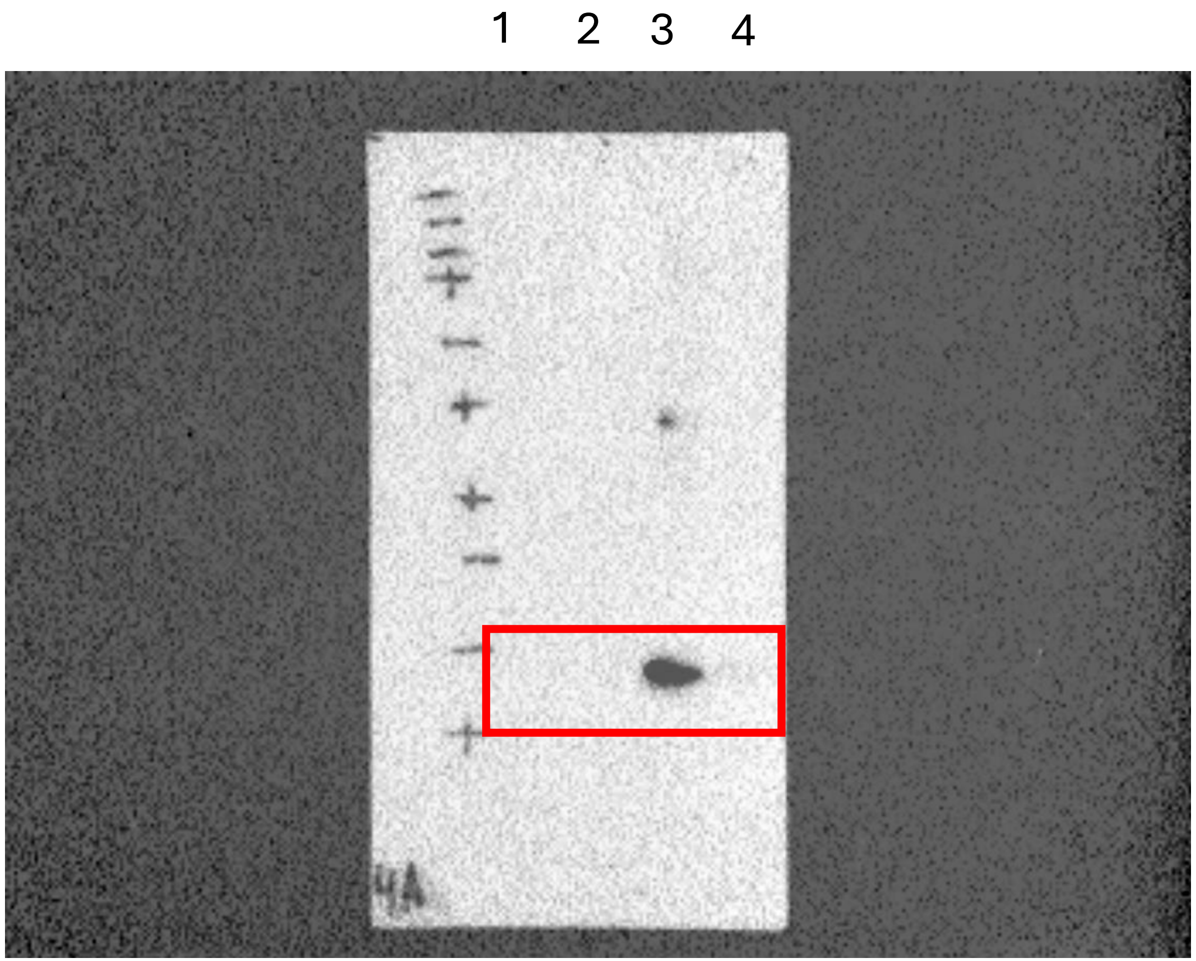

Anti-NS5 ZIKV

1- Mock shNT
2- Mock shIGF2BP2
3- ZIKV shNT
4- ZIKV shIGF2BP2

1 2 3 4

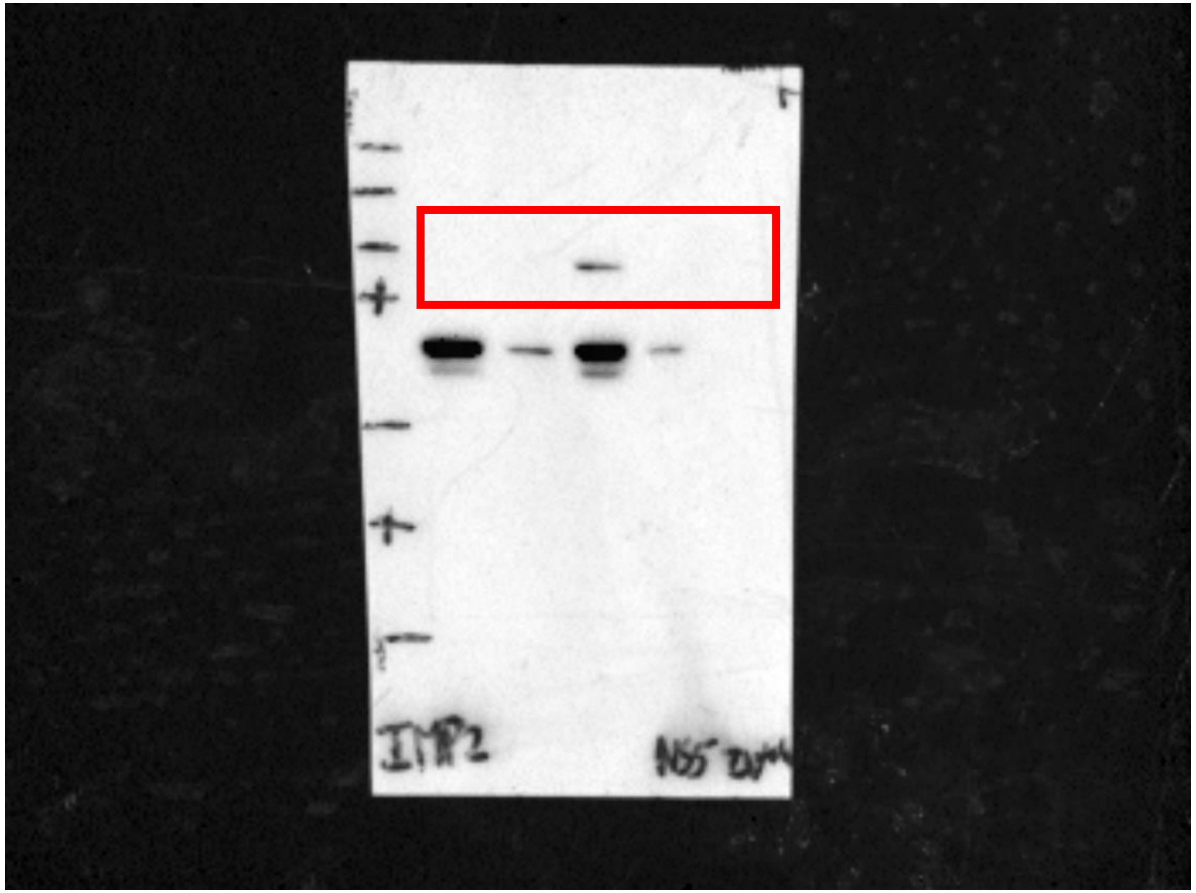

Anti-actin

1- Mock shNT
2- Mock shIGF2BP2
3- ZIKV shNT
4- ZIKV shIGF2BP2

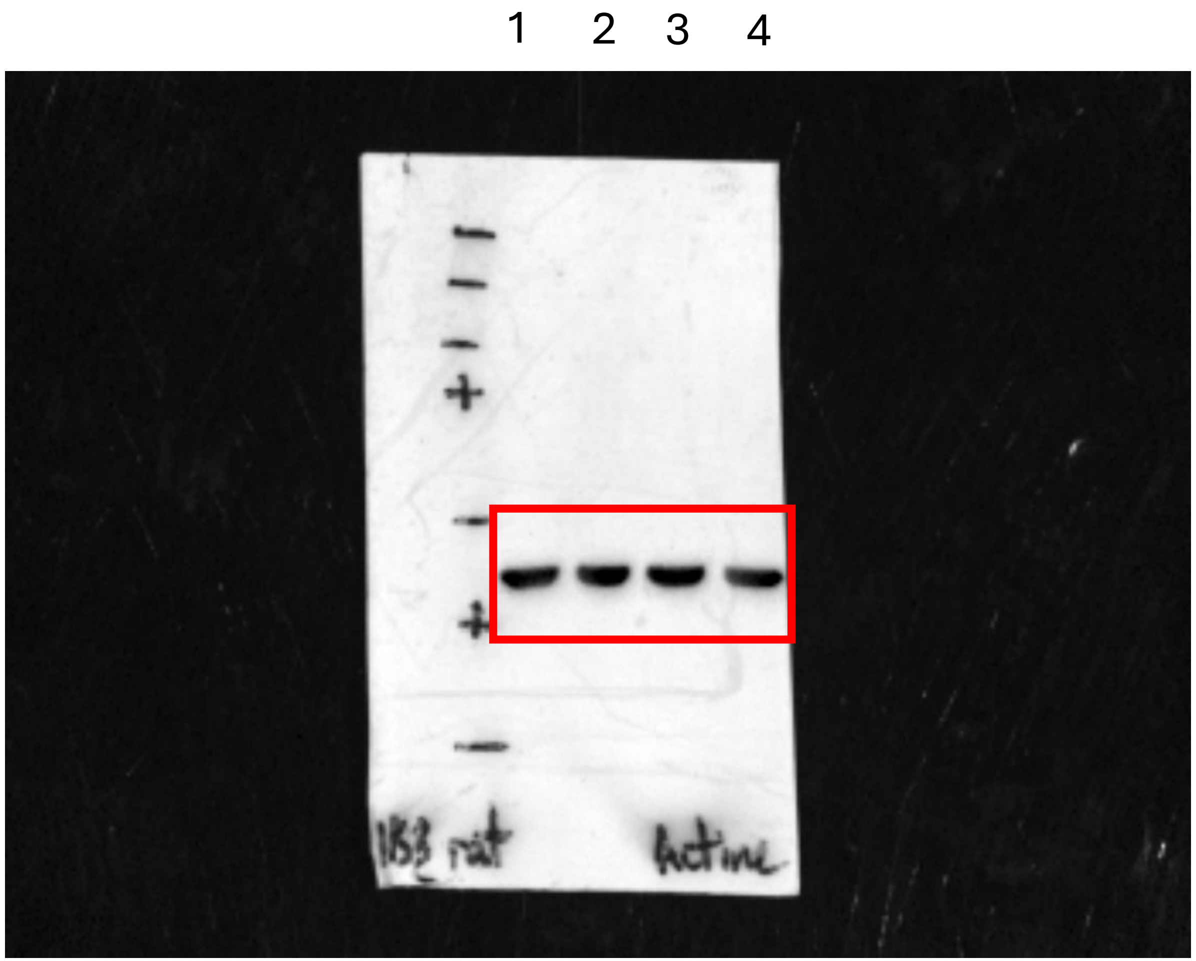

Figure 2D

Anti-IGF2BP2

3- NHA-hTERT shNT
4- NHA-hTERT shIGF2BP2

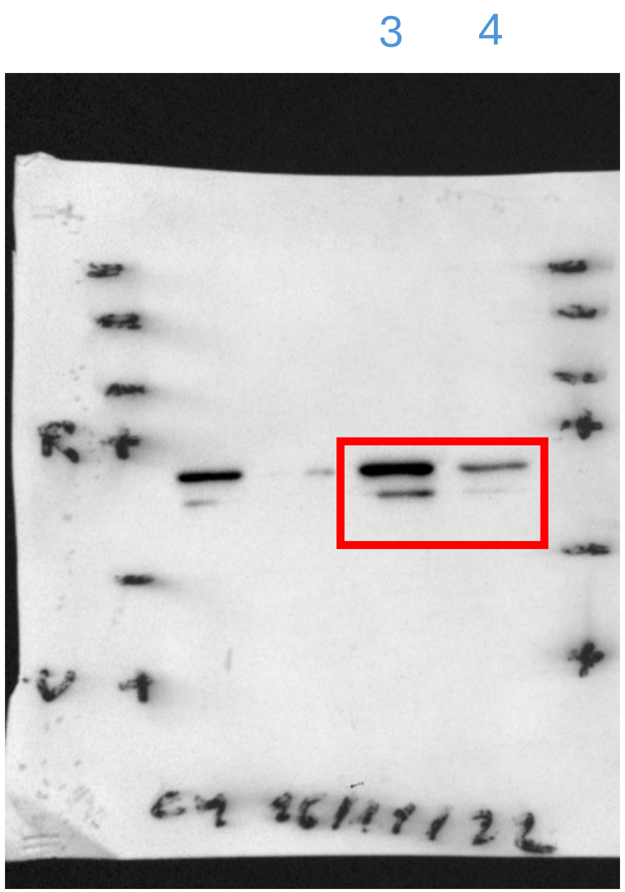

Anti-actin

3- NHA-hTERT shNT
4- NHA-hTERT shIGF2BP2

3 4

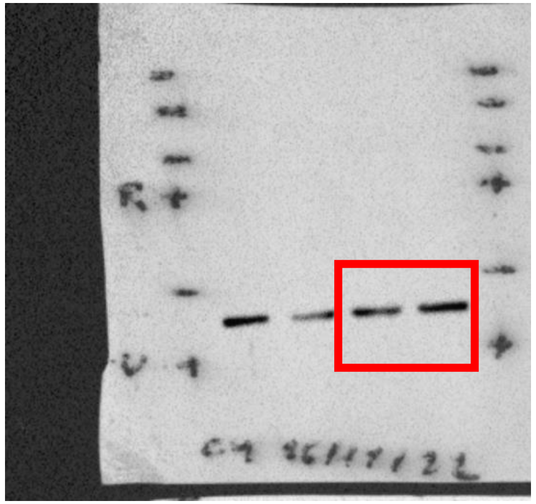

Figure 2E

Anti-IGF2BP2

1- JEG-3 shNT
2- JEG-3 shIGF2BP2

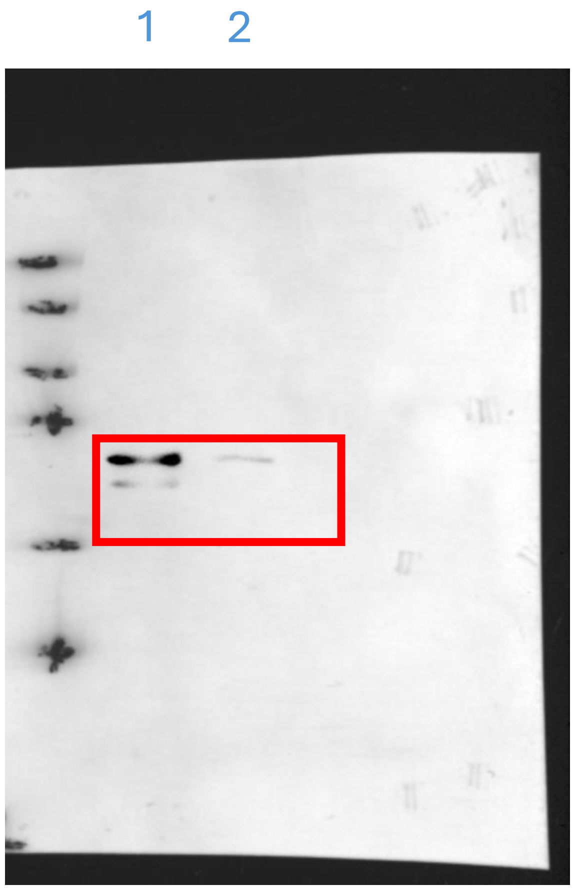

Anti-Actin

1- JEG-3 shNT
2- JEG-3 shIGF2BP2

1 2

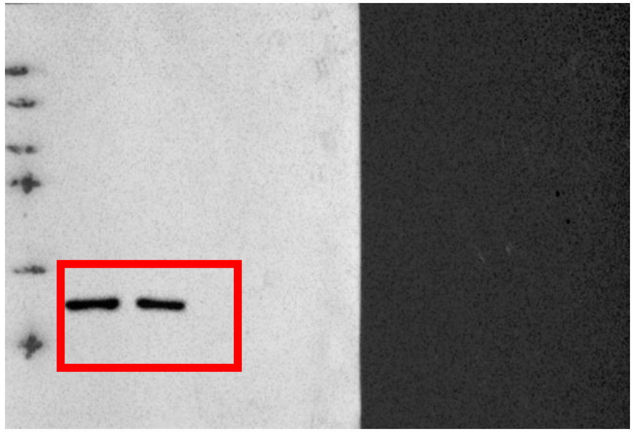

Supplement: Figure 2—source data 2. [file elife-94347-fig2-data2.zip › Figure 2-source data 2.pdf]
