# Supplementary figures and images for: Zika virus remodels and hijacks IGF2BP2 ribonucleoprotein complex to promote viral replication organelle biogenesis
Source: eLife. 2024 Nov 20;13:RP94347. doi: 10.7554/eLife.94347 (PMC11578589; doi:10.7554/eLife.94347)

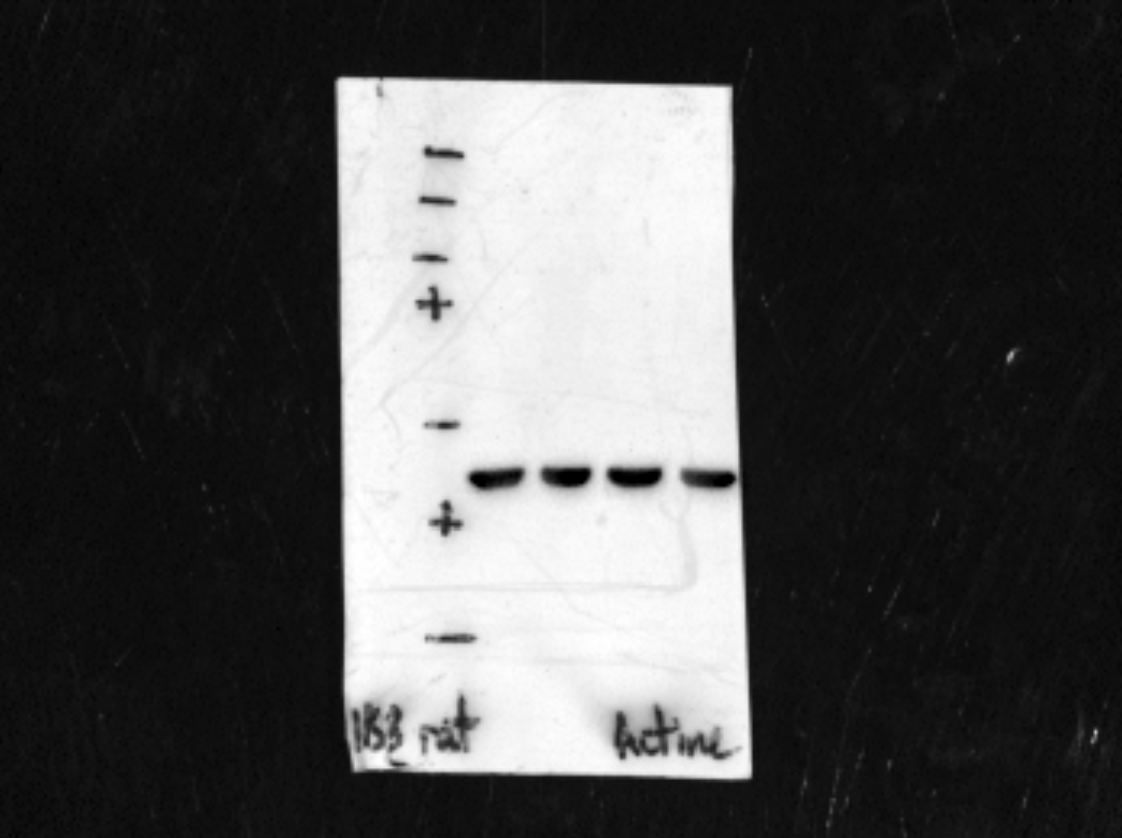

Supplement: Figure 2—source data 3. [file elife-94347-fig2-data3.zip › Figure 2A raw data/Actin/Merge_Actine.tif]

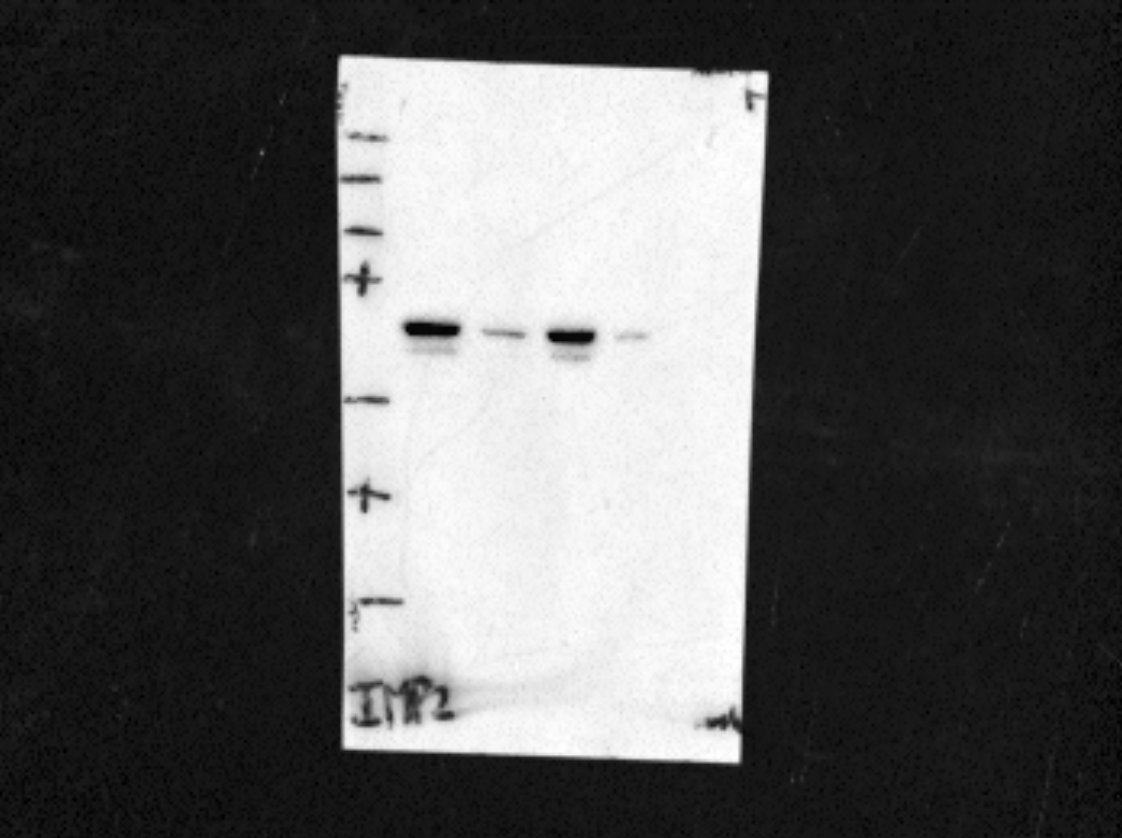

Supplement: Figure 2—source data 3. [file elife-94347-fig2-data3.zip › Figure 2A raw data/IGF2BP2/Merge_IGF2BP2.tif]

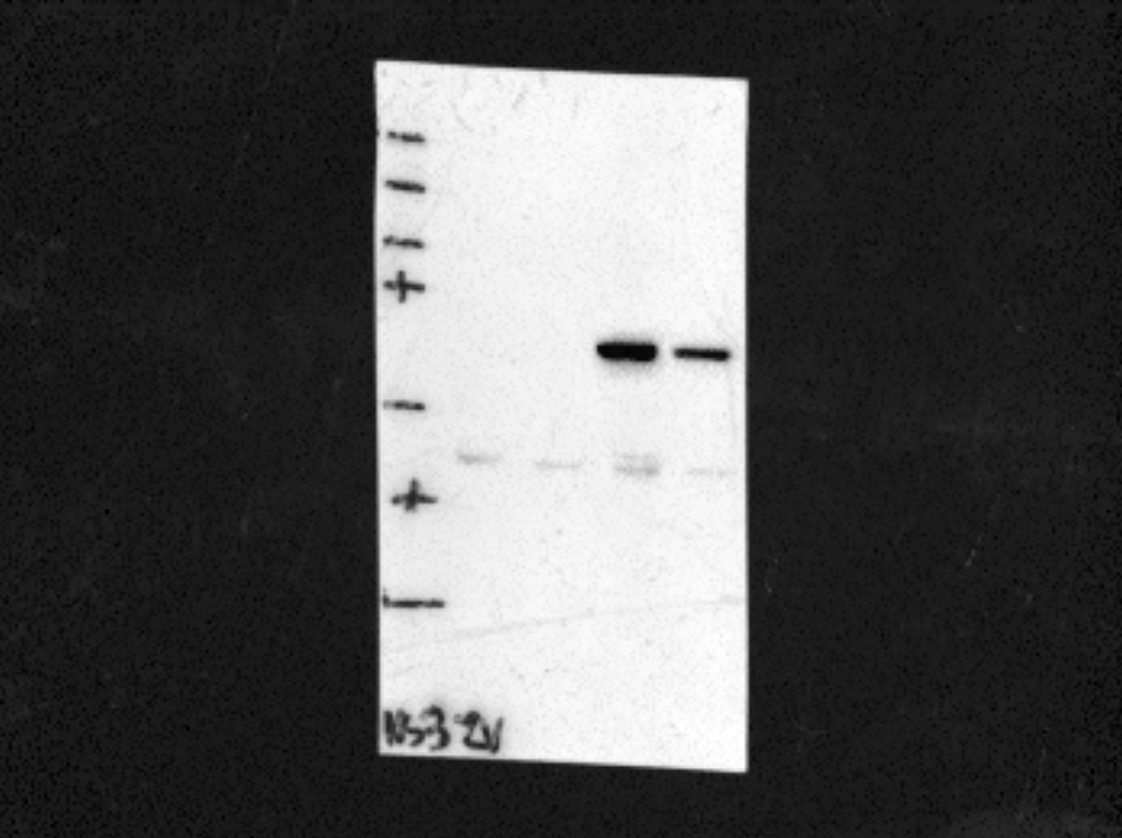

Supplement: Figure 2—source data 3. [file elife-94347-fig2-data3.zip › Figure 2A raw data/NS3/Merge_NS3 ZV.tif]

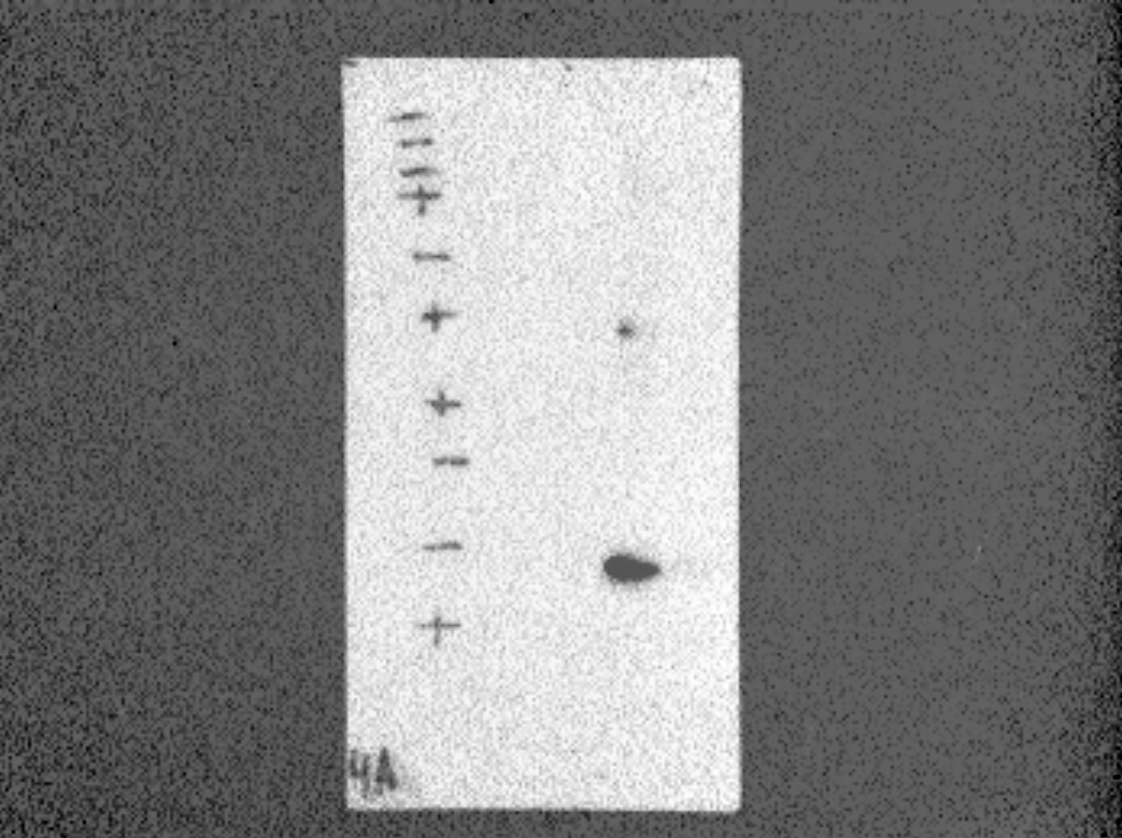

Supplement: Figure 2—source data 3. [file elife-94347-fig2-data3.zip › Figure 2A raw data/NS4A/Merge_NS4A ZV.tif]

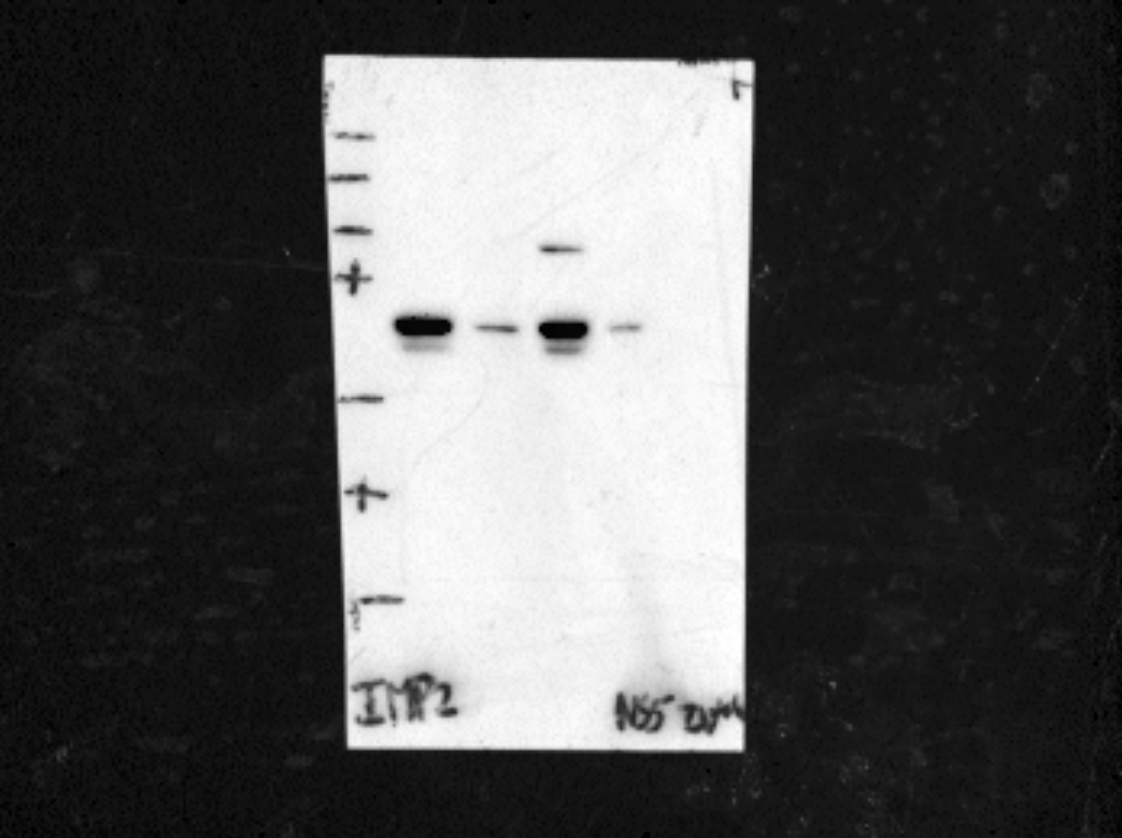

Supplement: Figure 2—source data 3. [file elife-94347-fig2-data3.zip › Figure 2A raw data/NS5/Merge_NS5 ZV.tif]

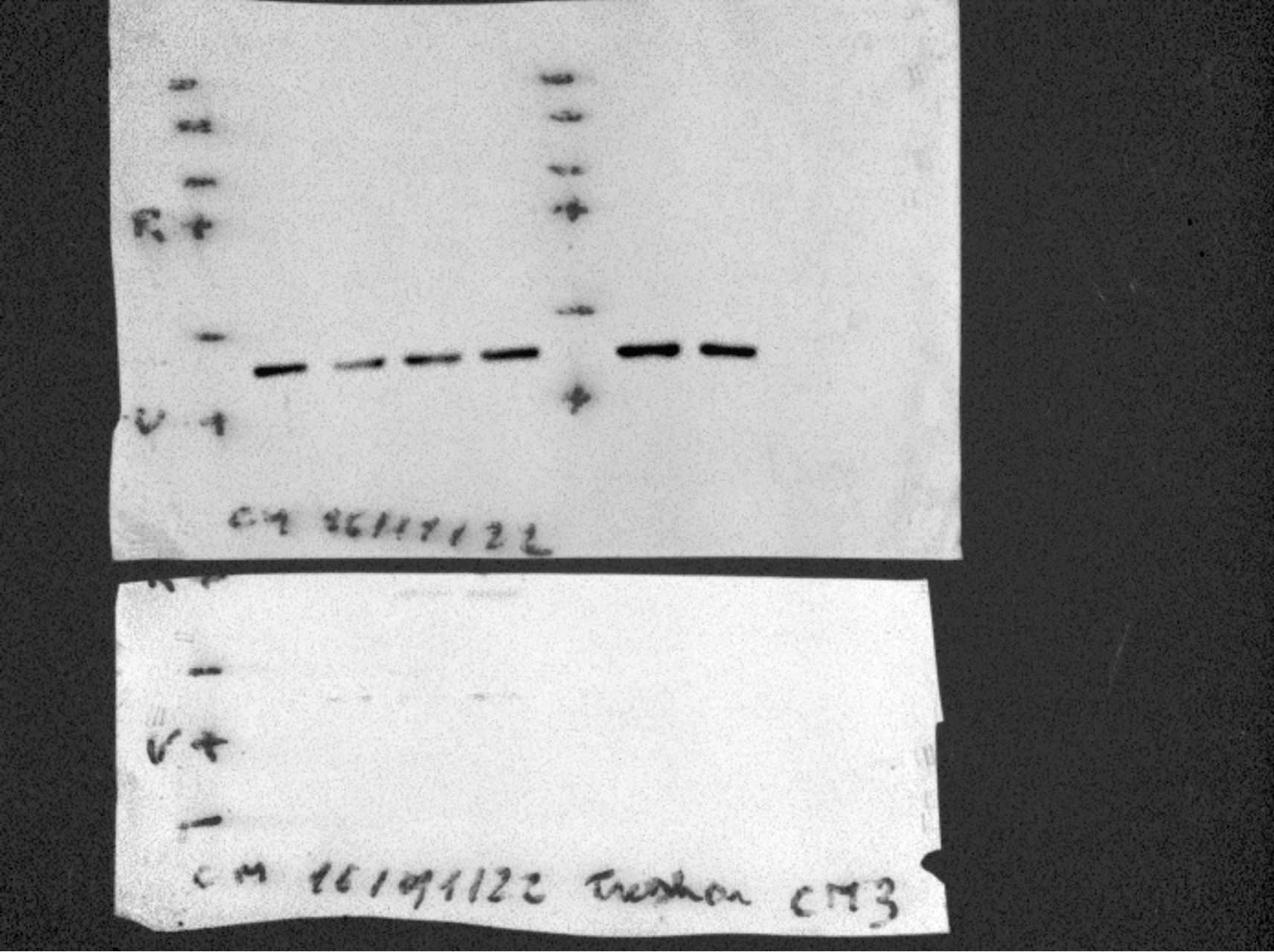

Supplement: Figure 2—source data 3. [file elife-94347-fig2-data3.zip › figure 2D raw data/Actin/Melany Juarez 2022-11-18 14hr 51min_Exposure_15.5sec+Melany Juarez 2022-11-18 15hr 00min MW.tif]

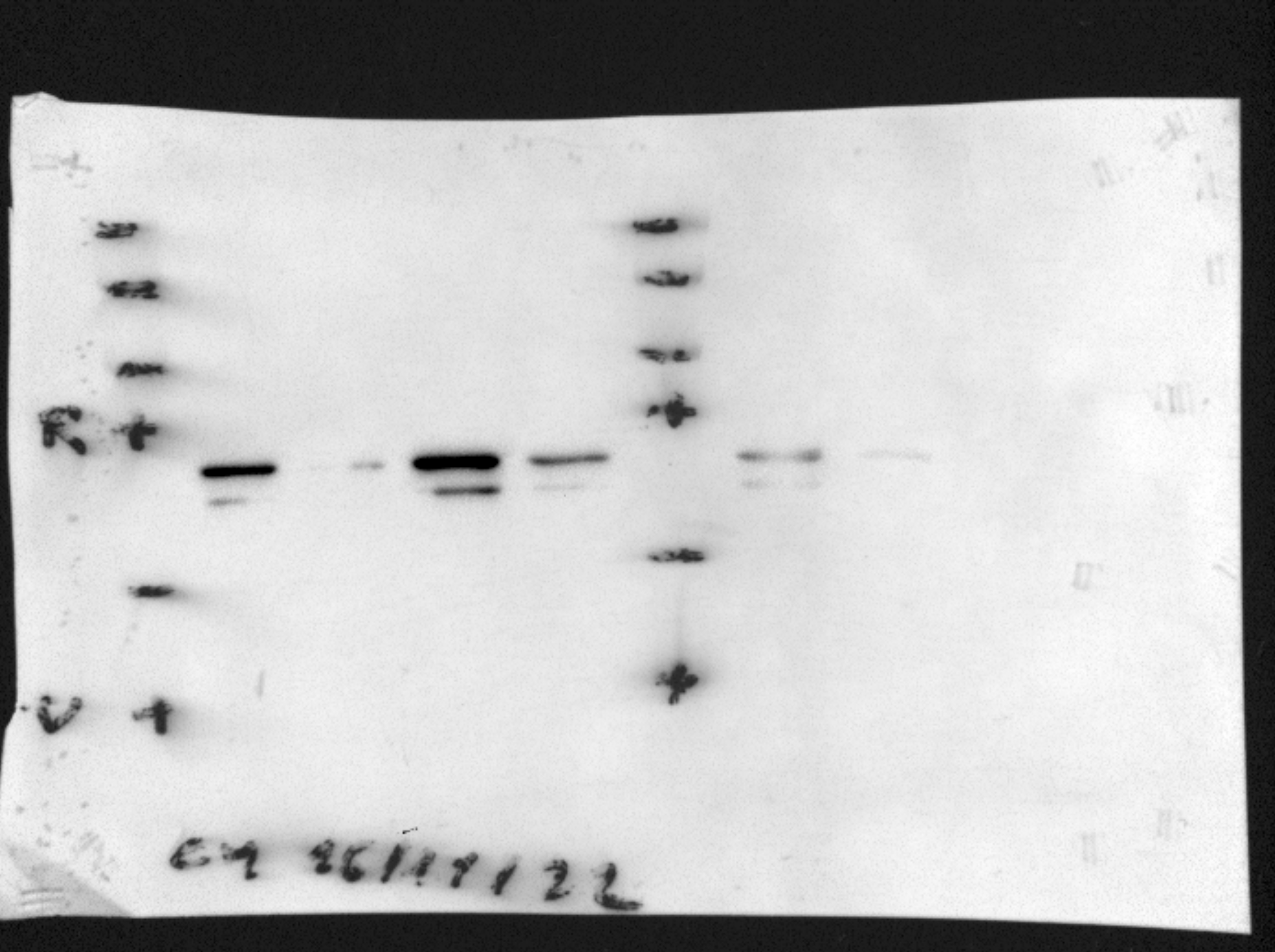

Supplement: Figure 2—source data 3. [file elife-94347-fig2-data3.zip › figure 2D raw data/IGF2BP2/merge.tif]

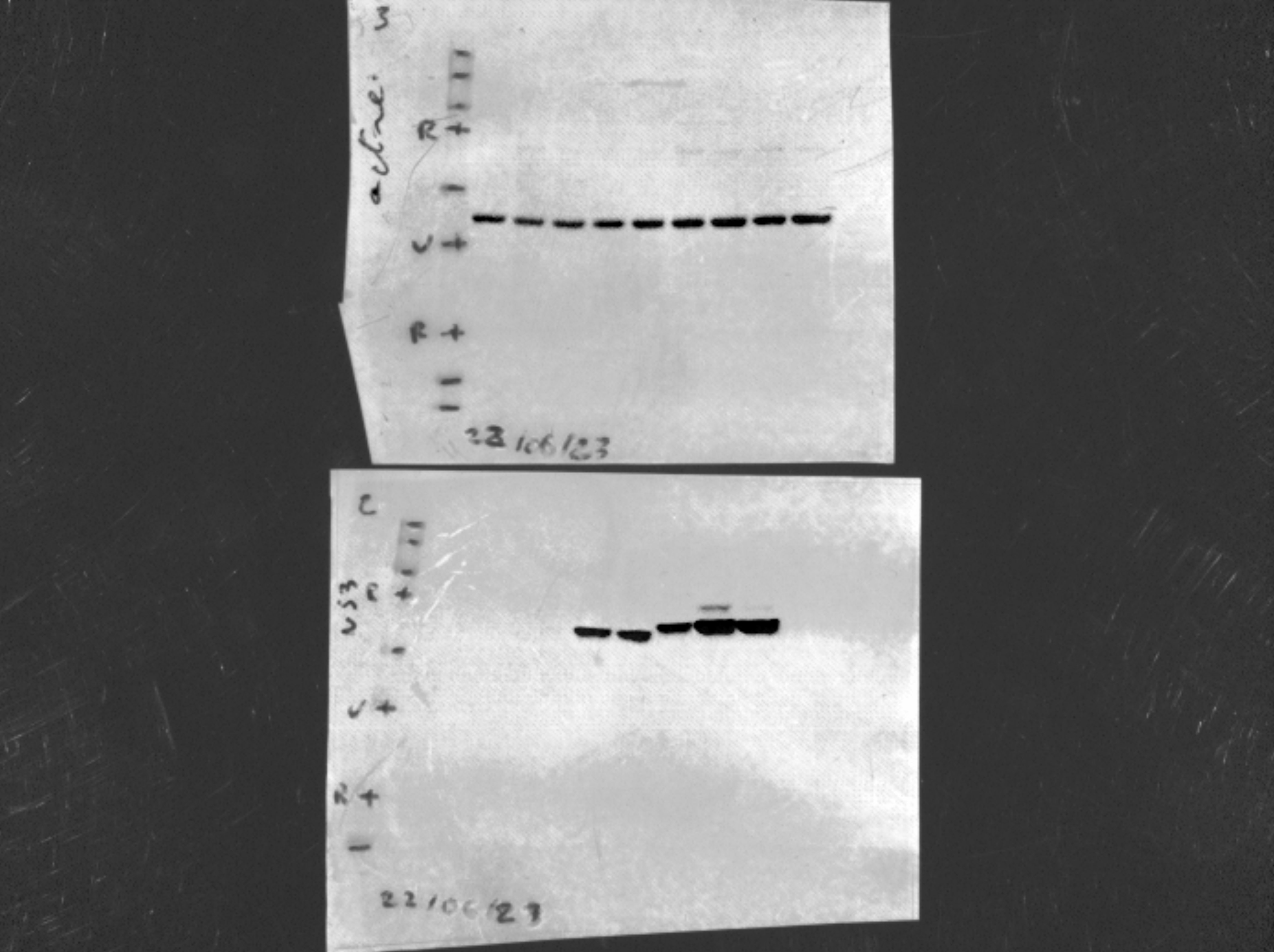

Supplement: Figure 4—source data 3. [file elife-94347-fig4-data3.zip › figure 4A raw data/Actin/merge.tif]

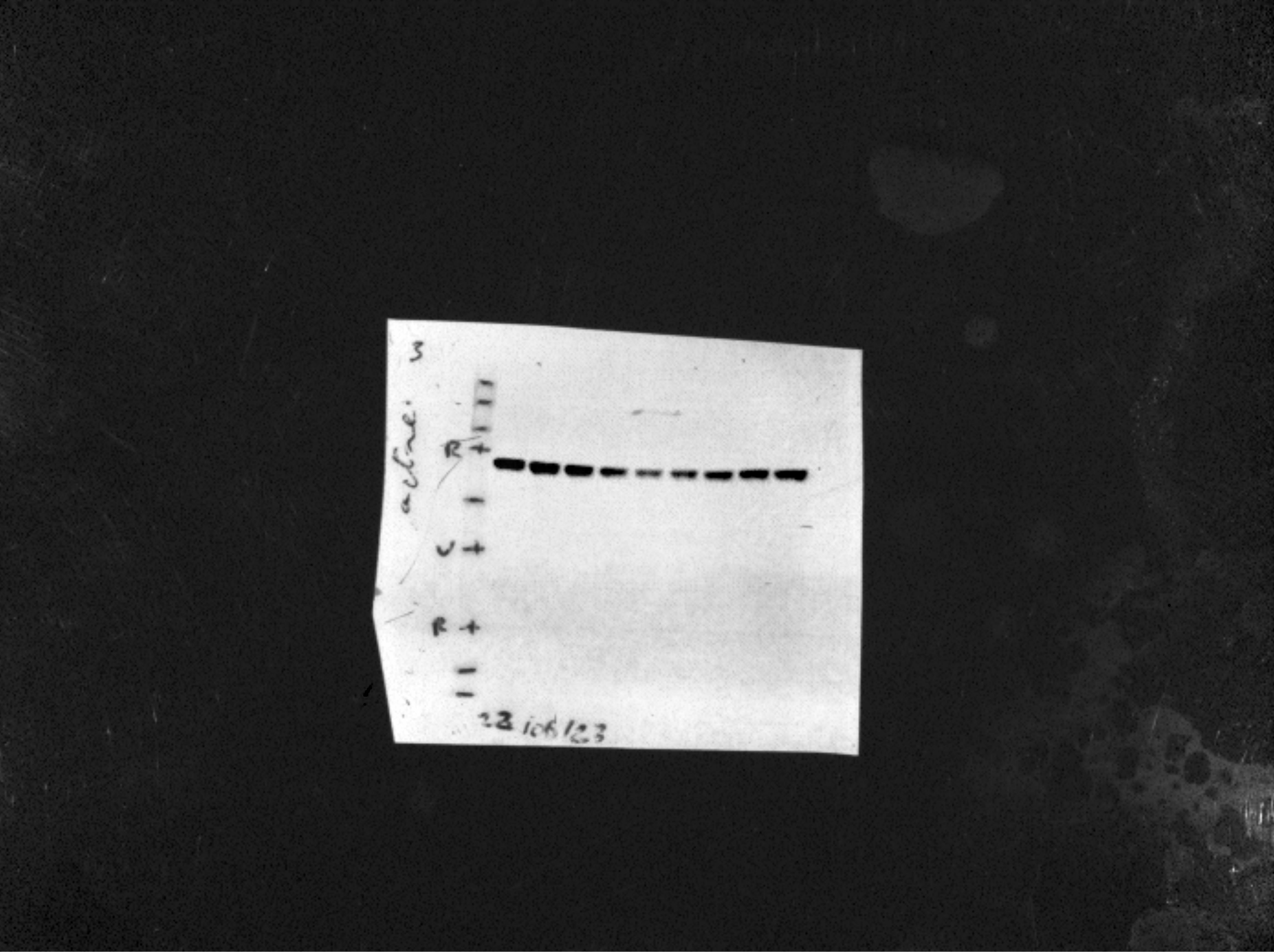

Supplement: Figure 4—source data 3. [file elife-94347-fig4-data3.zip › figure 4A raw data/DDX3/merge.tif]

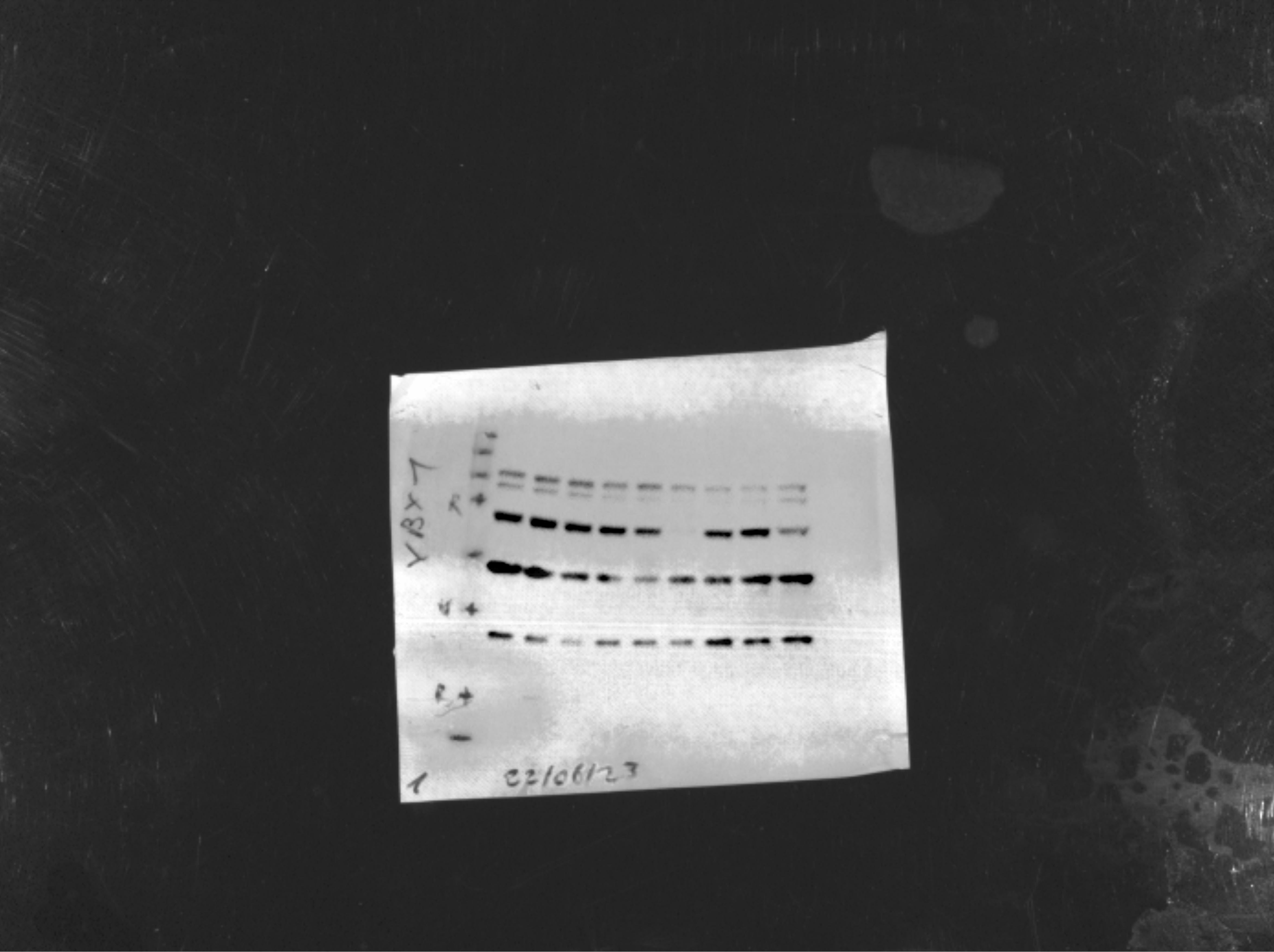

Supplement: Figure 4—source data 3. [file elife-94347-fig4-data3.zip › figure 4A raw data/DDX5/merge.tif]

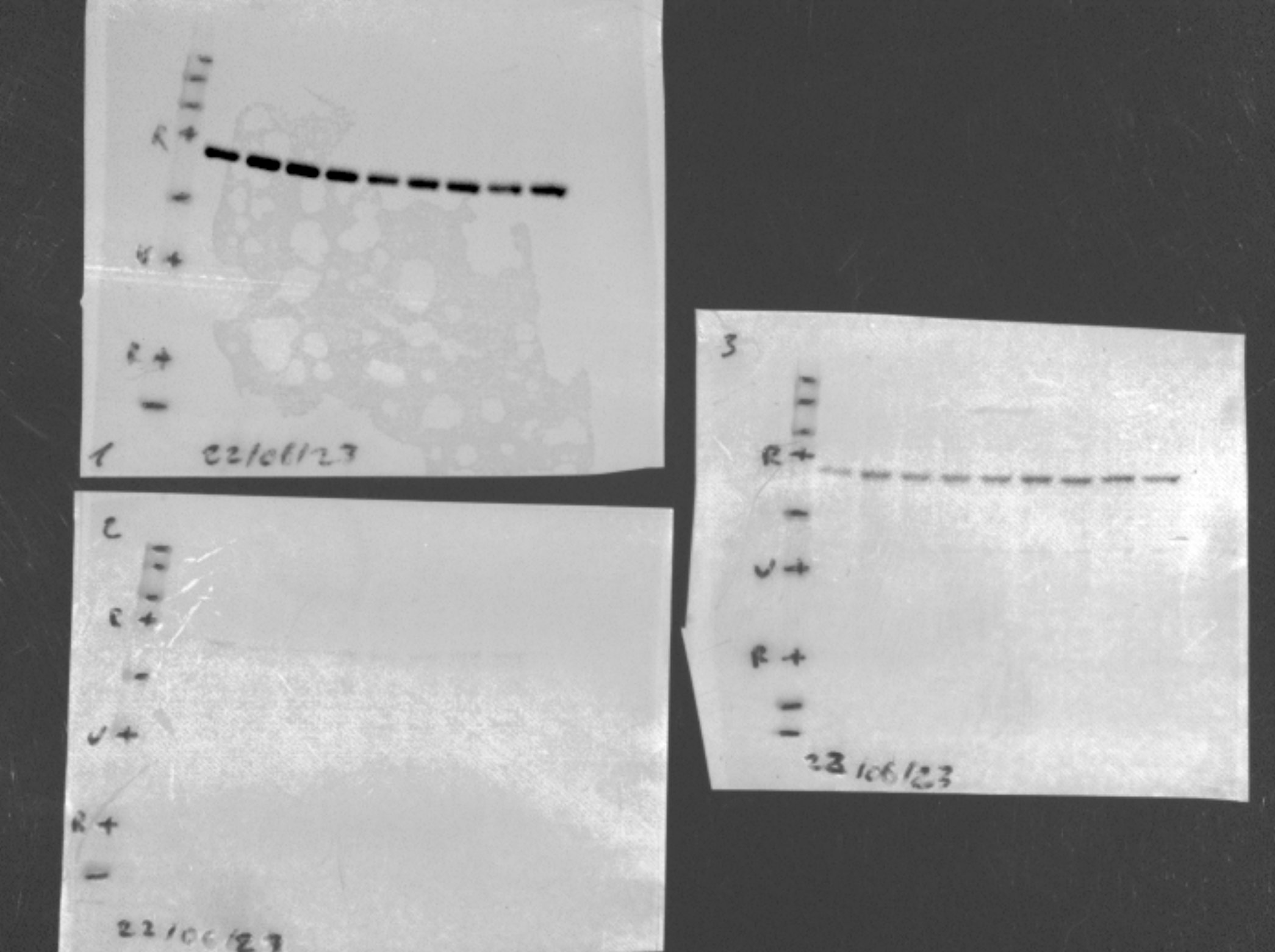

Supplement: Figure 4—source data 3. [file elife-94347-fig4-data3.zip › figure 4A raw data/IGF2BP1/Melany Juarez 2023-06-24 17hr 49min_Exposure_3.4sec+Melany Juarez 2023-06-24 17hr 51min MW.tif]

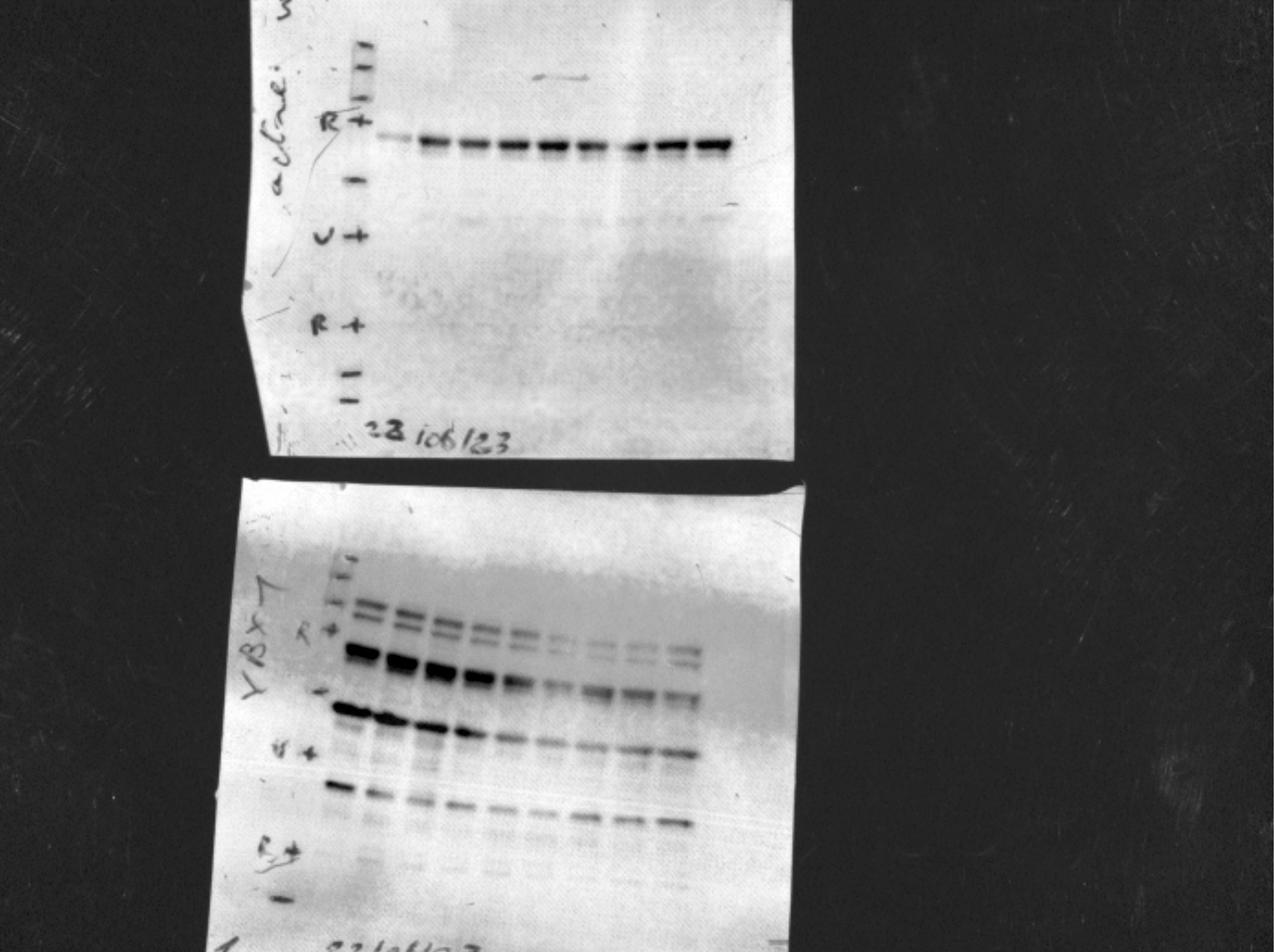

Supplement: Figure 4—source data 3. [file elife-94347-fig4-data3.zip › figure 4A raw data/IGF2BP2/merge.tif]

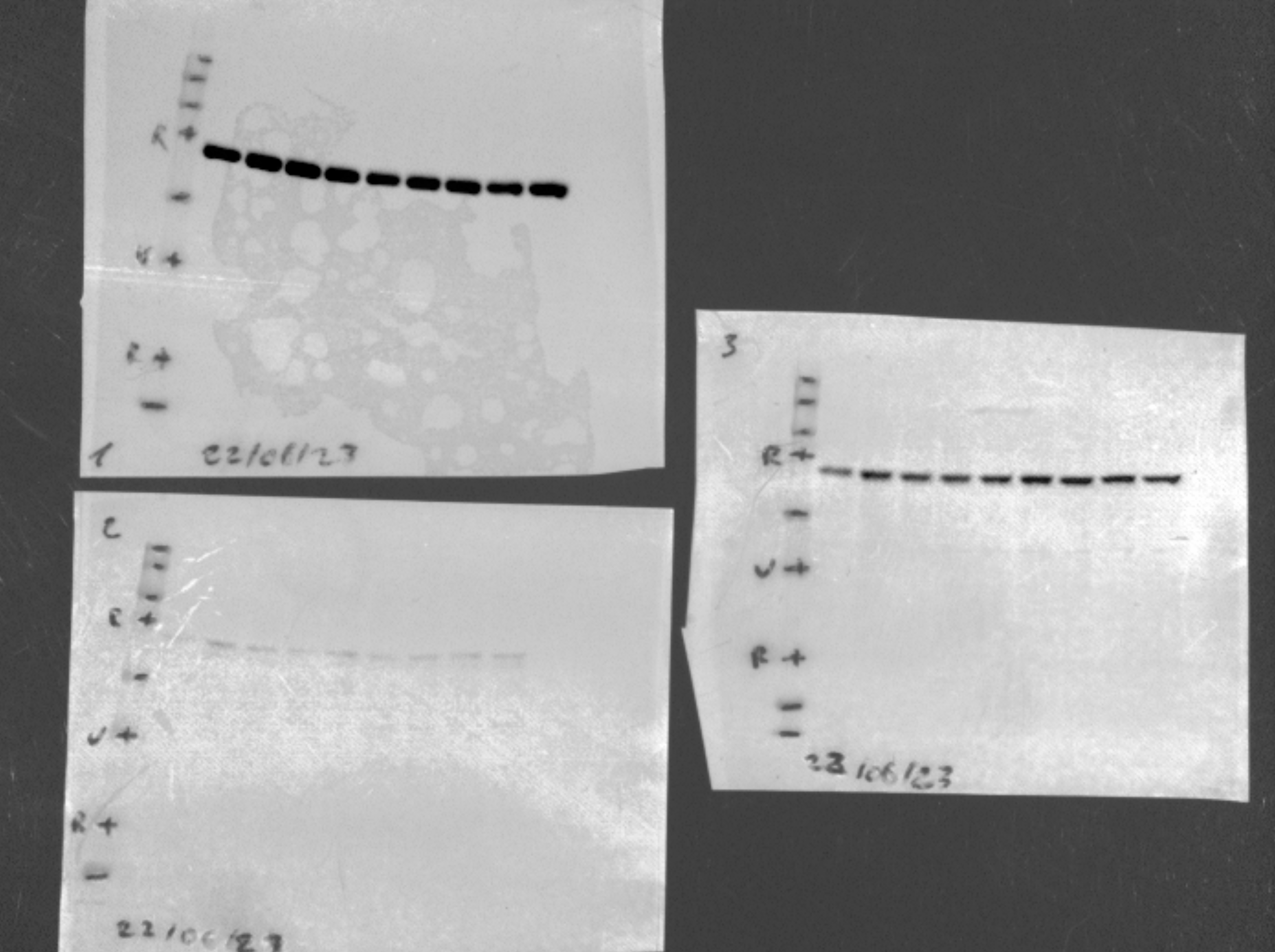

Supplement: Figure 4—source data 3. [file elife-94347-fig4-data3.zip › figure 4A raw data/IGF2BP3/merge.tif]

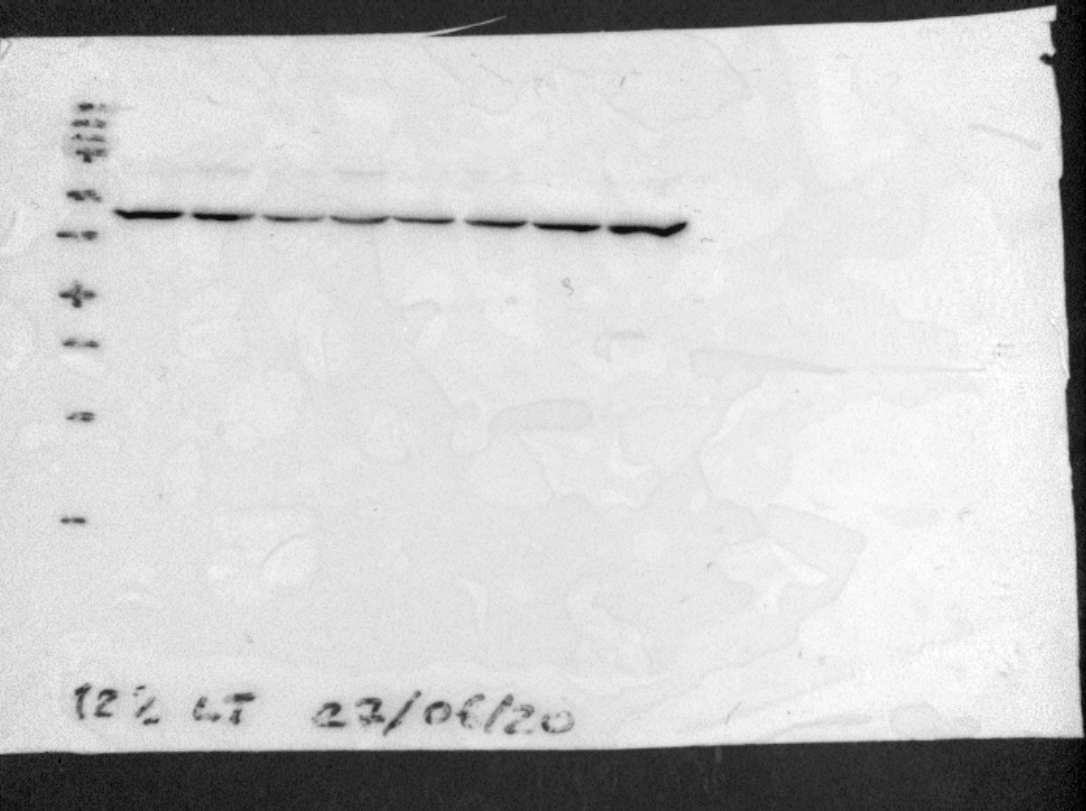

Supplement: Figure 4—source data 3. [file elife-94347-fig4-data3.zip › figure 4C raw data/Actin/actin merge.tif]

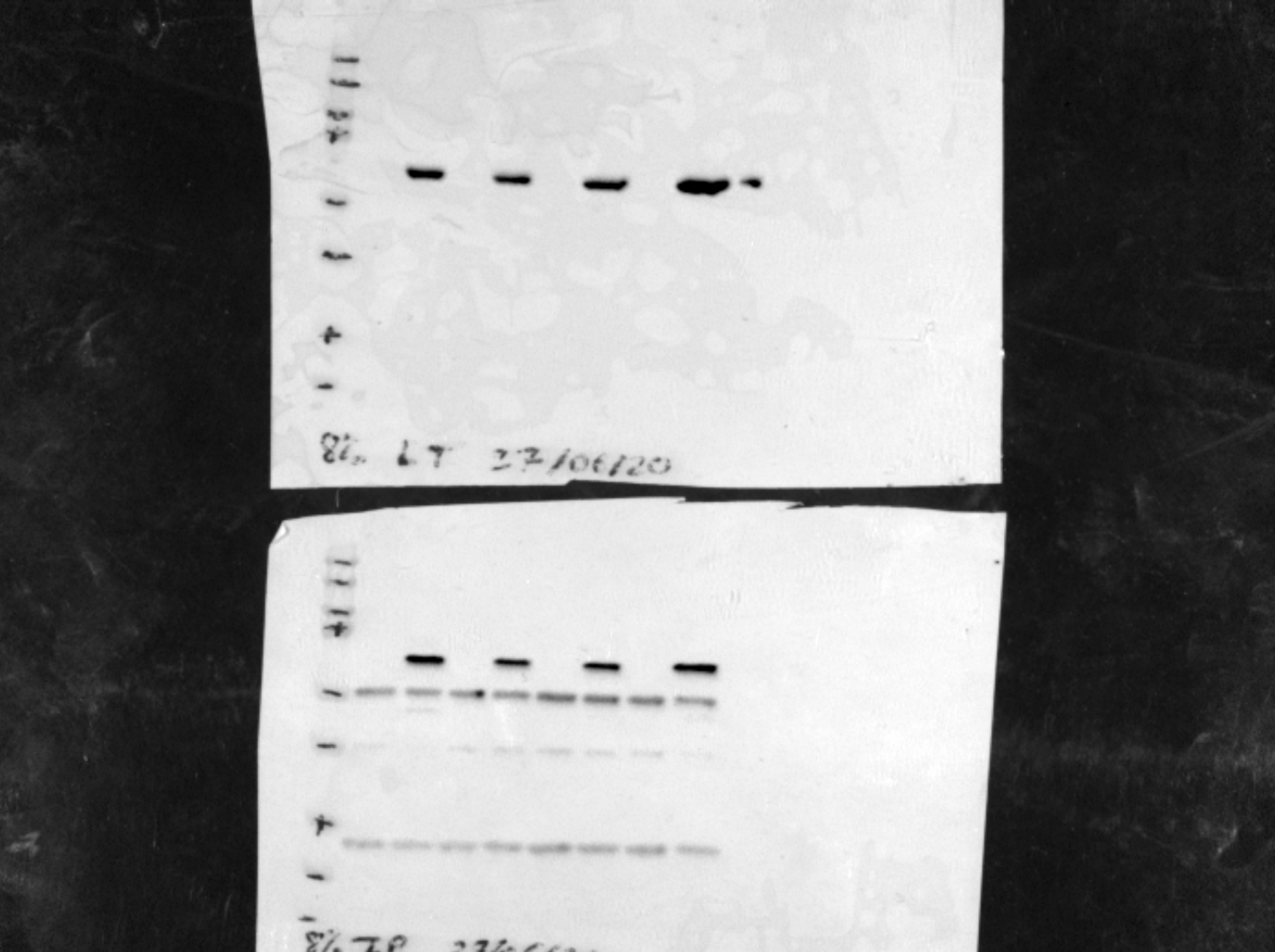

Supplement: Figure 4—source data 3. [file elife-94347-fig4-data3.zip › figure 4C raw data/HA-cell extracts/ha merge.tif]

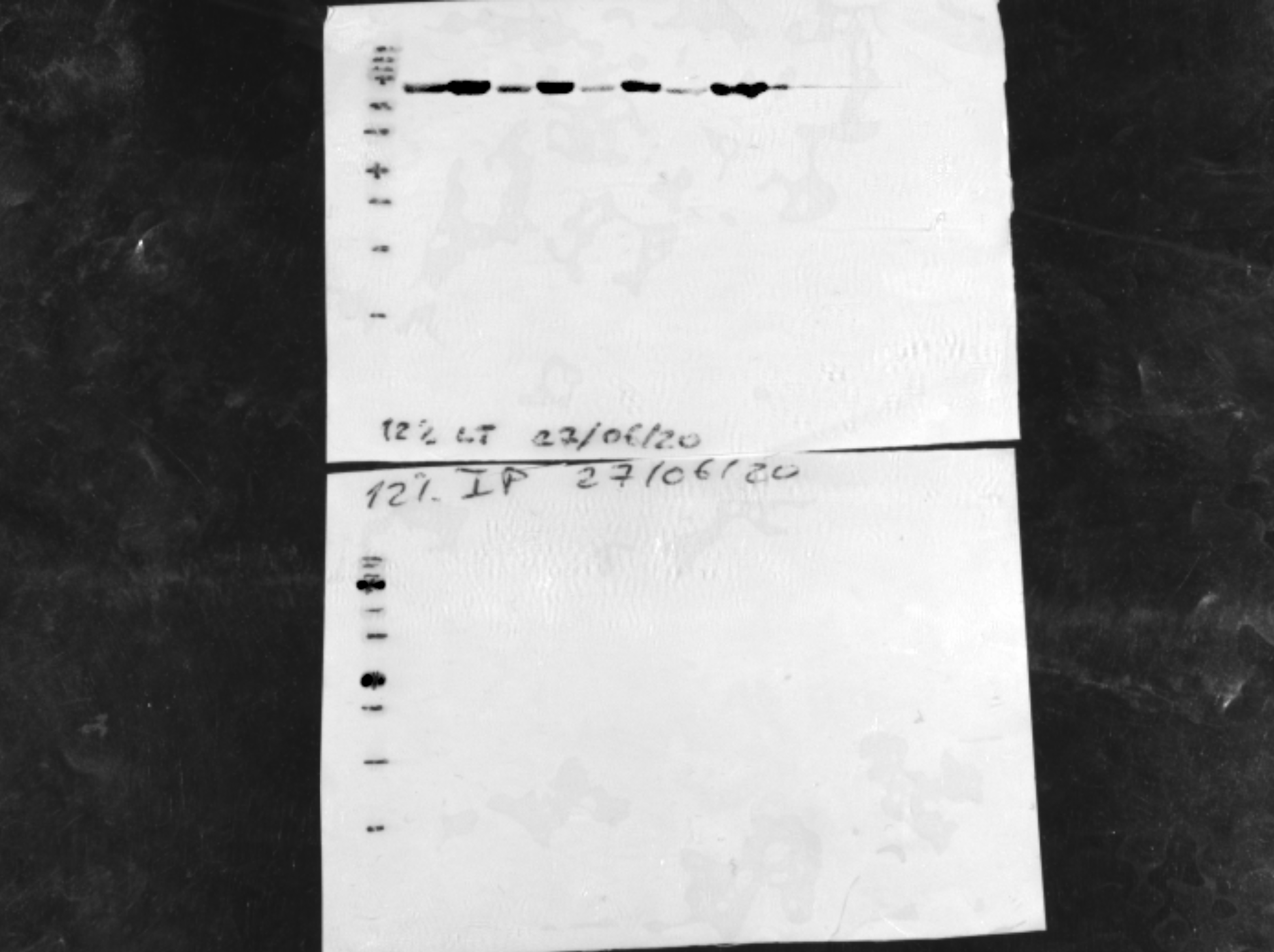

Supplement: Figure 4—source data 3. [file elife-94347-fig4-data3.zip › figure 4C raw data/IGF2BP2-cell extracts/igf2bp2 merge.tif]

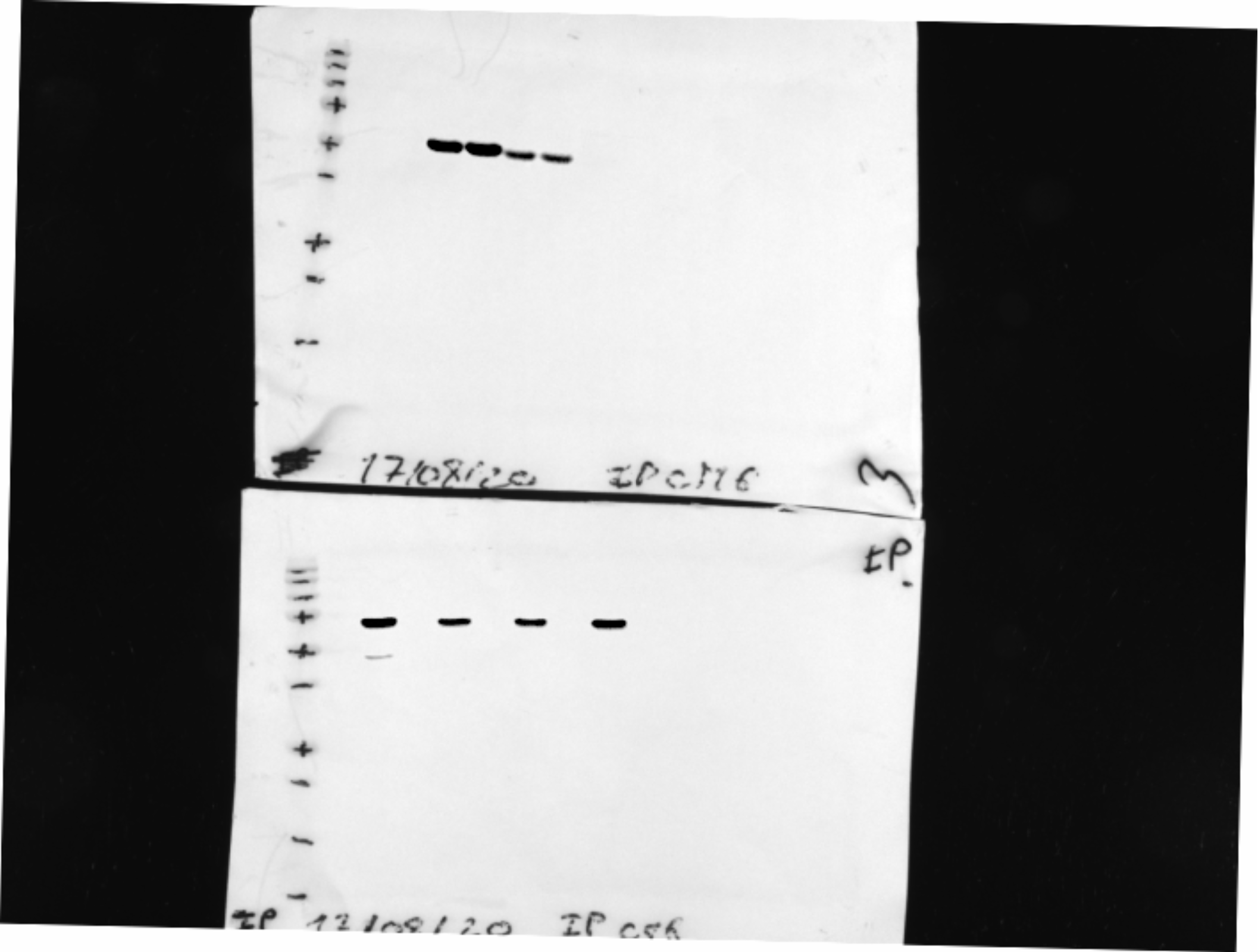

Supplement: Figure 4—source data 3. [file elife-94347-fig4-data3.zip › figure 4C raw data/IGF2BP2-IP/Mazeaud, Clement 2020-08-20 14hr 35min MERGE.tif]

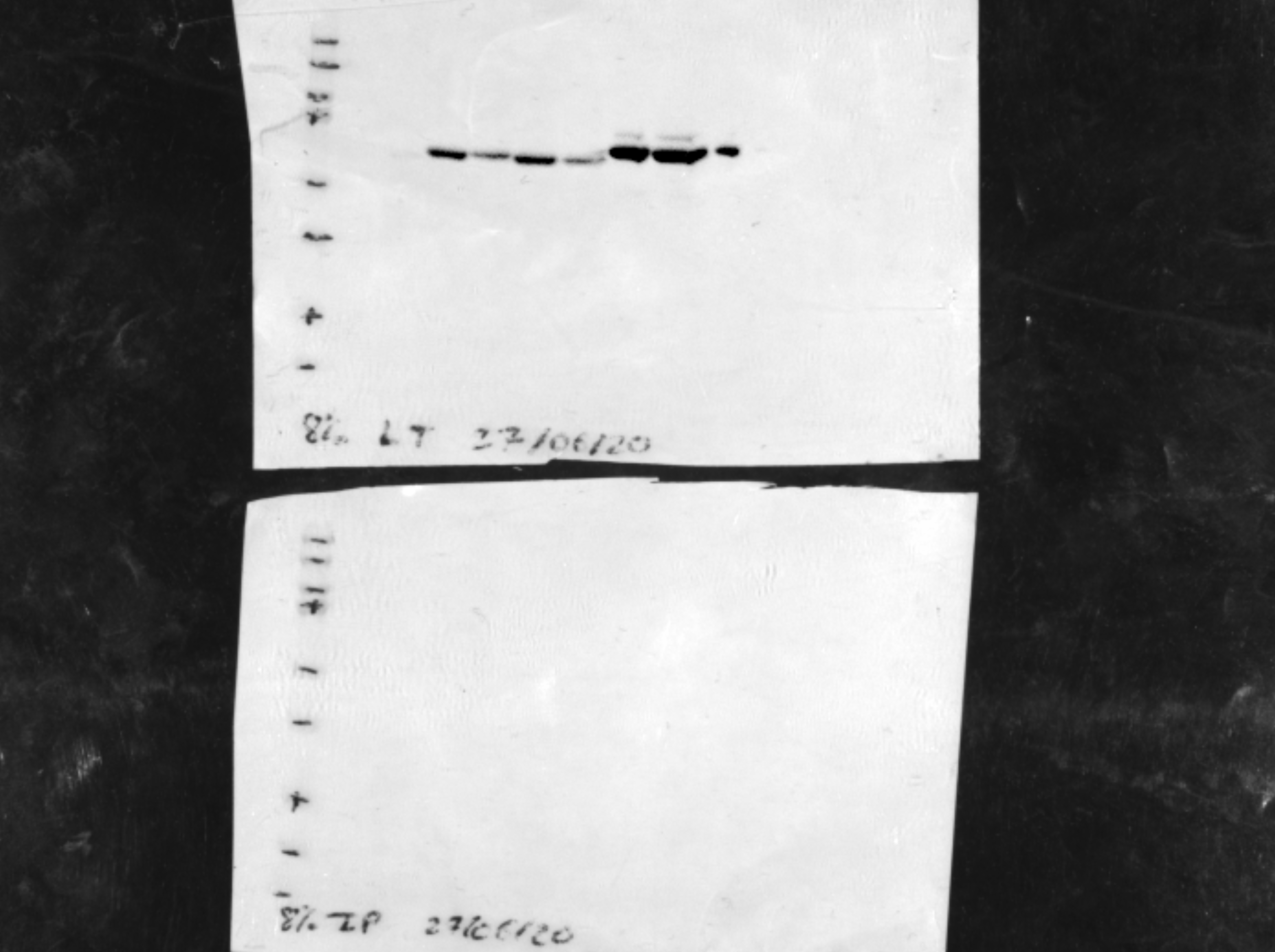

Supplement: Figure 4—source data 3. [file elife-94347-fig4-data3.zip › figure 4C raw data/NS3-cell extracts/merge.tif]

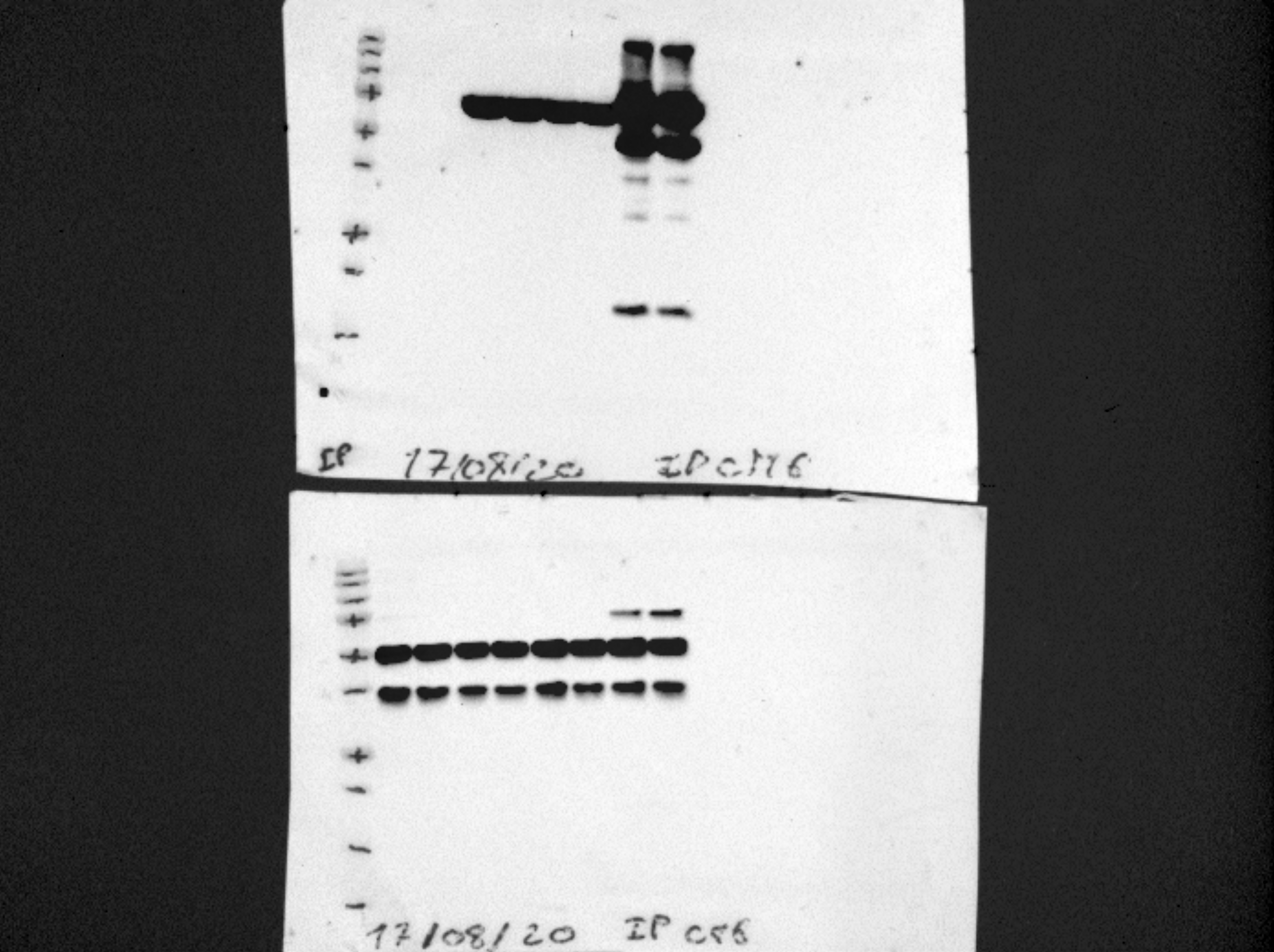

Supplement: Figure 4—source data 3. [file elife-94347-fig4-data3.zip › figure 4C raw data/NS3-IP/merge IP.tif]

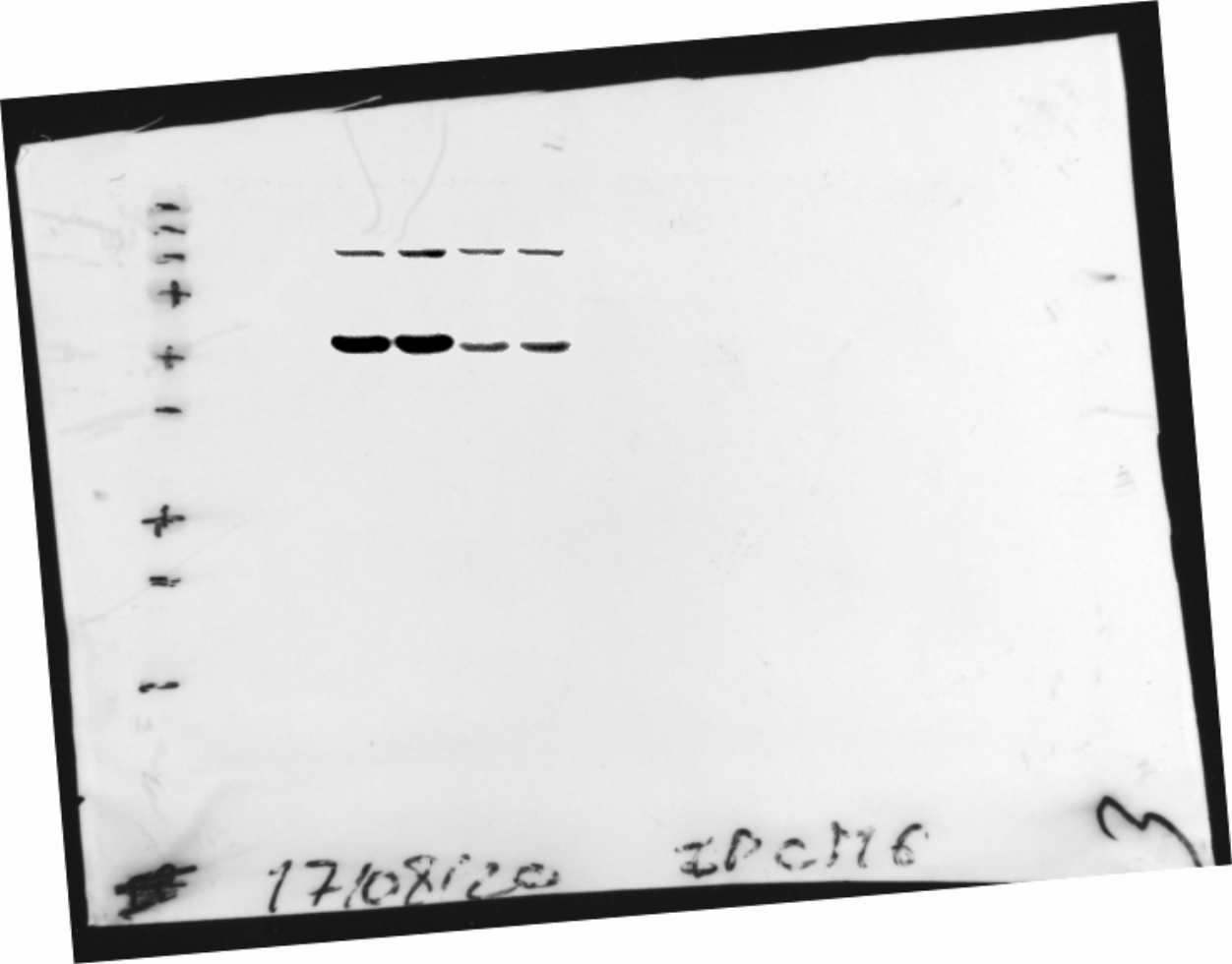

Supplement: Figure 4—source data 3. [file elife-94347-fig4-data3.zip › figure 4C raw data/NS5 cell extracts/Mazeaud, Clement 2020-08-22 12hr 42min MERGE.tif]

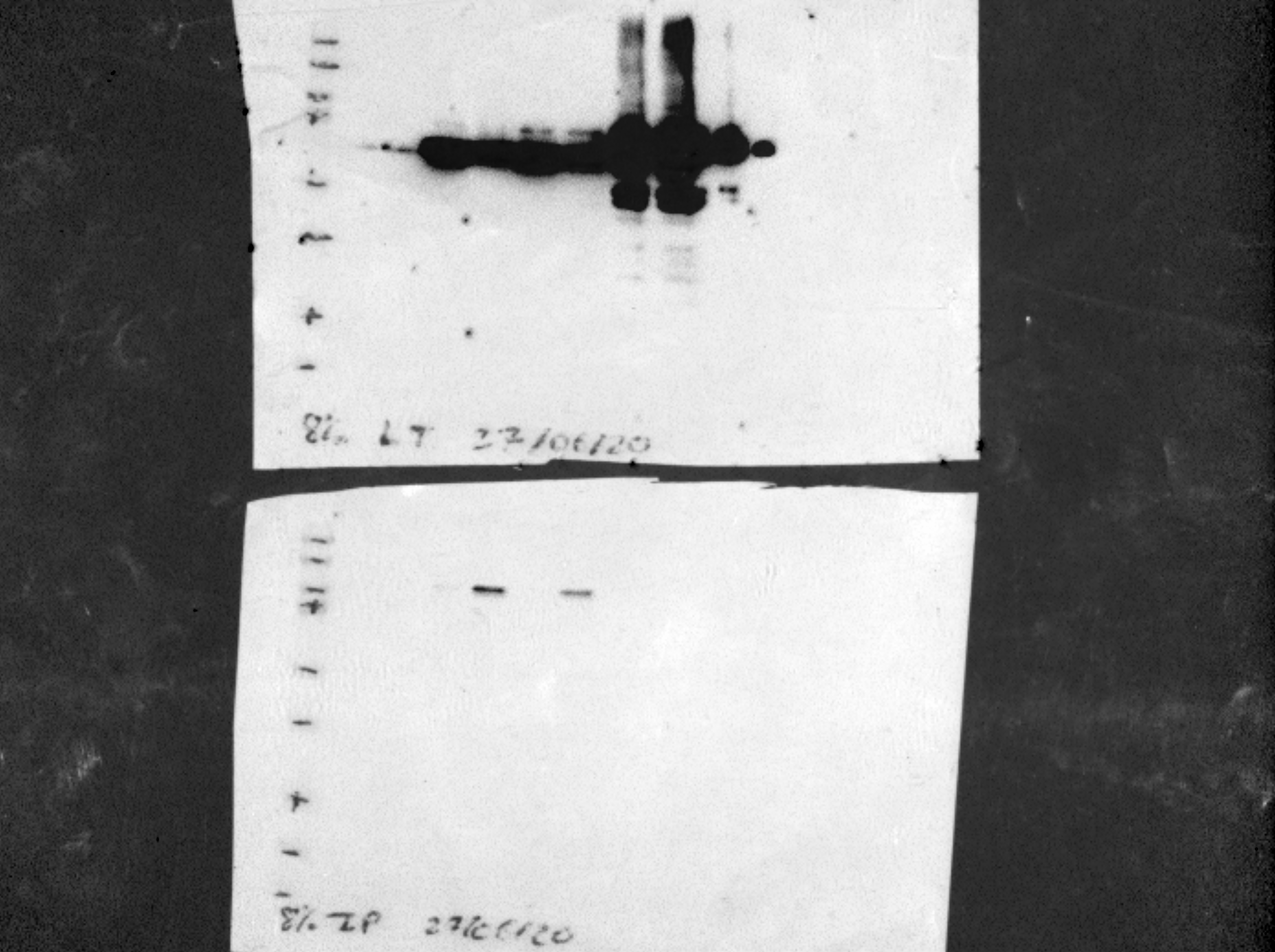

Supplement: Figure 4—source data 3. [file elife-94347-fig4-data3.zip › figure 4C raw data/NS5-IP/merge LT.tif]

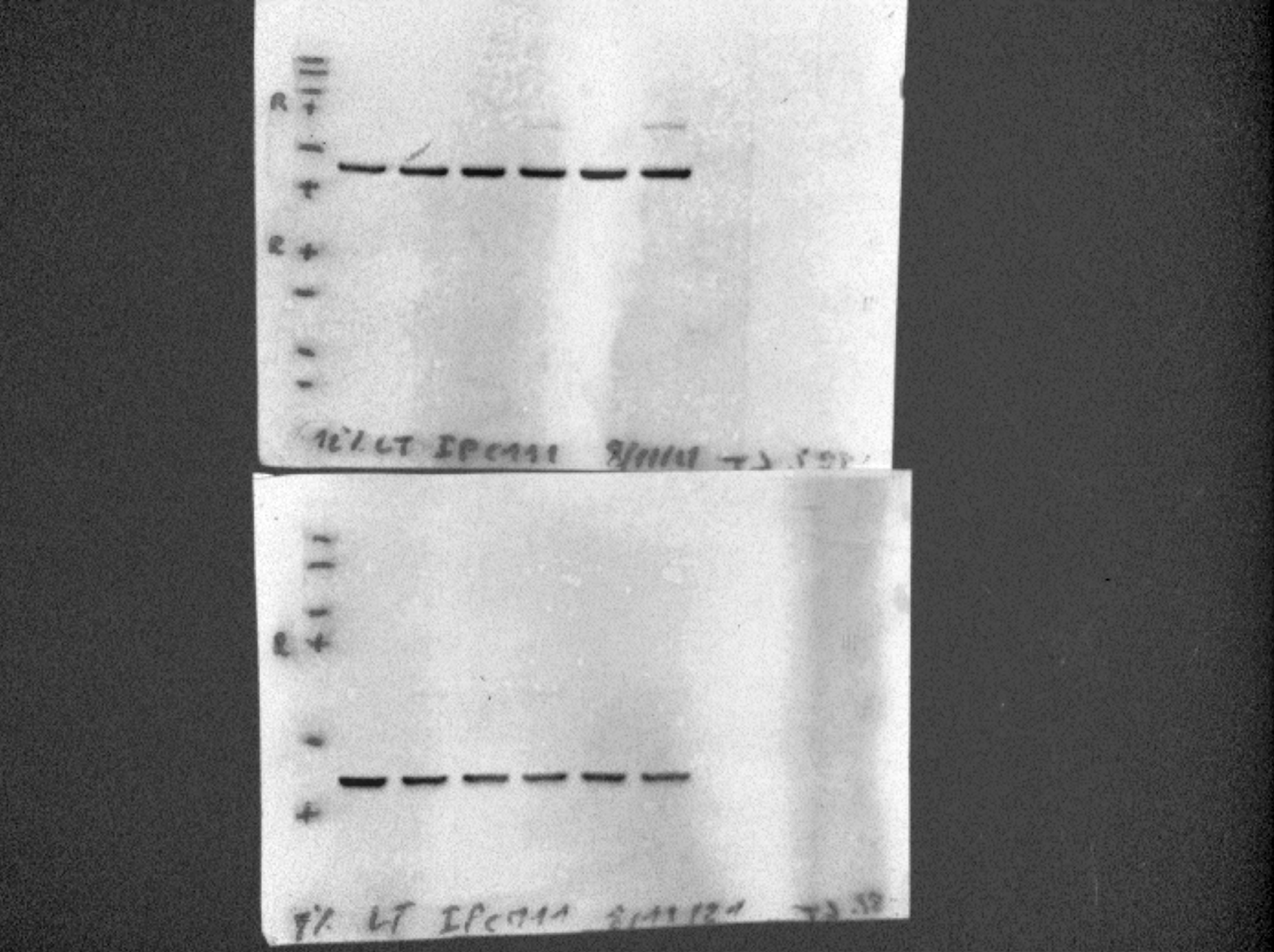

Supplement: Figure 8—source data 3. [file elife-94347-fig8-data3.zip › figure 8A raw data/Actin/merge.tif]

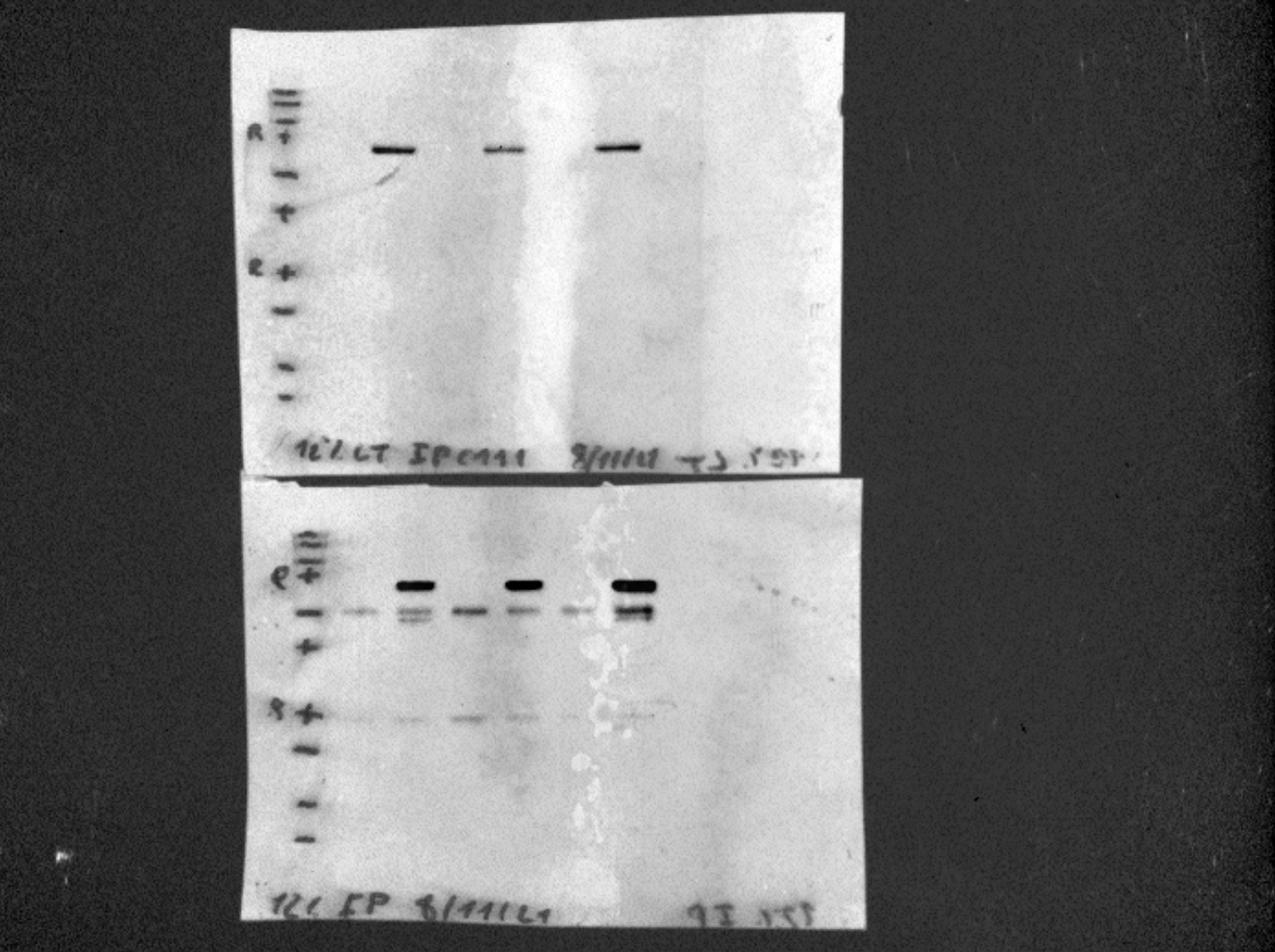

Supplement: Figure 8—source data 3. [file elife-94347-fig8-data3.zip › figure 8A raw data/HA/cell extracts/Mazeaud, Clement 2021-11-09 16hr 56min Merge.tif]

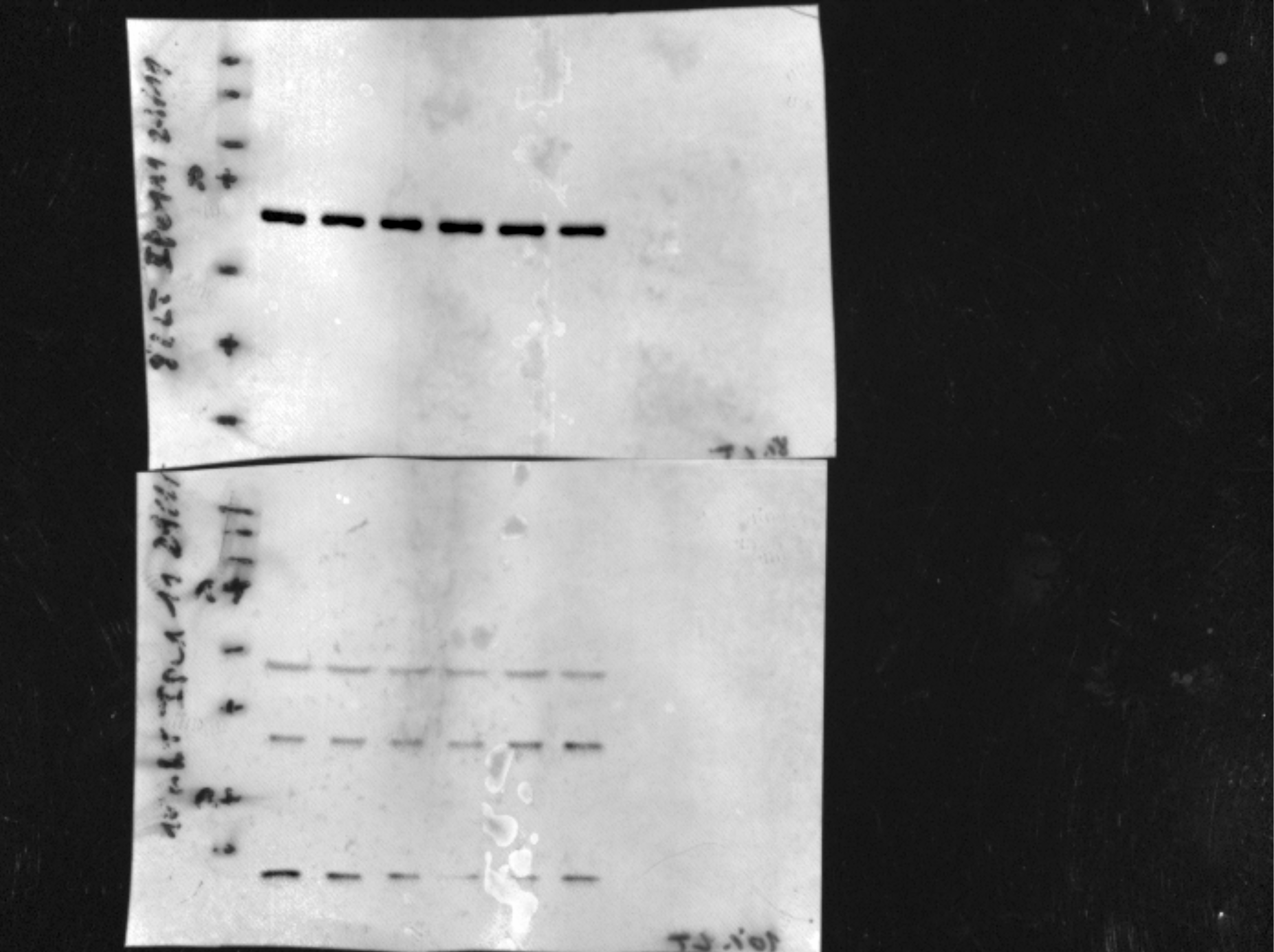

Supplement: Figure 8—source data 3. [file elife-94347-fig8-data3.zip › figure 8A raw data/IGF2BP1/cell extracts/merge LT.tif]

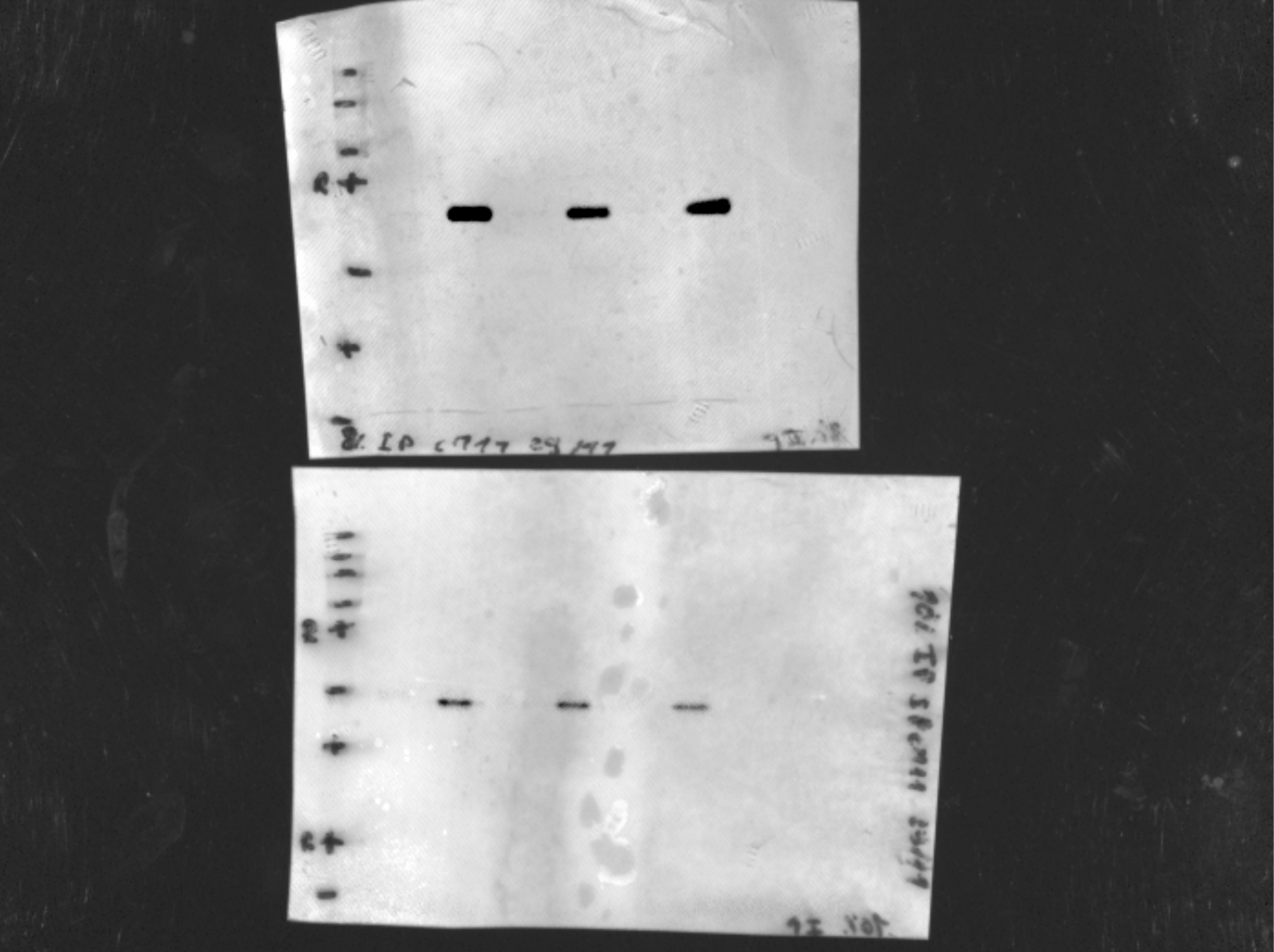

Supplement: Figure 8—source data 3. [file elife-94347-fig8-data3.zip › figure 8A raw data/IGF2BP1/IP anti-HA/merge IMP1 IP.tif]

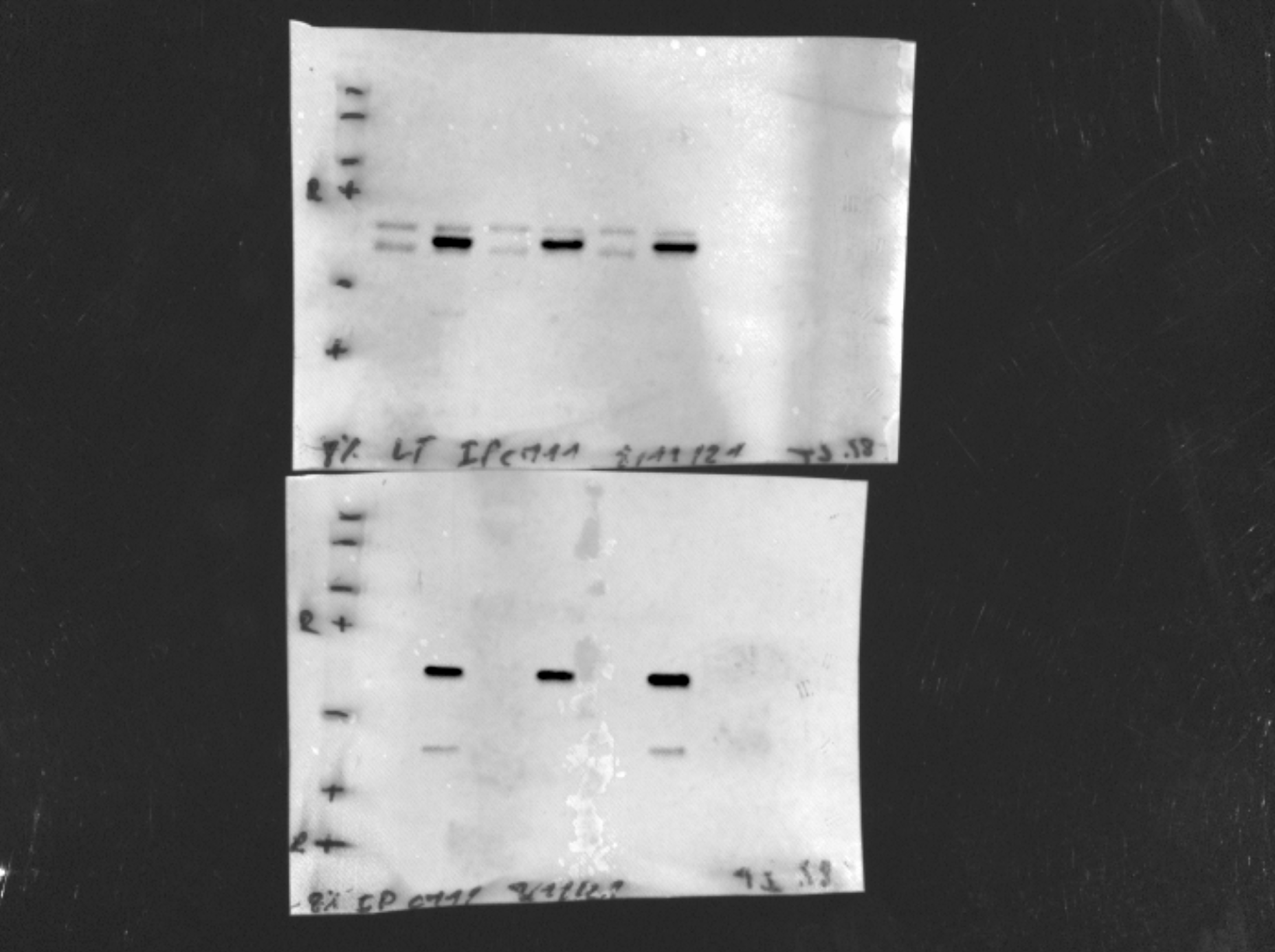

Supplement: Figure 8—source data 3. [file elife-94347-fig8-data3.zip › figure 8A raw data/IGF2BP2/cell extracts/Mazeaud, Clement 2021-11-09 17hr 13min Merge.tif]

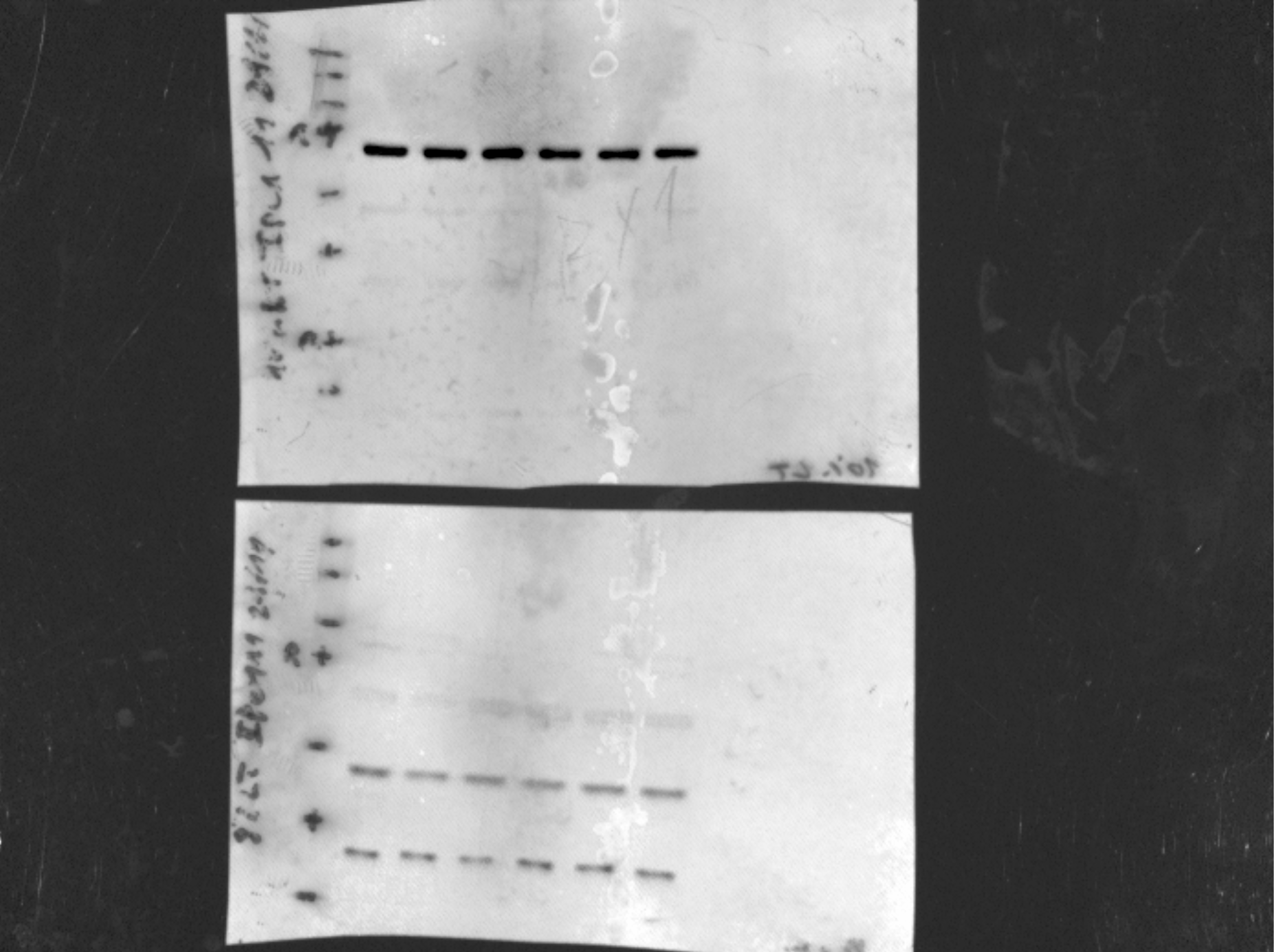

Supplement: Figure 8—source data 3. [file elife-94347-fig8-data3.zip › figure 8A raw data/IGF2BP3/cell extracts/MErge LT.tif]

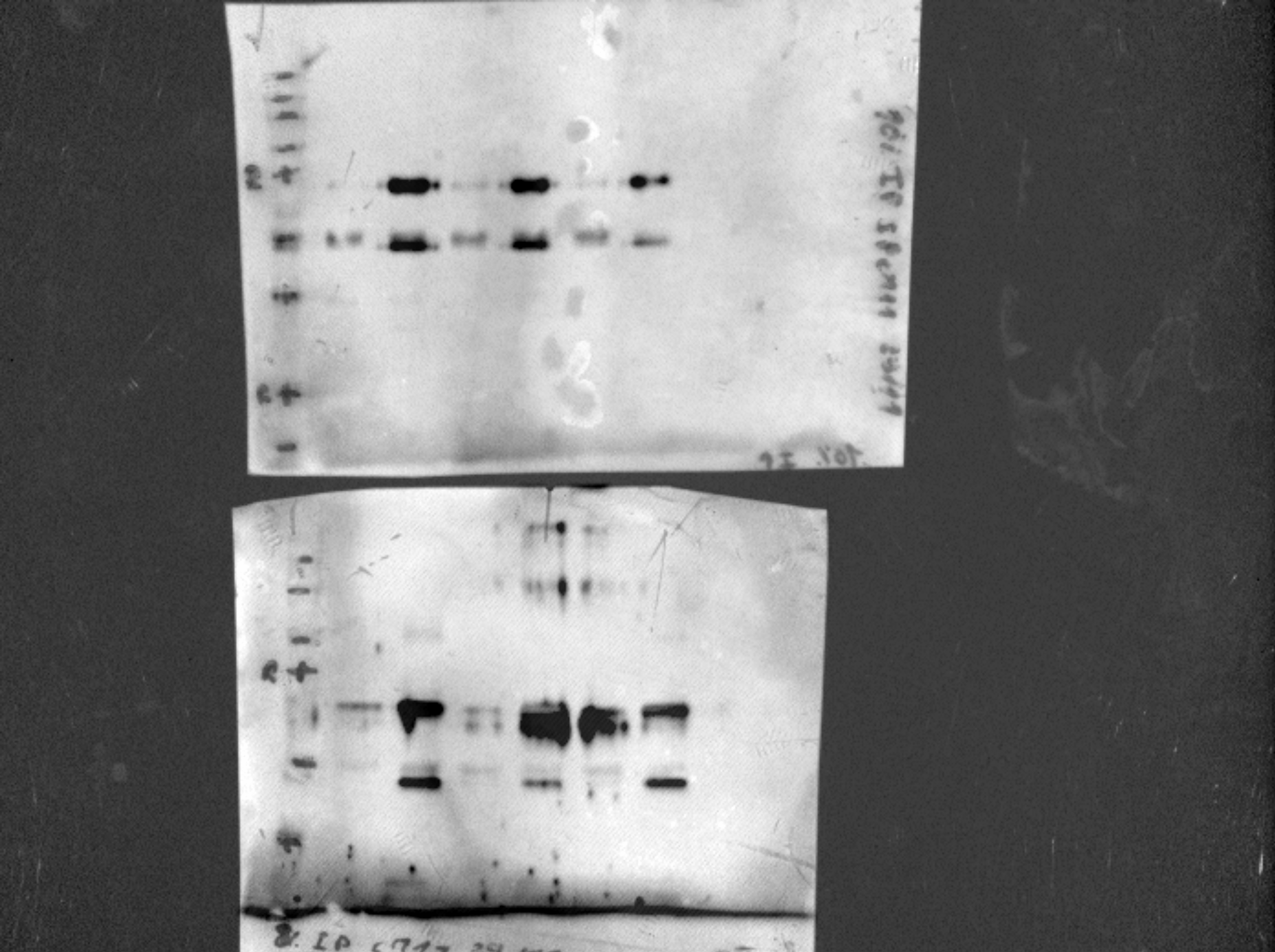

Supplement: Figure 8—source data 3. [file elife-94347-fig8-data3.zip › figure 8A raw data/IGF2BP3/IP anti-HA/Mazeaud, Cleement 2021-12-02 17hr 36min Merge.tif]

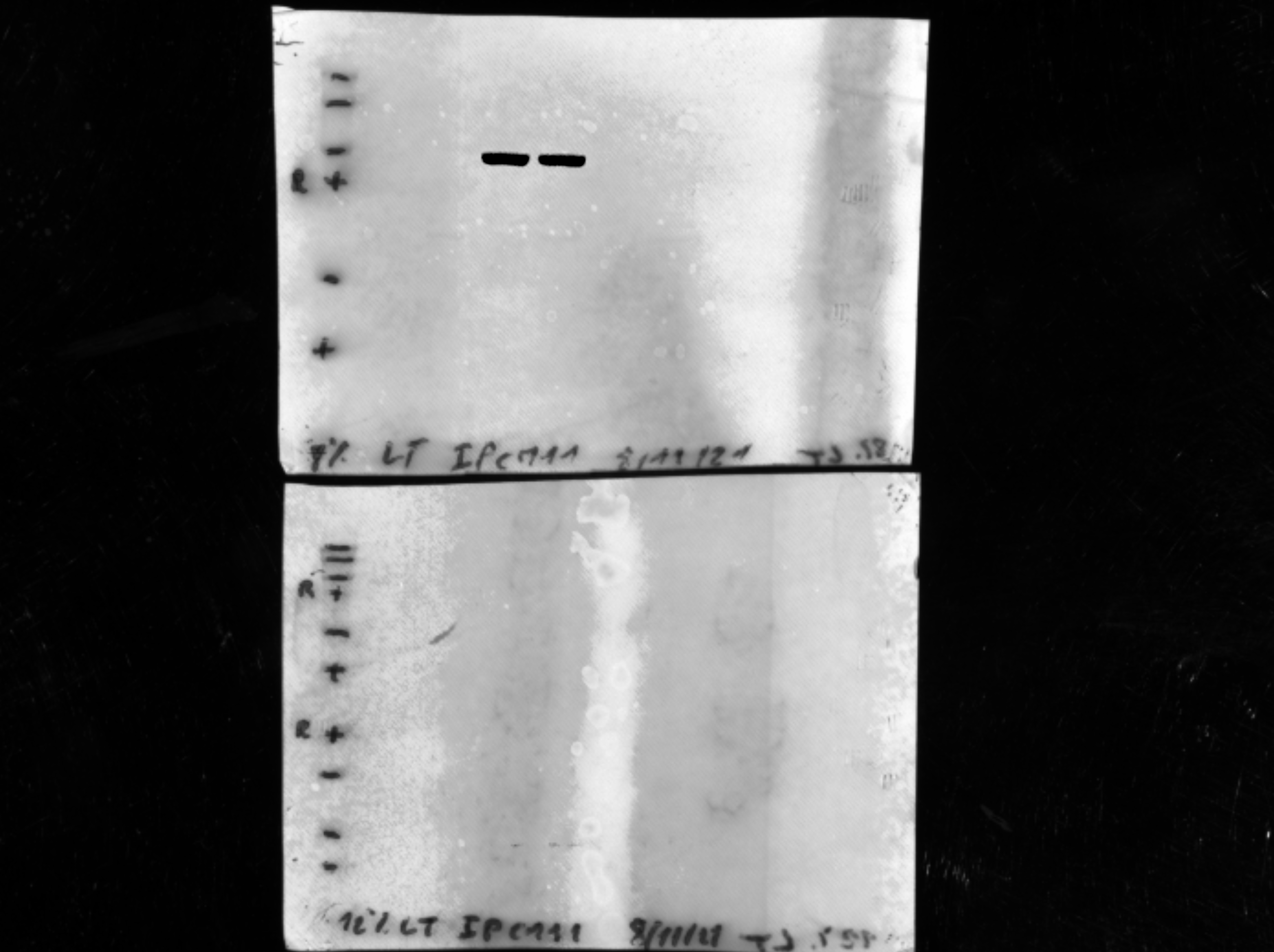

Supplement: Figure 8—source data 3. [file elife-94347-fig8-data3.zip › figure 8A raw data/NS5/cell extracts/merge TL.tif]

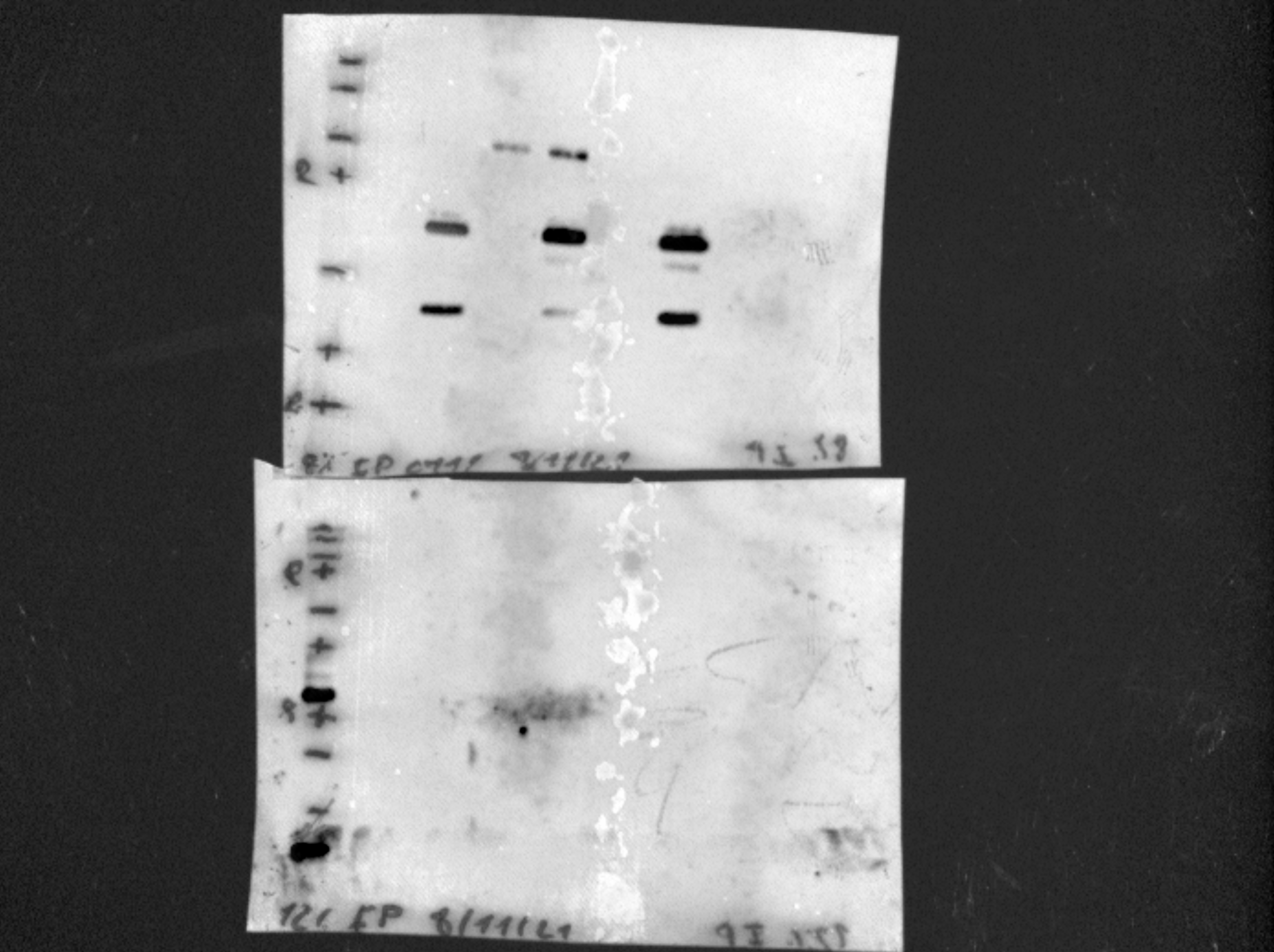

Supplement: Figure 8—source data 3. [file elife-94347-fig8-data3.zip › figure 8A raw data/NS5/IP anti-HA/Merge.tif]

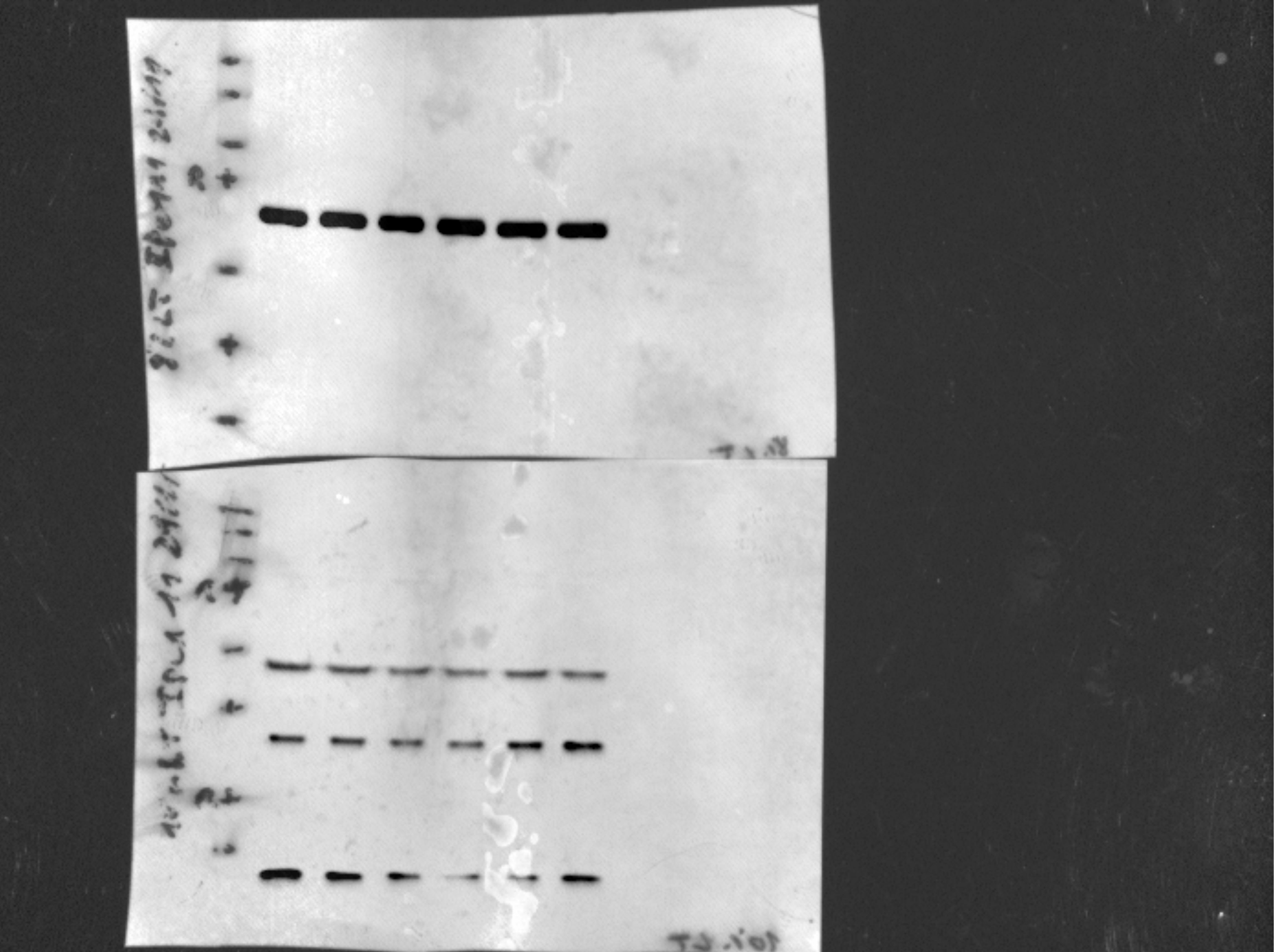

Supplement: Figure 8—source data 3. [file elife-94347-fig8-data3.zip › figure 8A raw data/YBX1/cell extracts/merrge LT.tif]

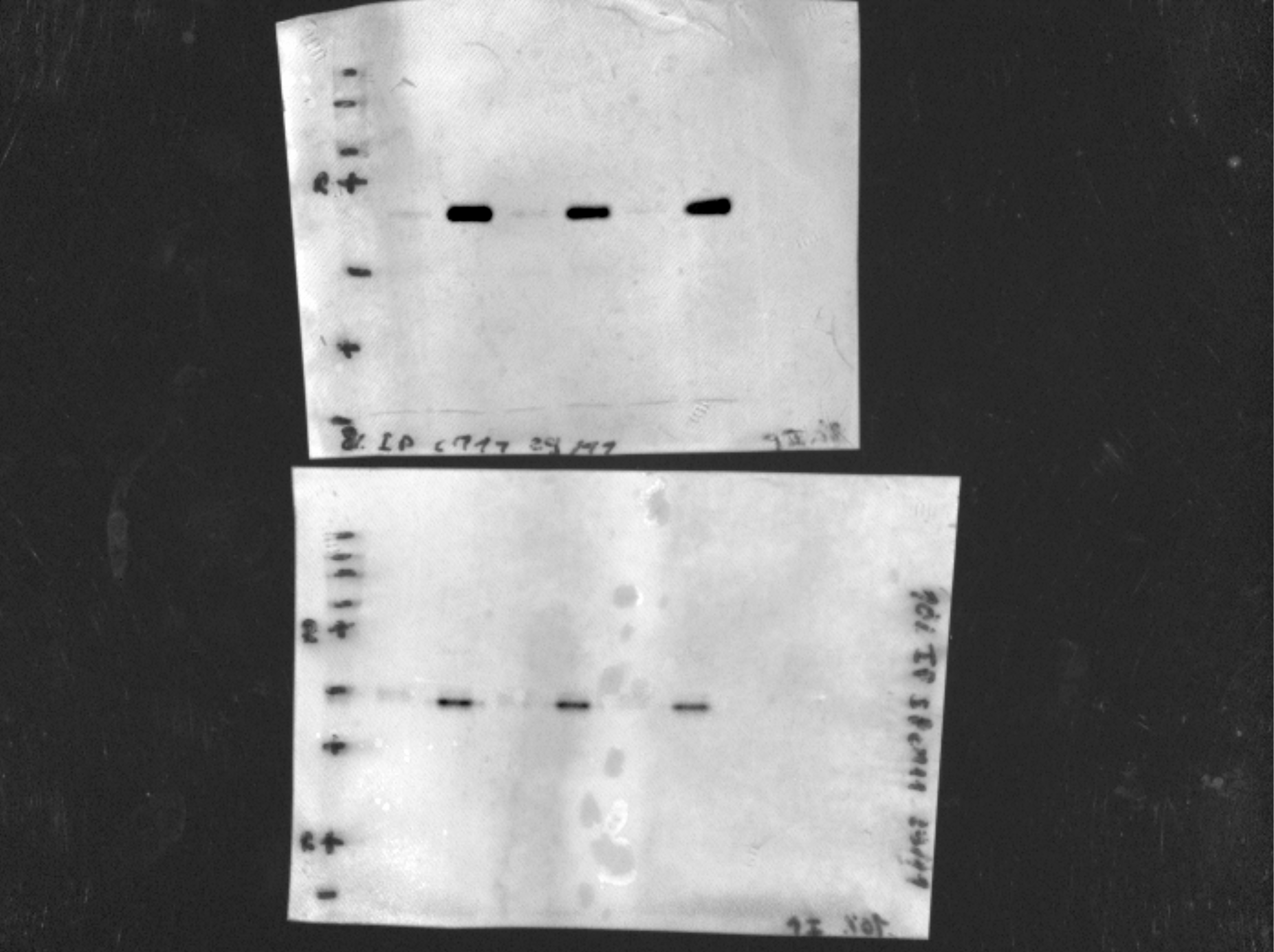

Supplement: Figure 8—source data 3. [file elife-94347-fig8-data3.zip › figure 8A raw data/YBX1/IP anti-HA/merge IP.tif]

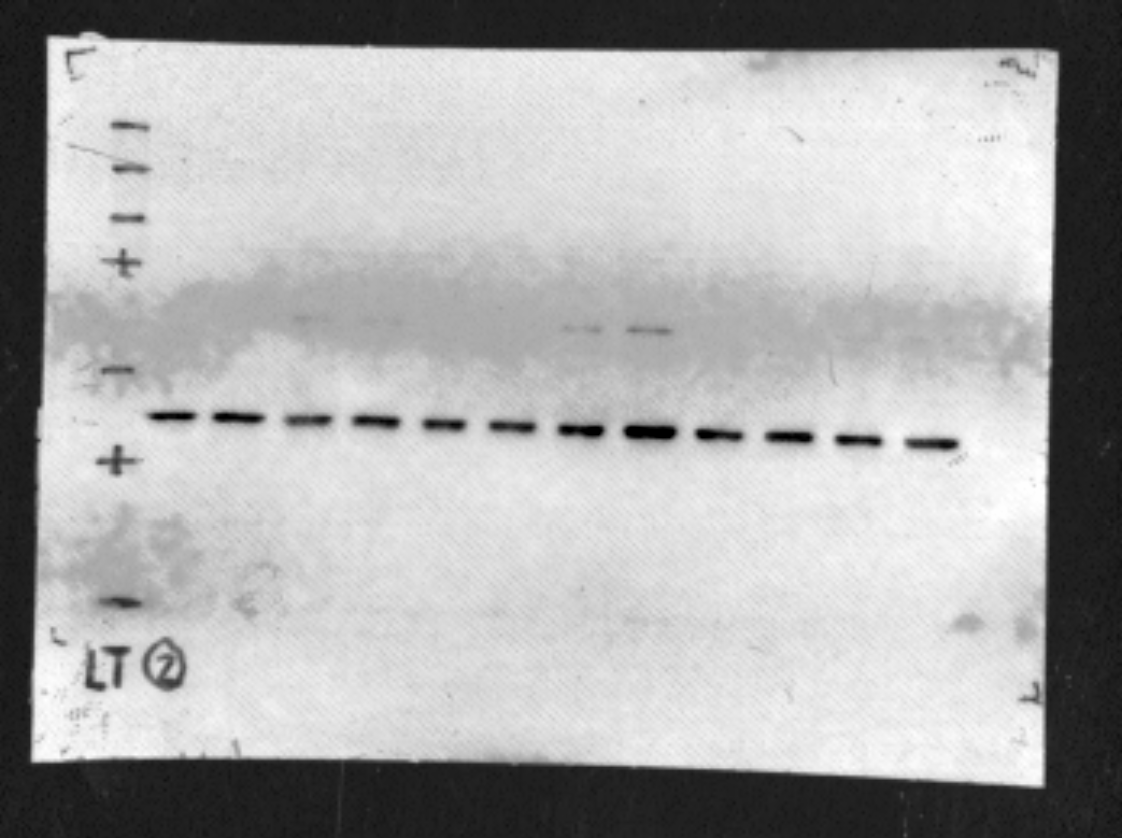

Supplement: Figure 8—figure supplement 3—source data 3. [file elife-94347-fig8-figsupp3-data3.zip › figure 8-figure supplement 3A raw data/Actin/Merge_LT Actine.tif]

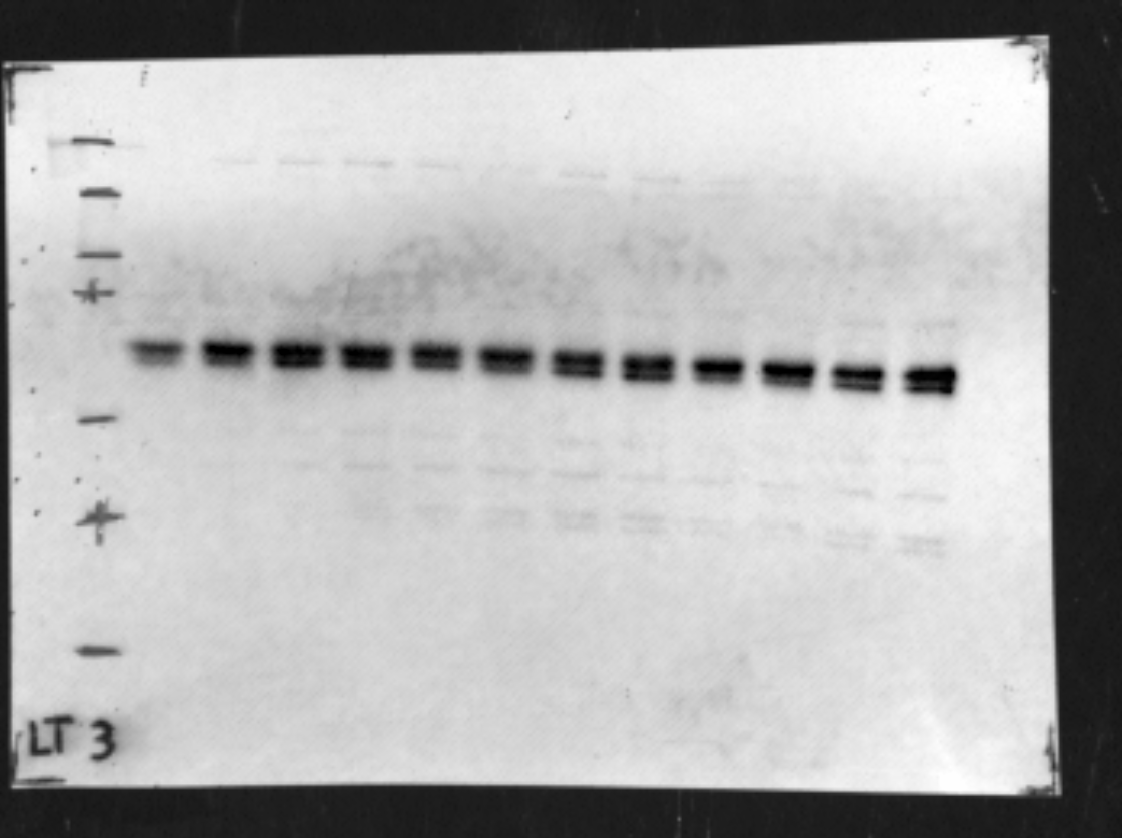

Supplement: Figure 8—figure supplement 3—source data 3. [file elife-94347-fig8-figsupp3-data3.zip › figure 8-figure supplement 3A raw data/ATL2/cell extracts/Merge_LT ATL2.tif]

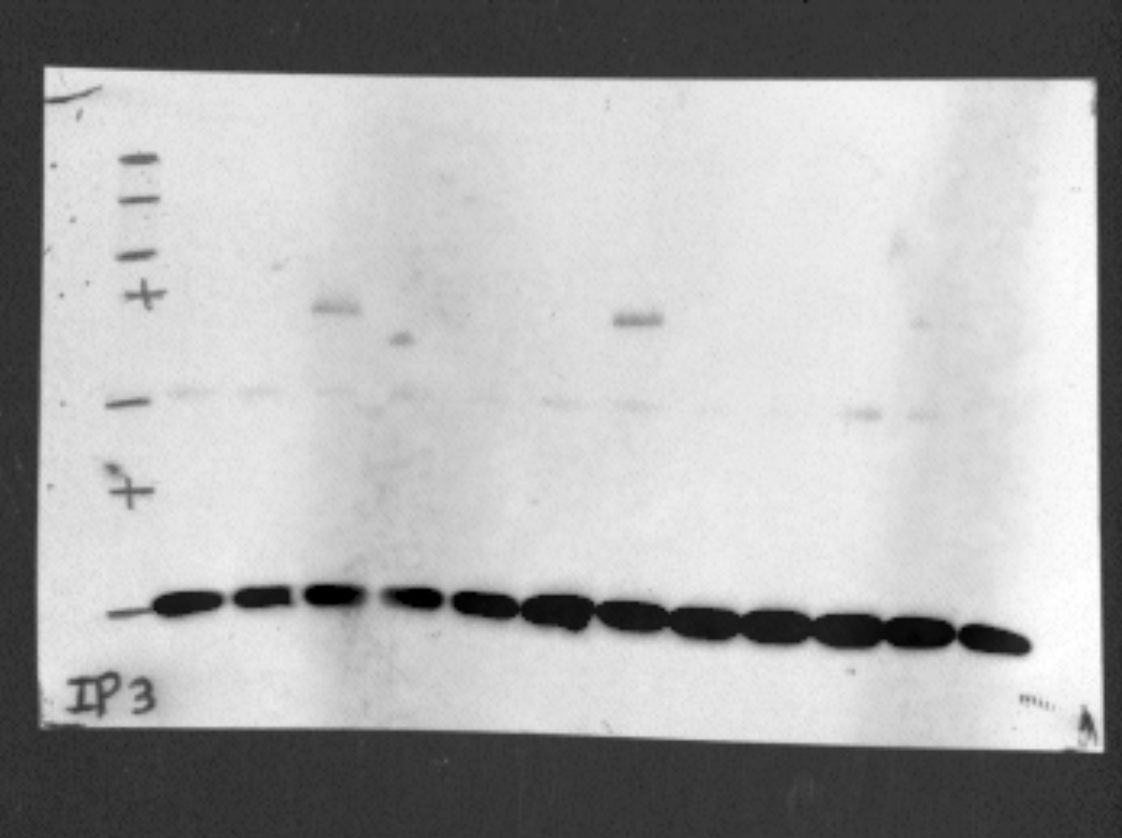

Supplement: Figure 8—figure supplement 3—source data 3. [file elife-94347-fig8-figsupp3-data3.zip › figure 8-figure supplement 3A raw data/ATL2/IP anti-HA/Merge_IP ATL2.tif]

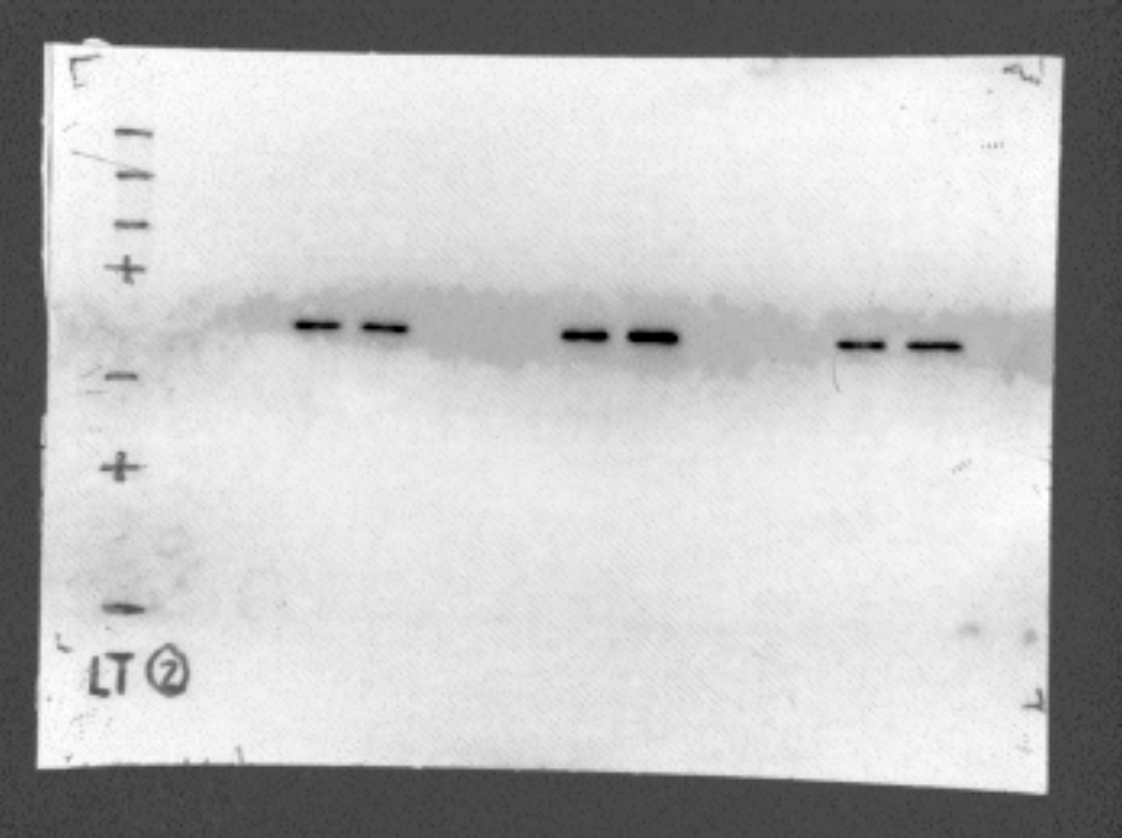

Supplement: Figure 8—figure supplement 3—source data 3. [file elife-94347-fig8-figsupp3-data3.zip › figure 8-figure supplement 3A raw data/HA/cell extracts/Merge_LT HA.tif]

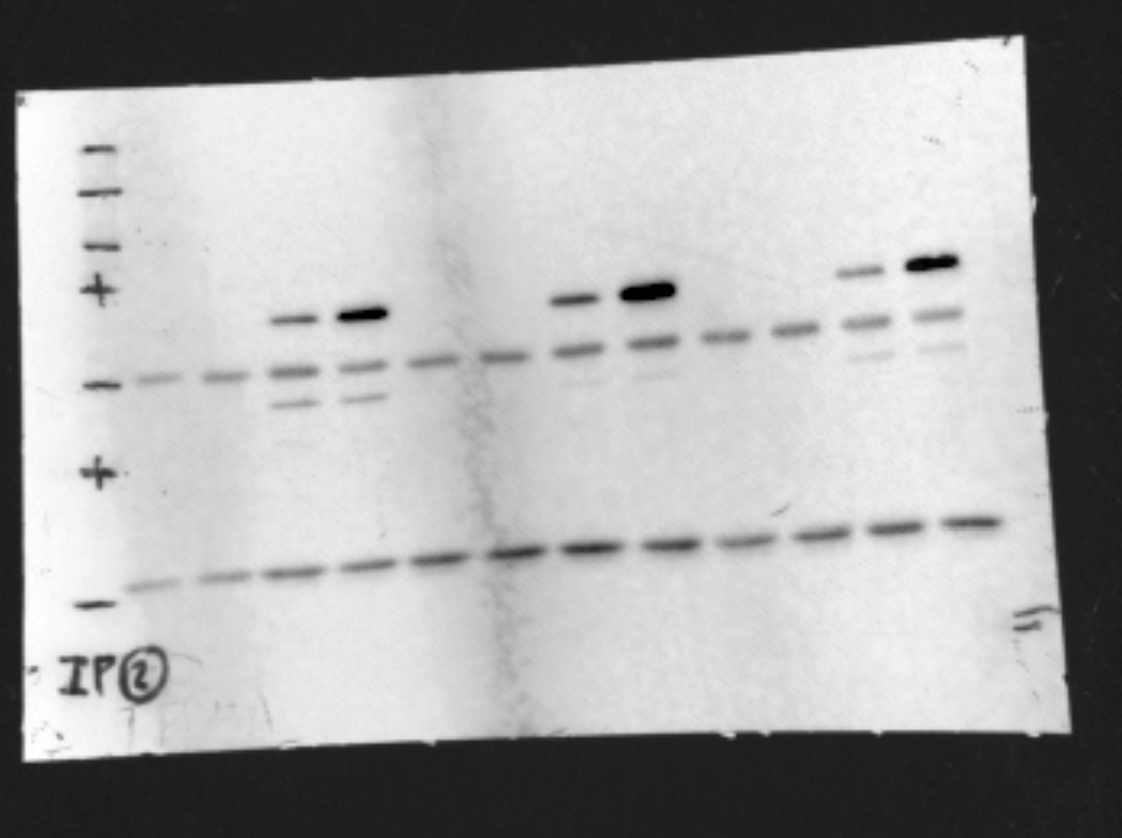

Supplement: Figure 8—figure supplement 3—source data 3. [file elife-94347-fig8-figsupp3-data3.zip › figure 8-figure supplement 3A raw data/HA/IP anti-HA/Merge_IP HA.tif]

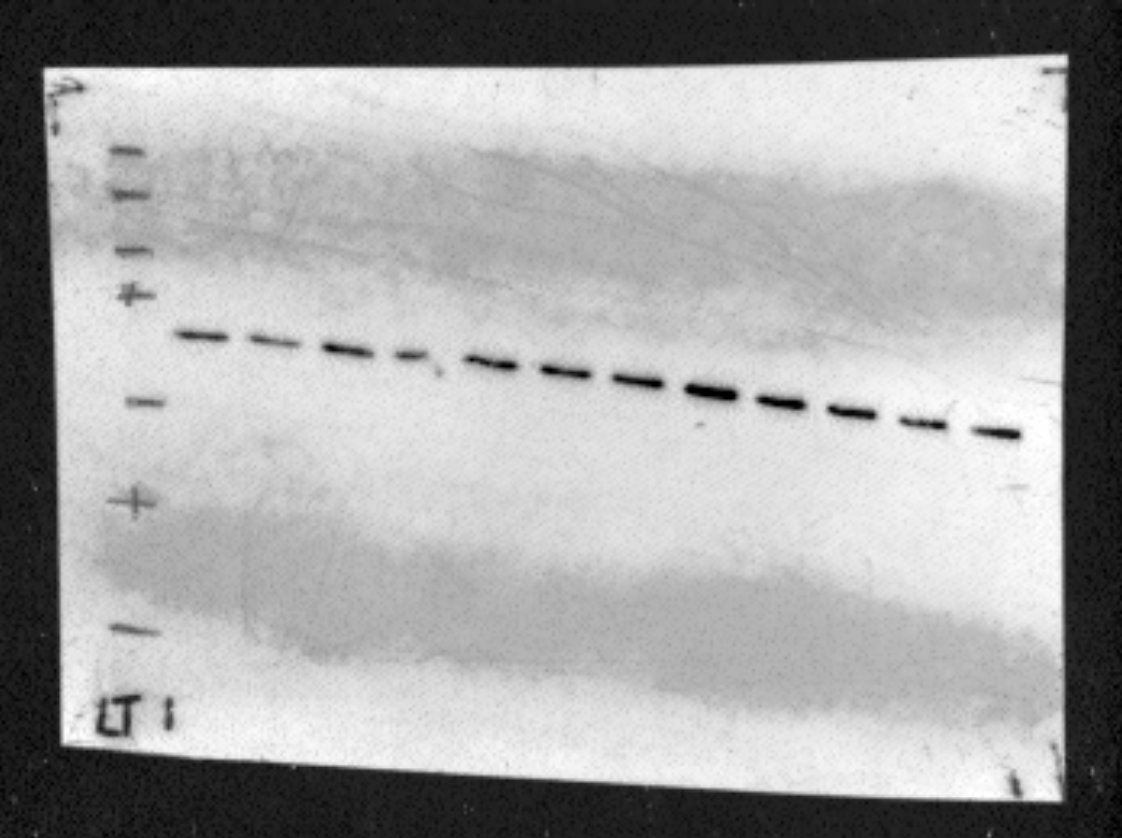

Supplement: Figure 8—figure supplement 3—source data 3. [file elife-94347-fig8-figsupp3-data3.zip › figure 8-figure supplement 3A raw data/IGF2BP1/cell extracts/Merge_LT IMP1.tif]

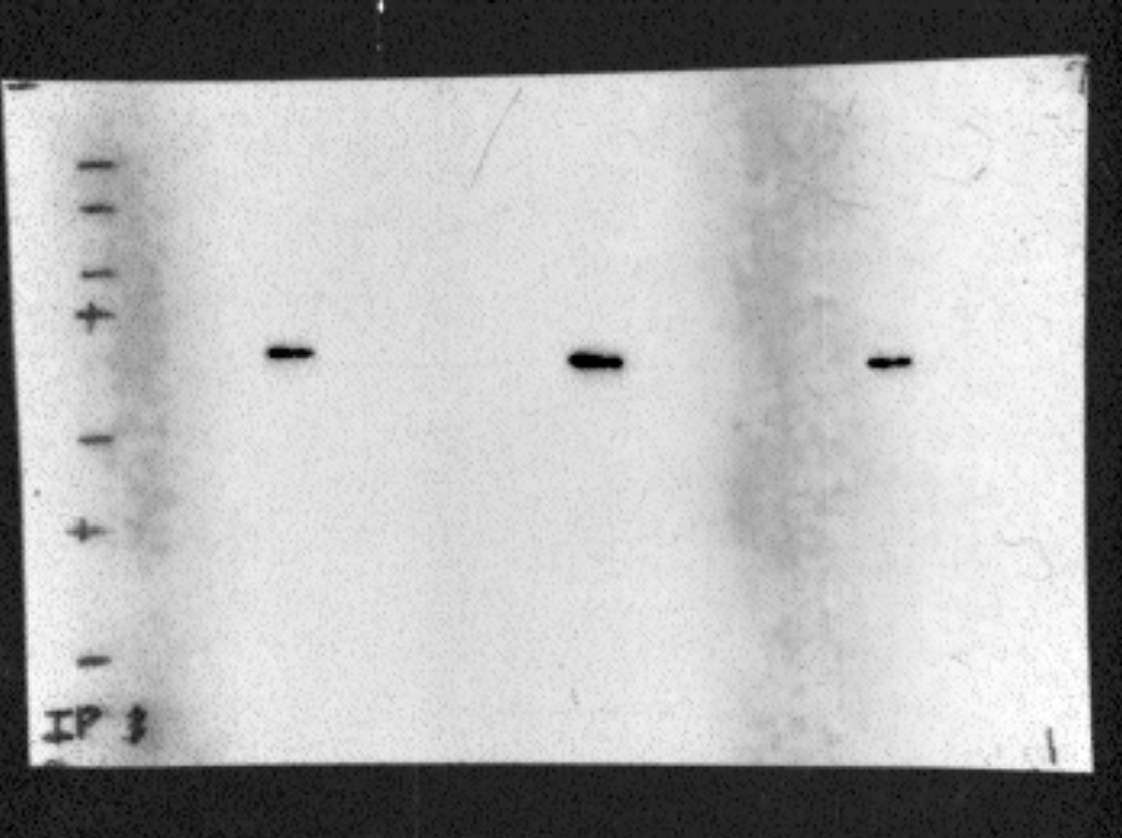

Supplement: Figure 8—figure supplement 3—source data 3. [file elife-94347-fig8-figsupp3-data3.zip › figure 8-figure supplement 3A raw data/IGF2BP1/IP anti-HA/Merge_IP IMP1.tif]

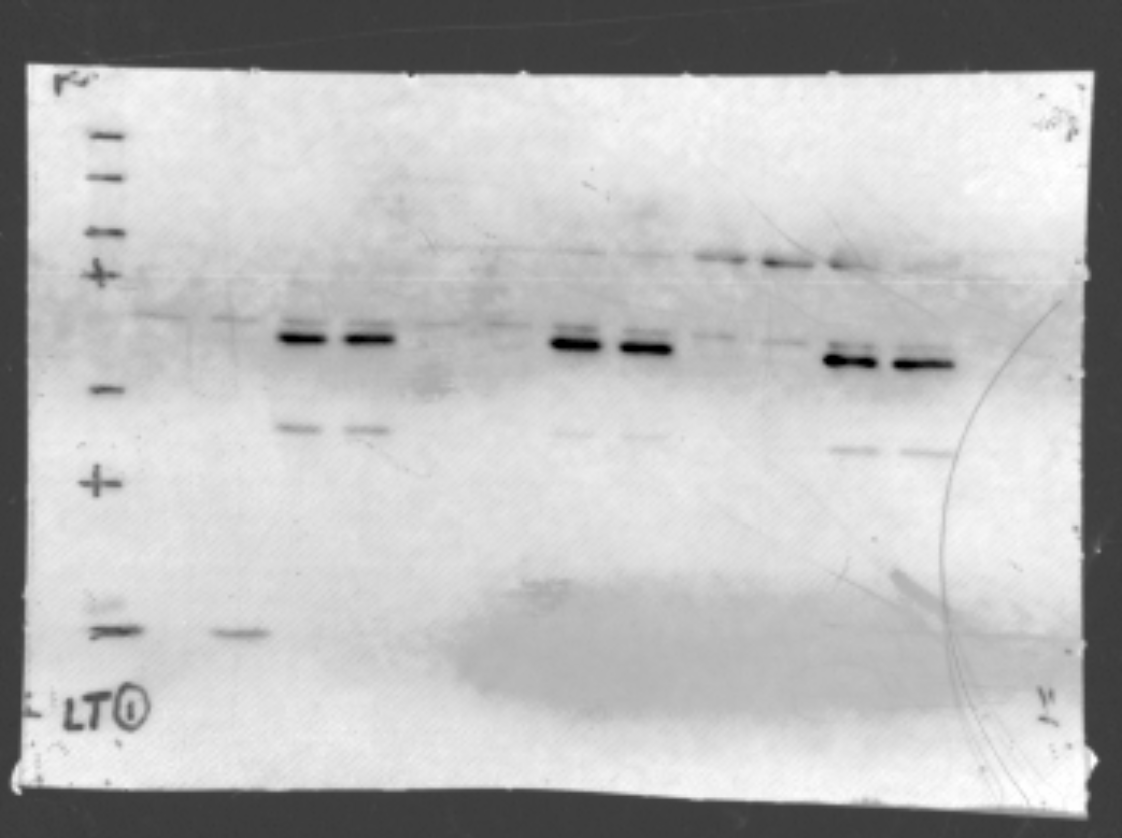

Supplement: Figure 8—figure supplement 3—source data 3. [file elife-94347-fig8-figsupp3-data3.zip › figure 8-figure supplement 3A raw data/IGF2BP2/cell extracts/Merge_LT IMP2.tif]

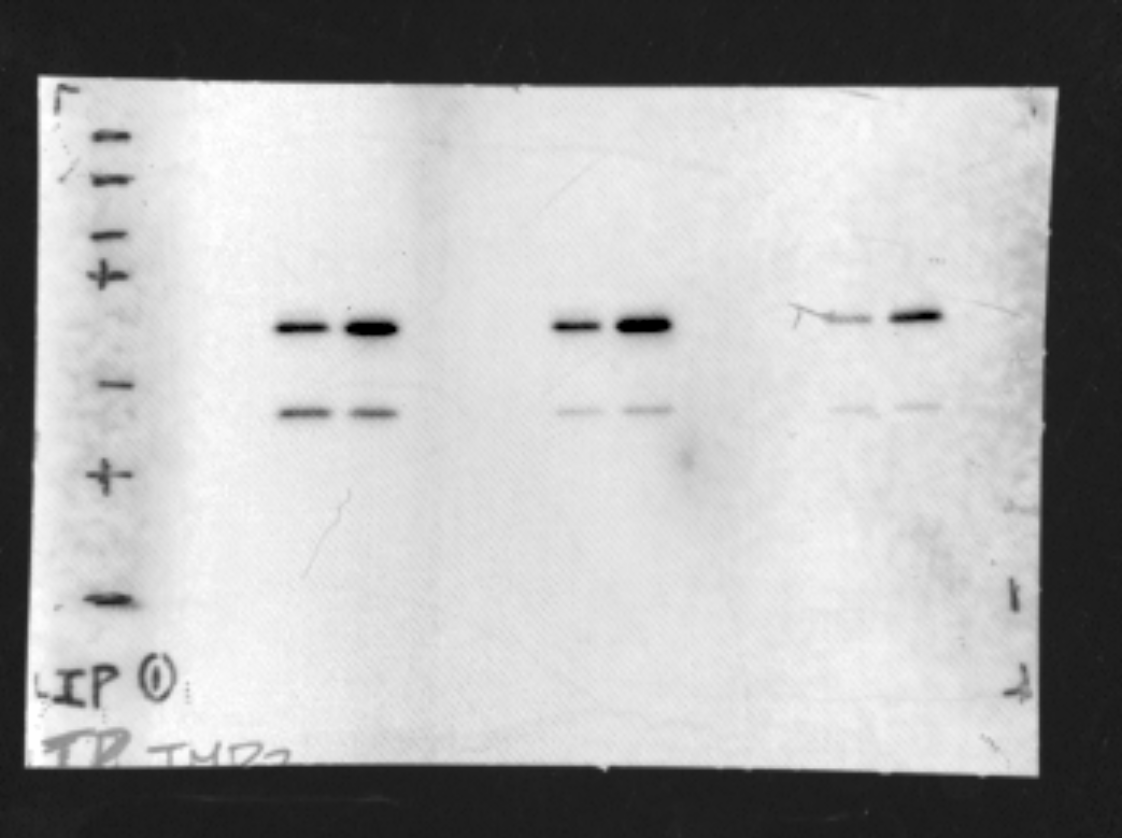

Supplement: Figure 8—figure supplement 3—source data 3. [file elife-94347-fig8-figsupp3-data3.zip › figure 8-figure supplement 3A raw data/IGF2BP2/IP anti-HA/Merge_IP IMP2.tif]

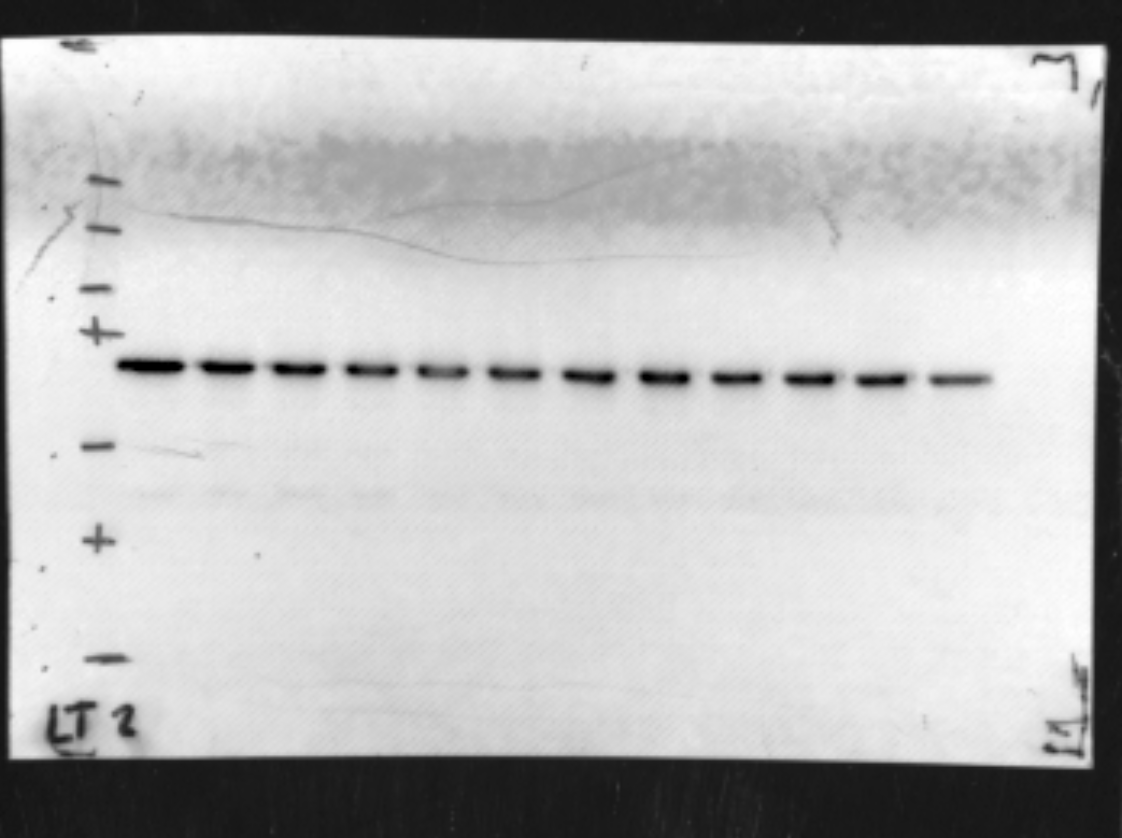

Supplement: Figure 8—figure supplement 3—source data 3. [file elife-94347-fig8-figsupp3-data3.zip › figure 8-figure supplement 3A raw data/IGF2BP3/cell extracts/Merge_LT IMP3.tif]

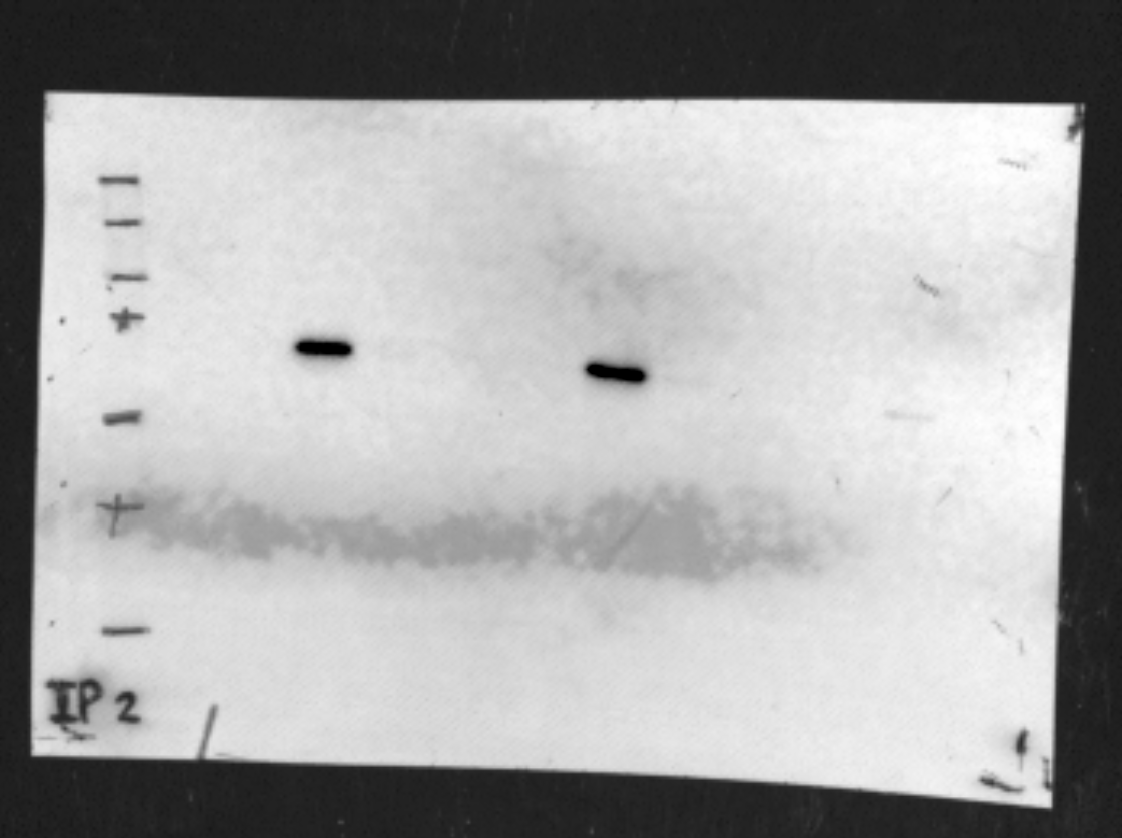

Supplement: Figure 8—figure supplement 3—source data 3. [file elife-94347-fig8-figsupp3-data3.zip › figure 8-figure supplement 3A raw data/IGF2BP3/IP anti-HA/Merge_IP IMP3.tif]

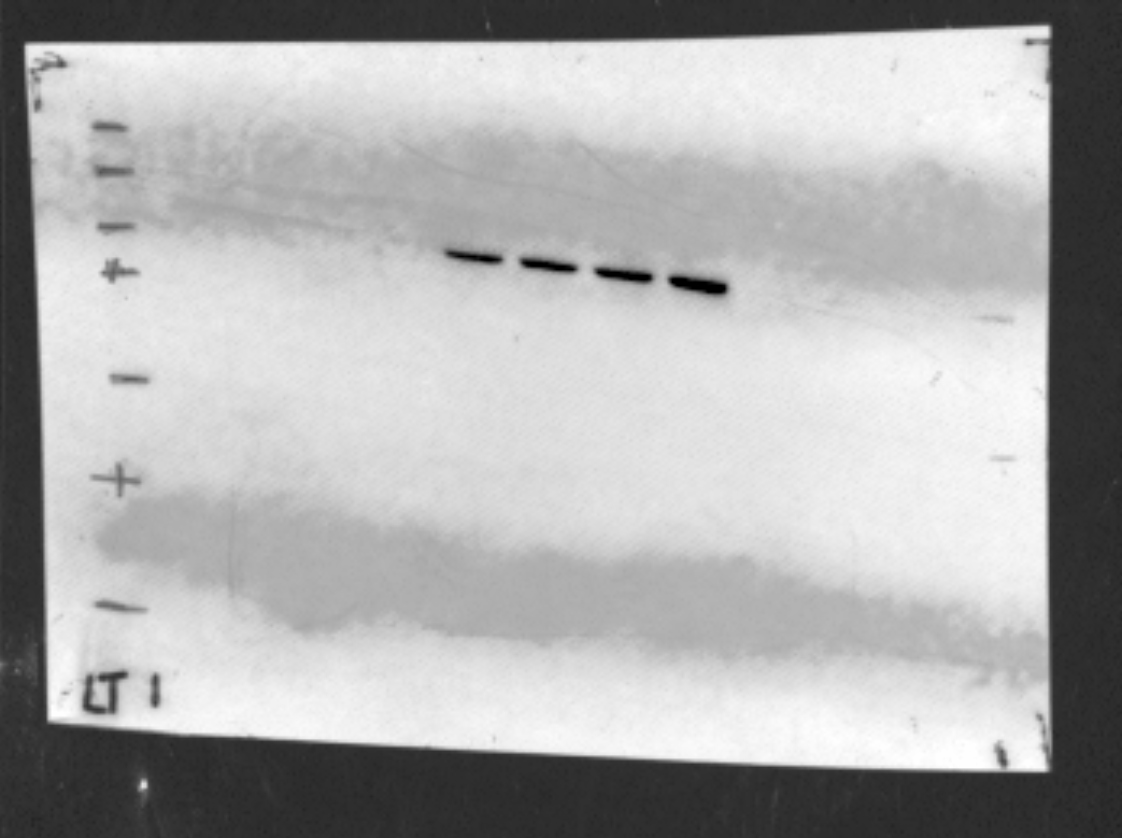

Supplement: Figure 8—figure supplement 3—source data 3. [file elife-94347-fig8-figsupp3-data3.zip › figure 8-figure supplement 3A raw data/NS5/cell extracts/Merge_LT NS5 ZV.tif]

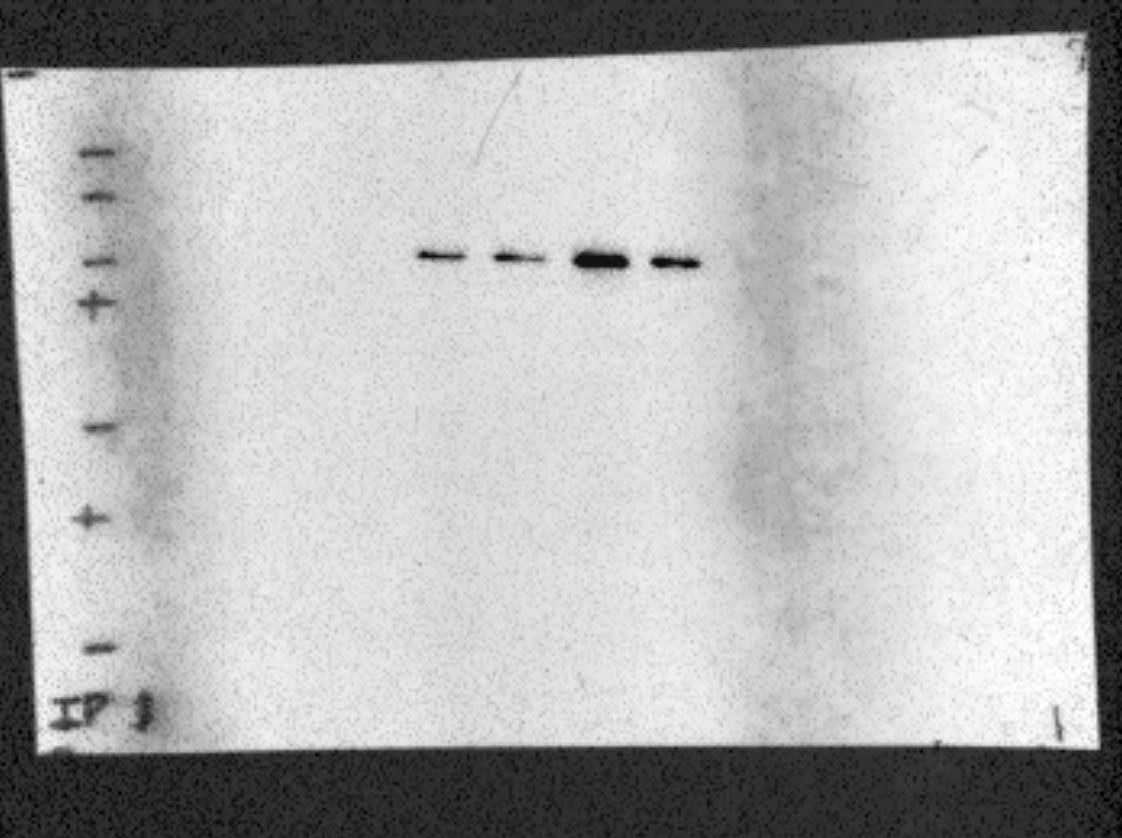

Supplement: Figure 8—figure supplement 3—source data 3. [file elife-94347-fig8-figsupp3-data3.zip › figure 8-figure supplement 3A raw data/NS5/IP anti-HA/Merge_IP NS5 ZV.tif]

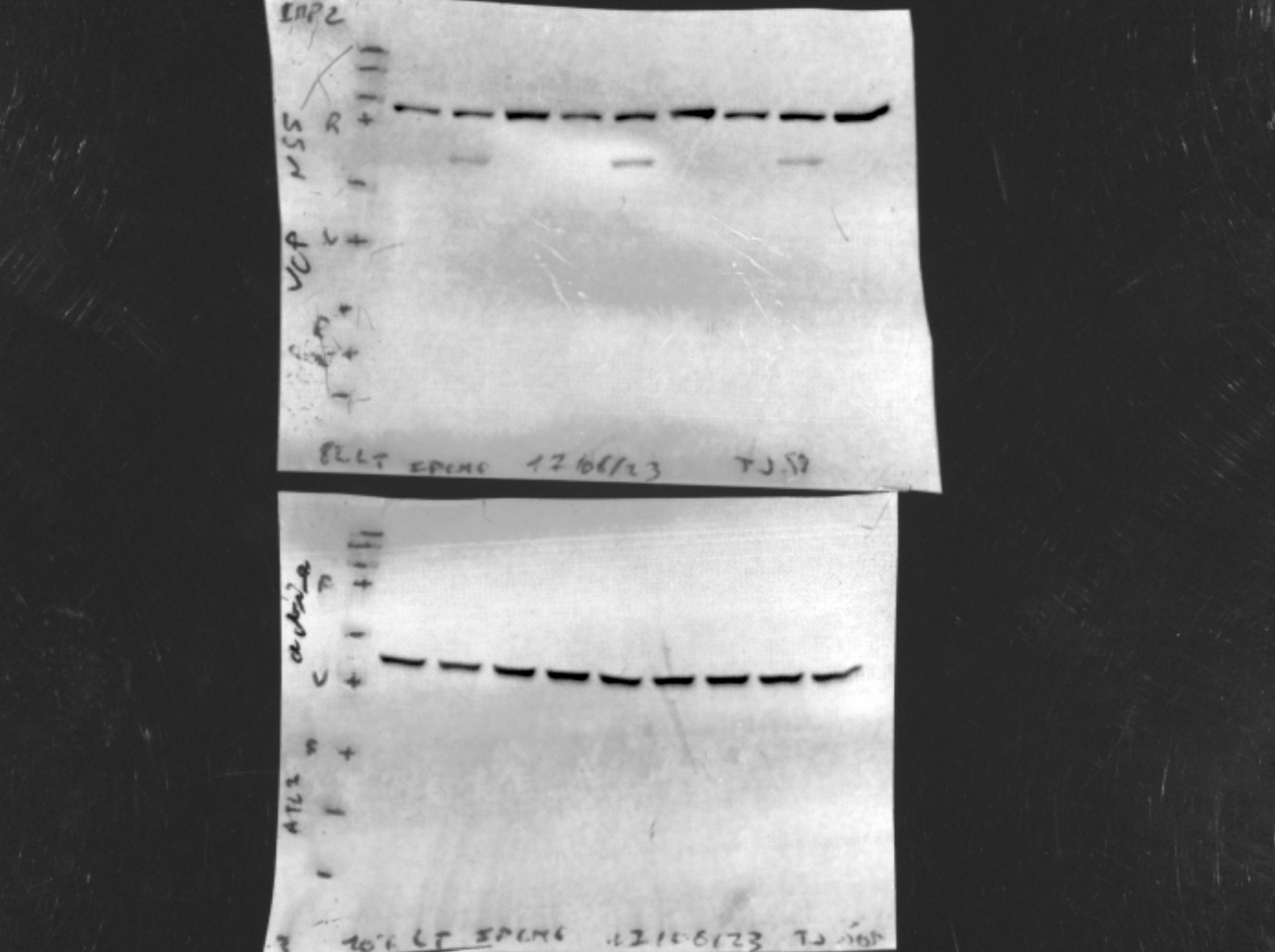

Supplement: Figure 9—figure supplement 2—source data 3. [file elife-94347-fig9-figsupp2-data3.zip › figure 9-figure supplement 2A raw data/Actin/Melany Juarez 2023-06-21 16hr 45min Merge.tif]

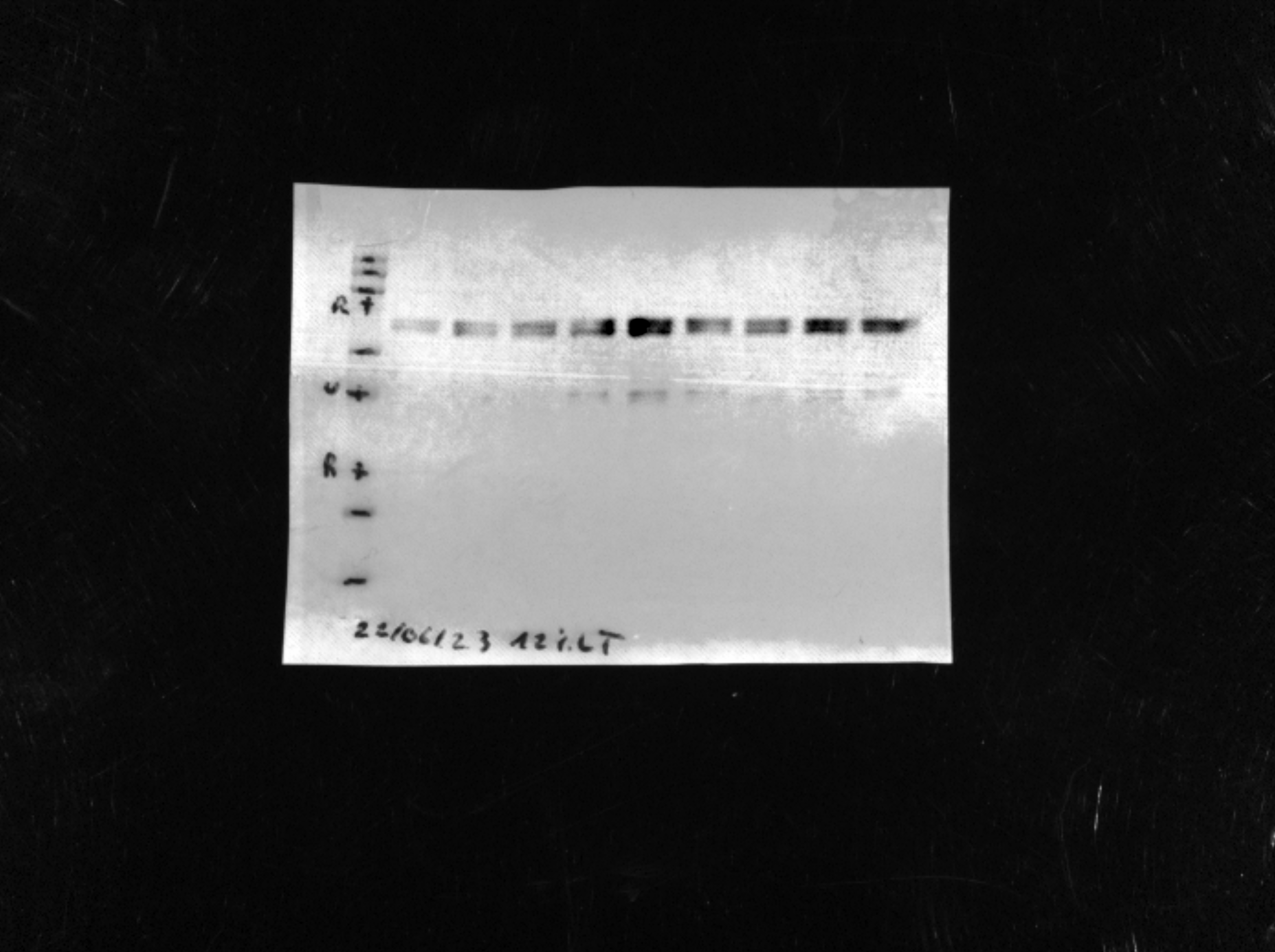

Supplement: Figure 9—figure supplement 2—source data 3. [file elife-94347-fig9-figsupp2-data3.zip › figure 9-figure supplement 2A raw data/ATL2/cell extracts/merge LT.tif]

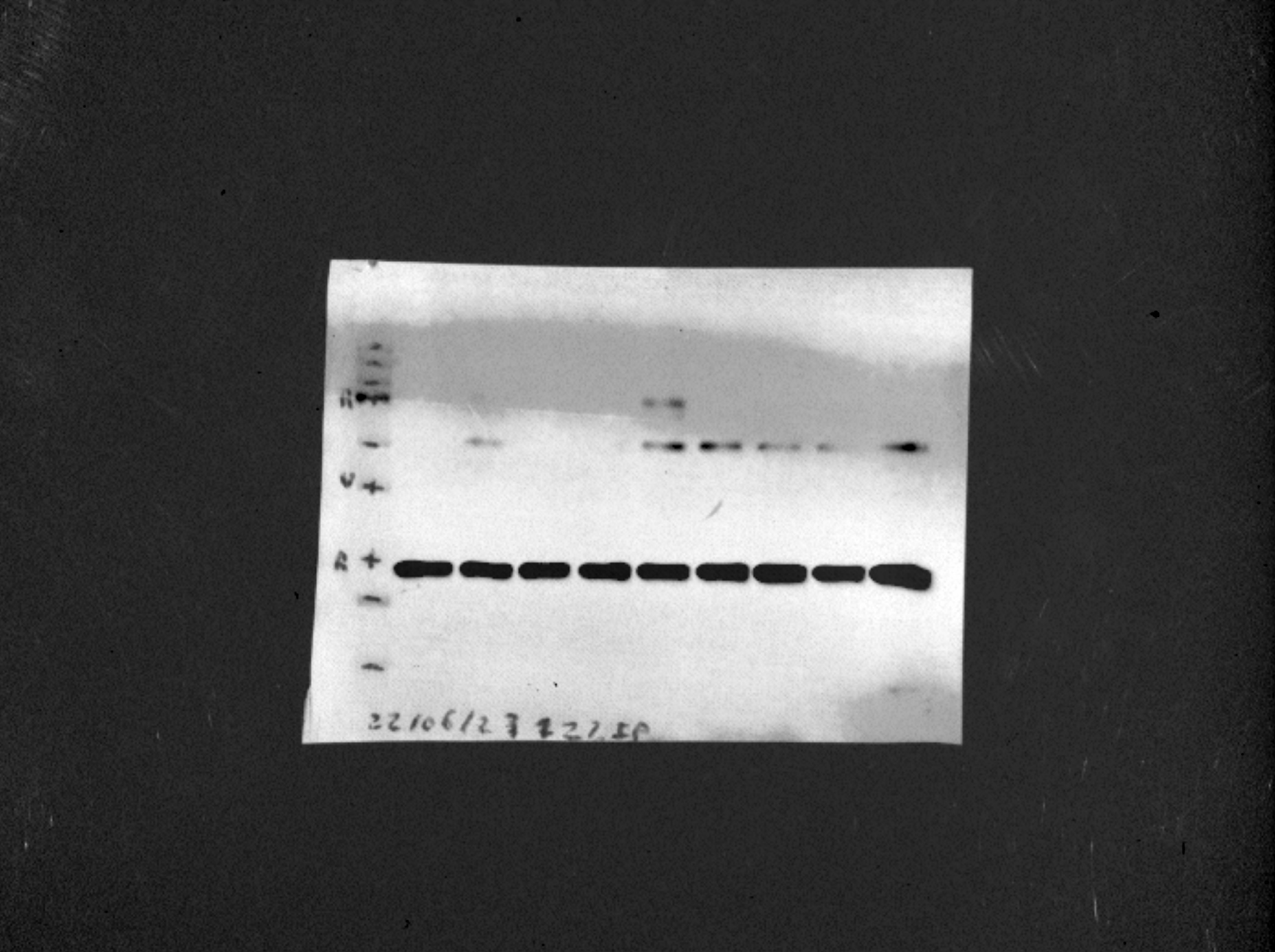

Supplement: Figure 9—figure supplement 2—source data 3. [file elife-94347-fig9-figsupp2-data3.zip › figure 9-figure supplement 2A raw data/ATL2/IP anti-HA/Melany Juarez 2023-06-24 17hr 38min Merge.tif]

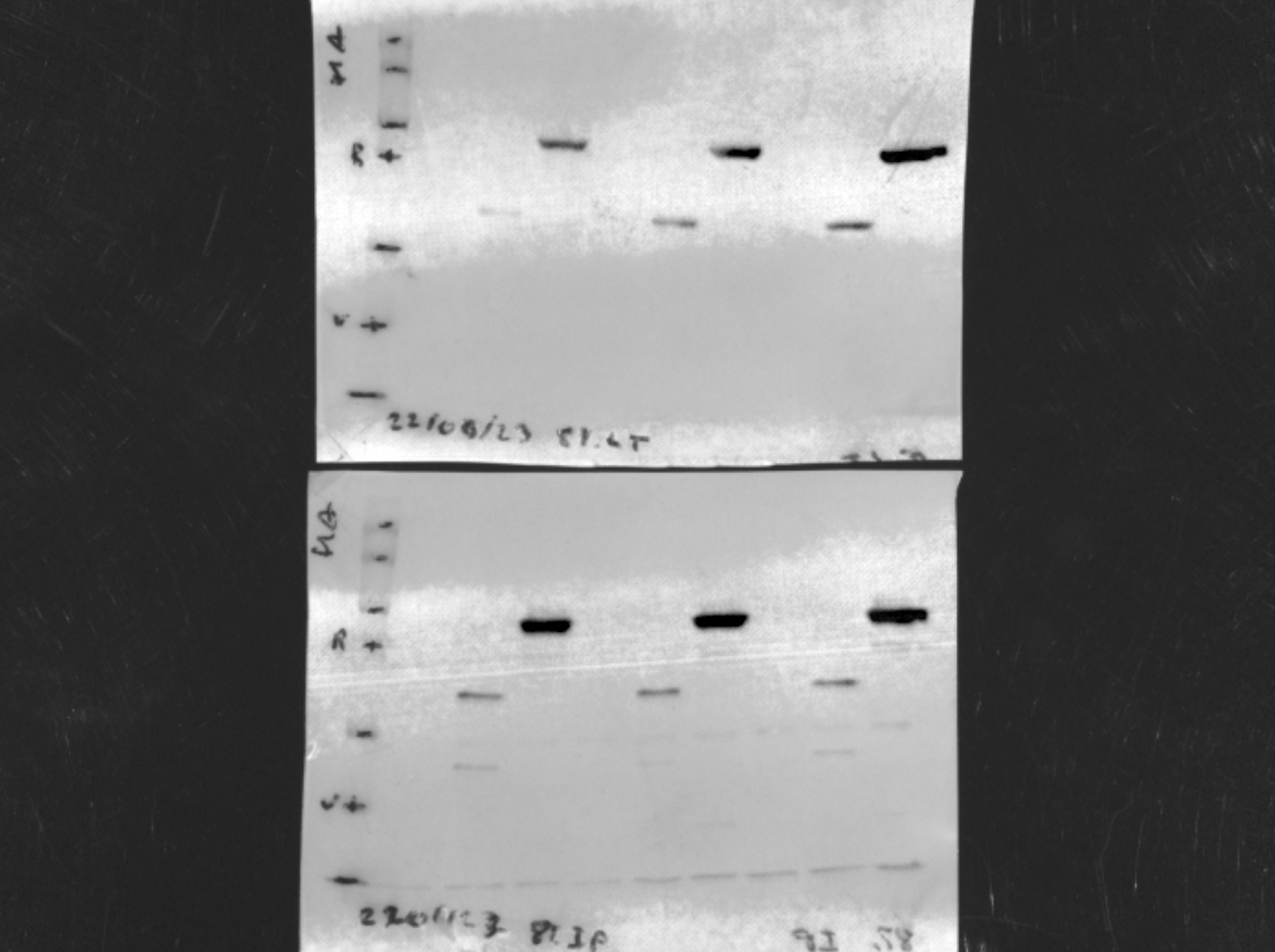

Supplement: Figure 9—figure supplement 2—source data 3. [file elife-94347-fig9-figsupp2-data3.zip › figure 9-figure supplement 2A raw data/HA/cell extracts/Melany Juarez 2023-06-24 16hr 59min Merge.tif]

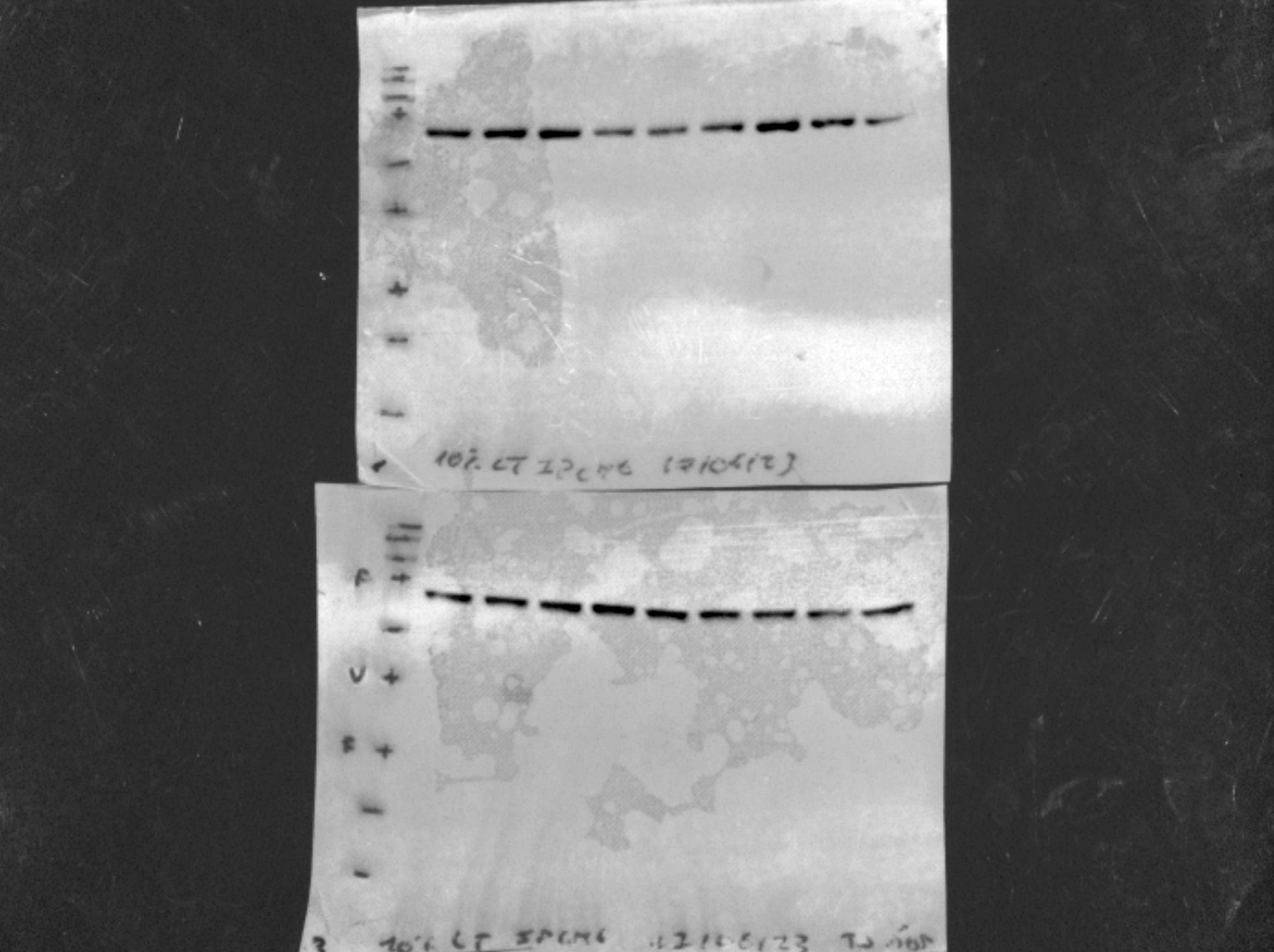

Supplement: Figure 9—figure supplement 2—source data 3. [file elife-94347-fig9-figsupp2-data3.zip › figure 9-figure supplement 2A raw data/IGF2BP1/cell extracts/Melany Juarez 2023-06-19 14hr 05min mERGE.tif]

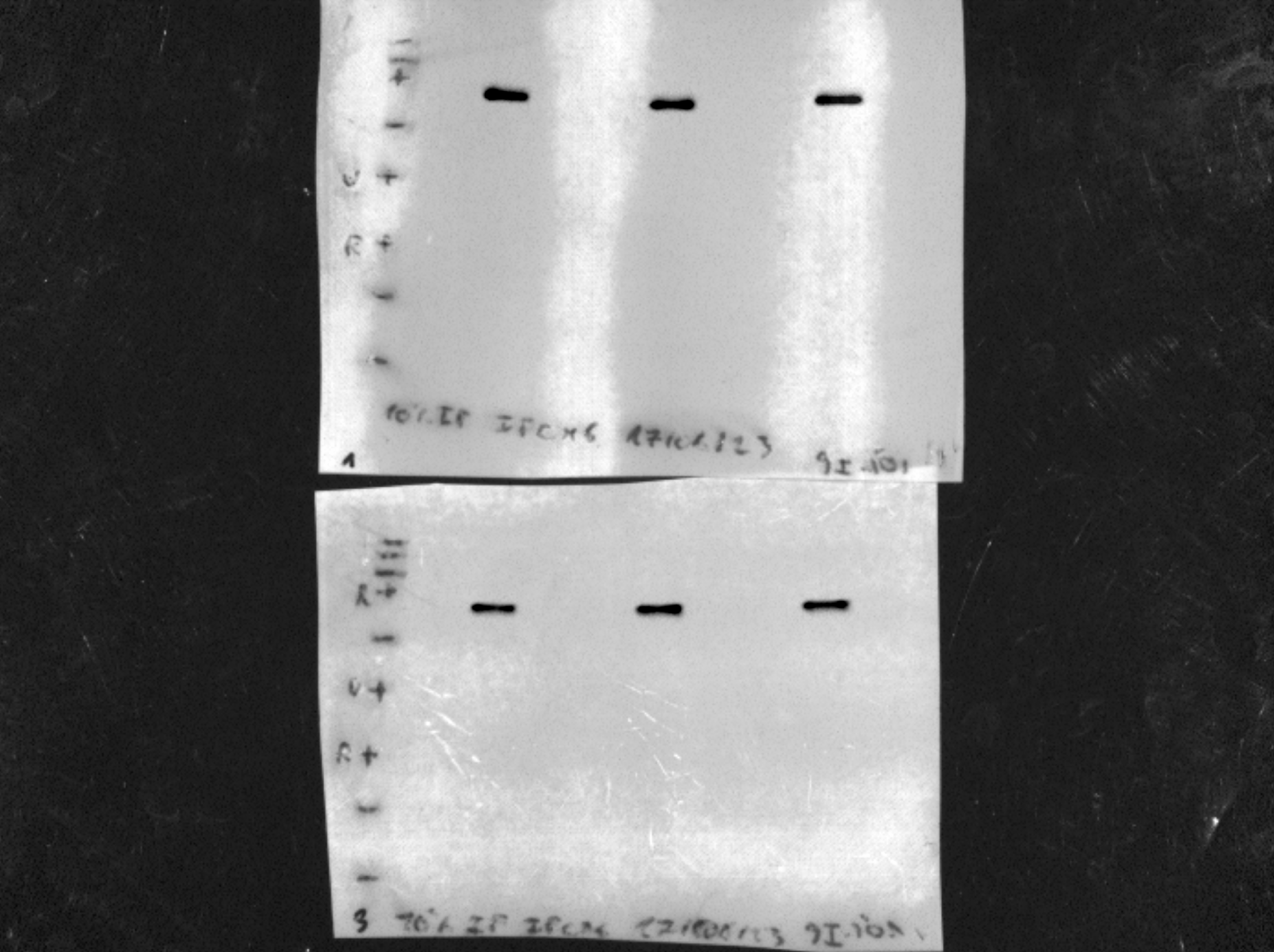

Supplement: Figure 9—figure supplement 2—source data 3. [file elife-94347-fig9-figsupp2-data3.zip › figure 9-figure supplement 2A raw data/IGF2BP1/IP anti-HA/Melany Juarez 2023-06-19 14hr 13min Merge.tif]

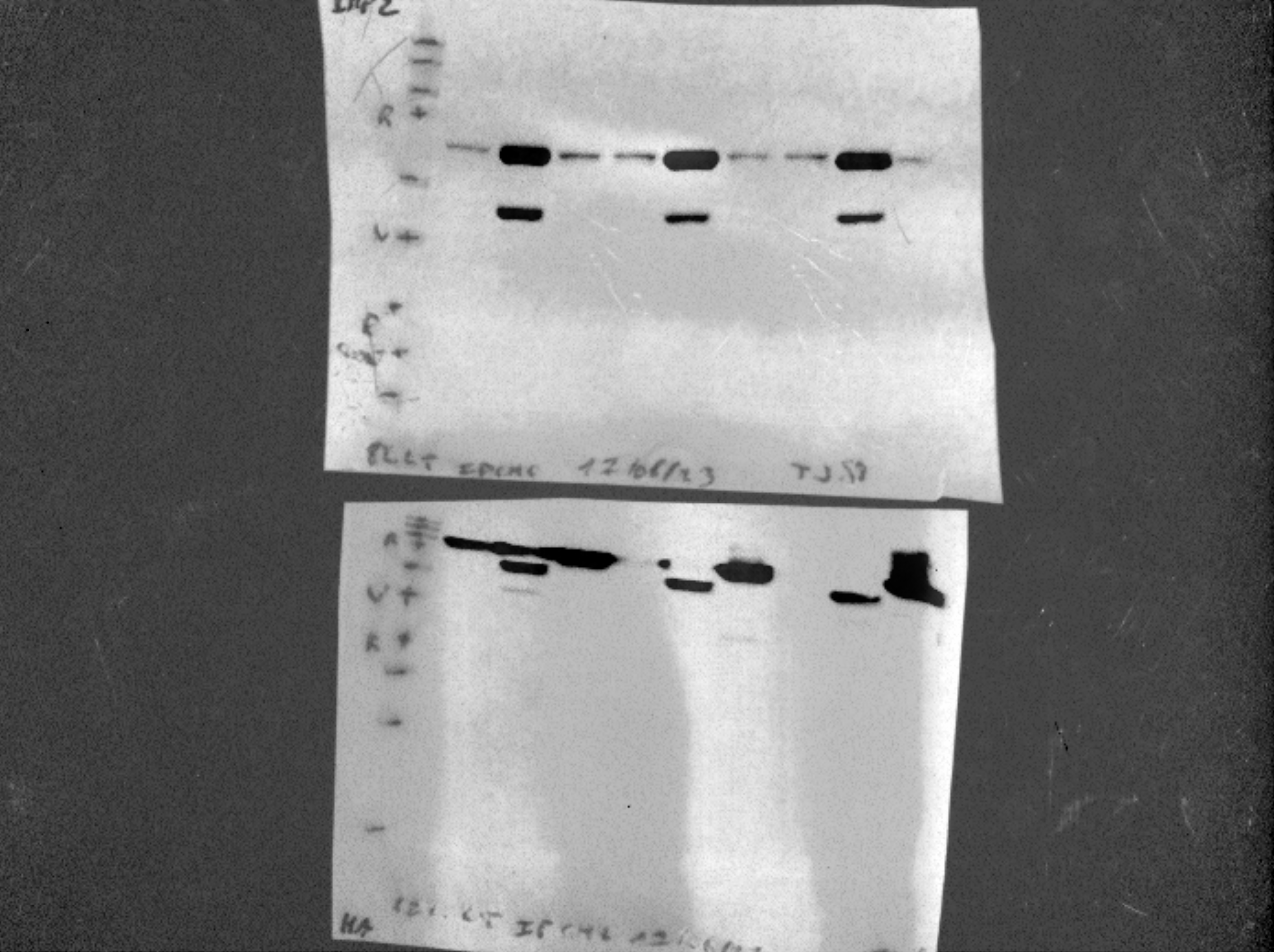

Supplement: Figure 9—figure supplement 2—source data 3. [file elife-94347-fig9-figsupp2-data3.zip › figure 9-figure supplement 2A raw data/IGF2BP2/cell extracts/MERGE LT.tif]

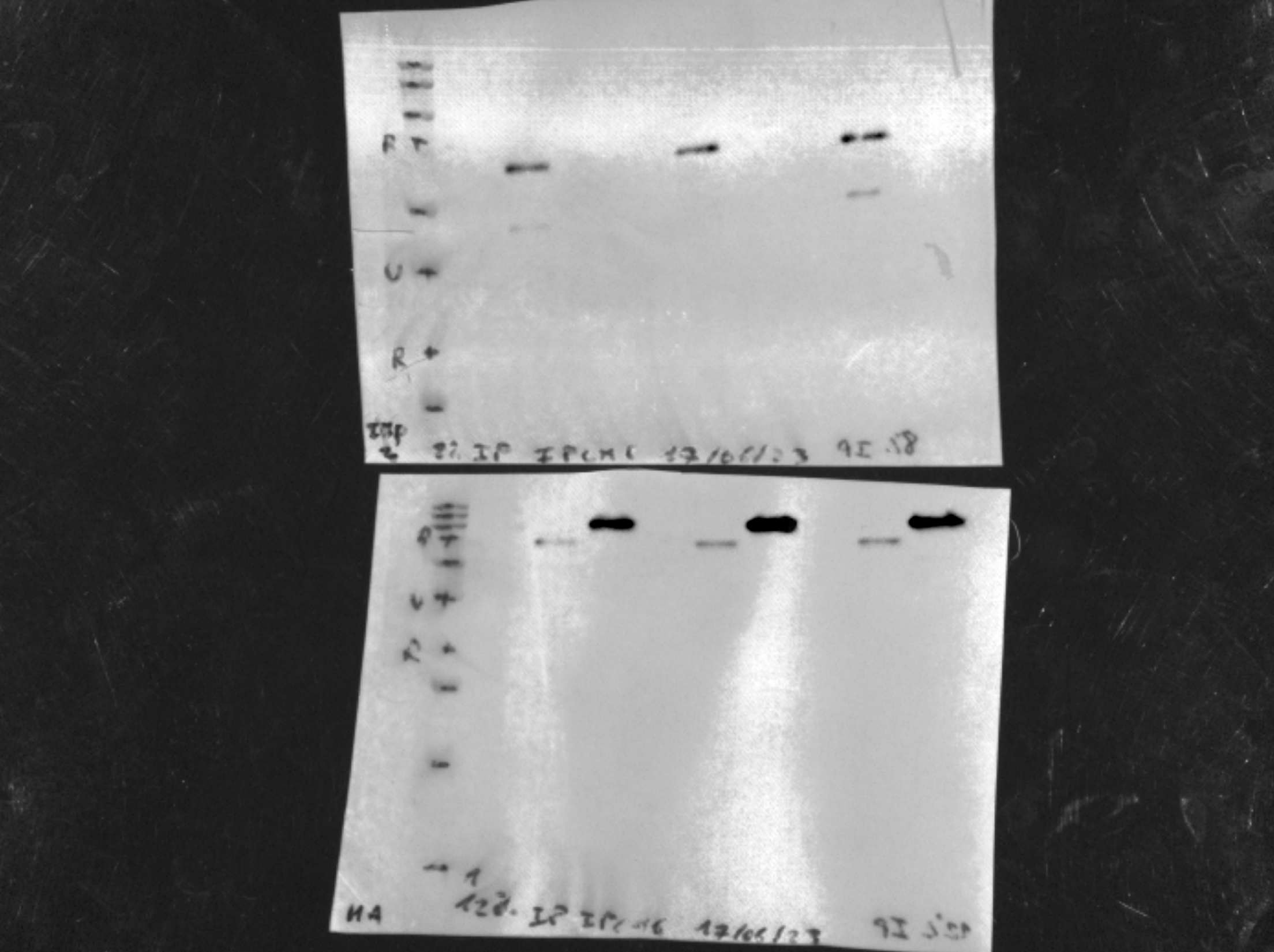

Supplement: Figure 9—figure supplement 2—source data 3. [file elife-94347-fig9-figsupp2-data3.zip › figure 9-figure supplement 2A raw data/IGF2BP2/IP anti-HA/Melany Juarez 2023-06-19 13hr 50min MERGE.tif]

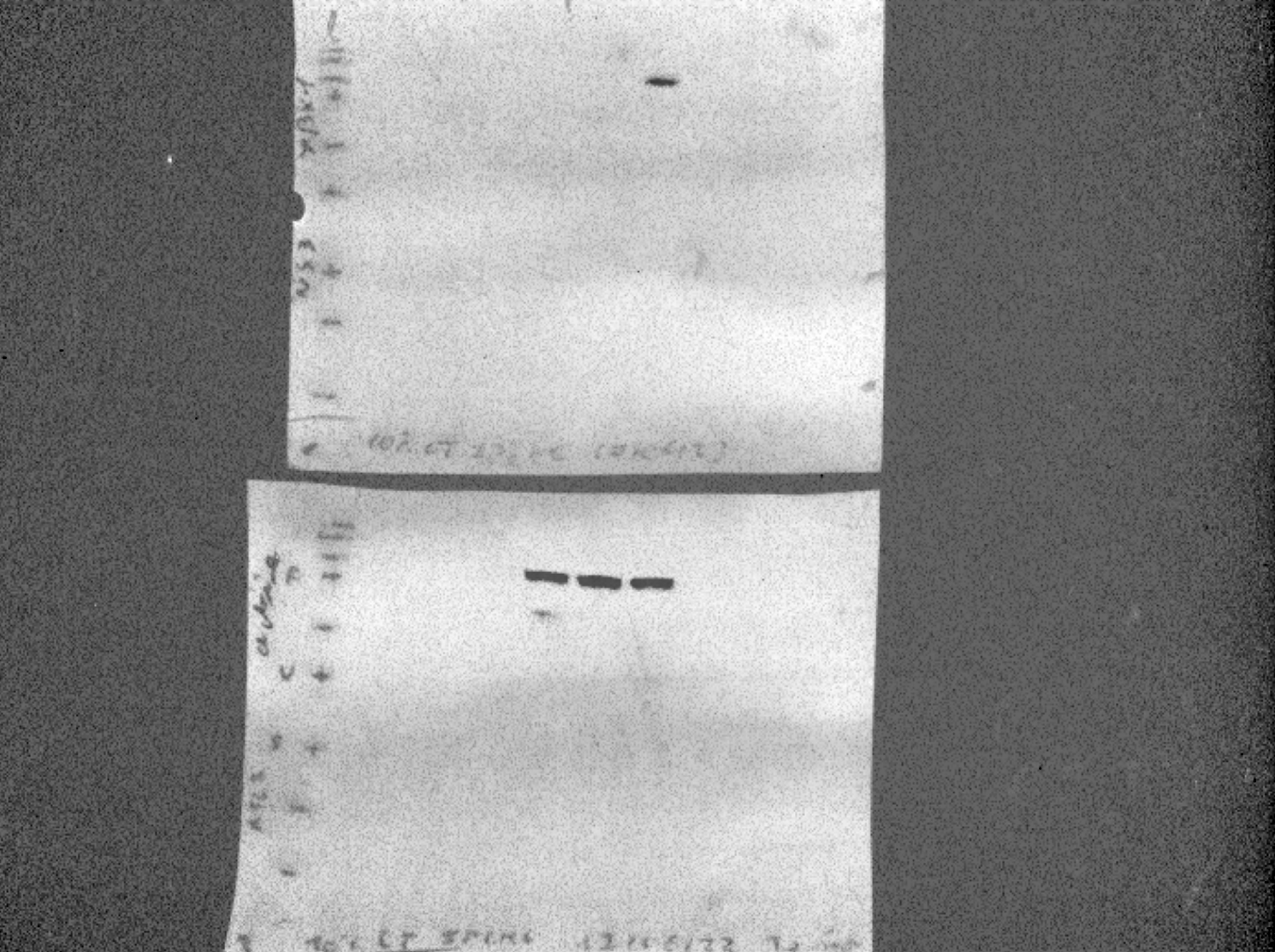

Supplement: Figure 9—figure supplement 2—source data 3. [file elife-94347-fig9-figsupp2-data3.zip › figure 9-figure supplement 2A raw data/NS5/cell extracts/Melany Juarez 2023-06-22 18hr 13min merge.tif]

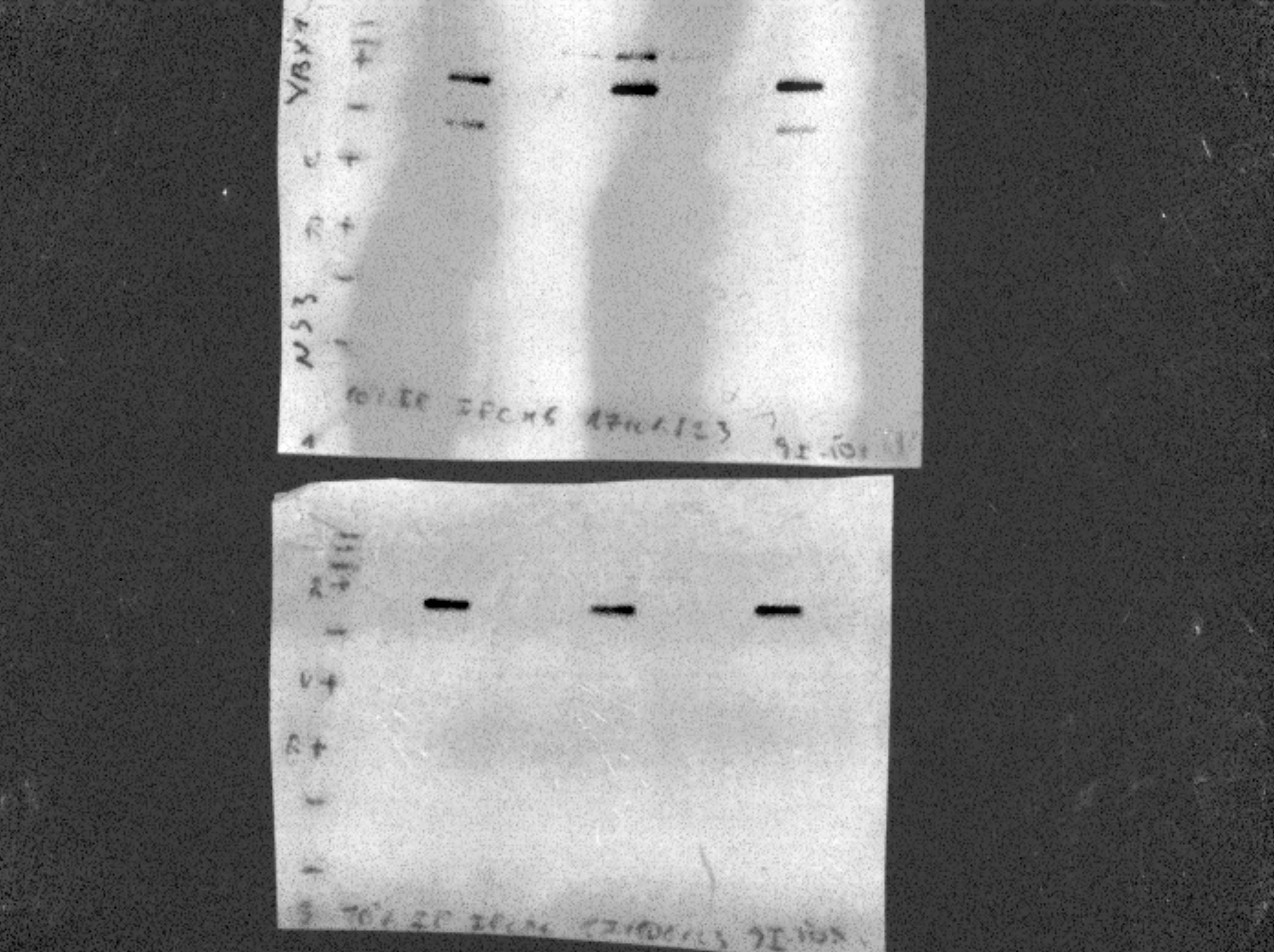

Supplement: Figure 9—figure supplement 2—source data 3. [file elife-94347-fig9-figsupp2-data3.zip › figure 9-figure supplement 2A raw data/NS5/IP anti-HA/merge IP.tif]

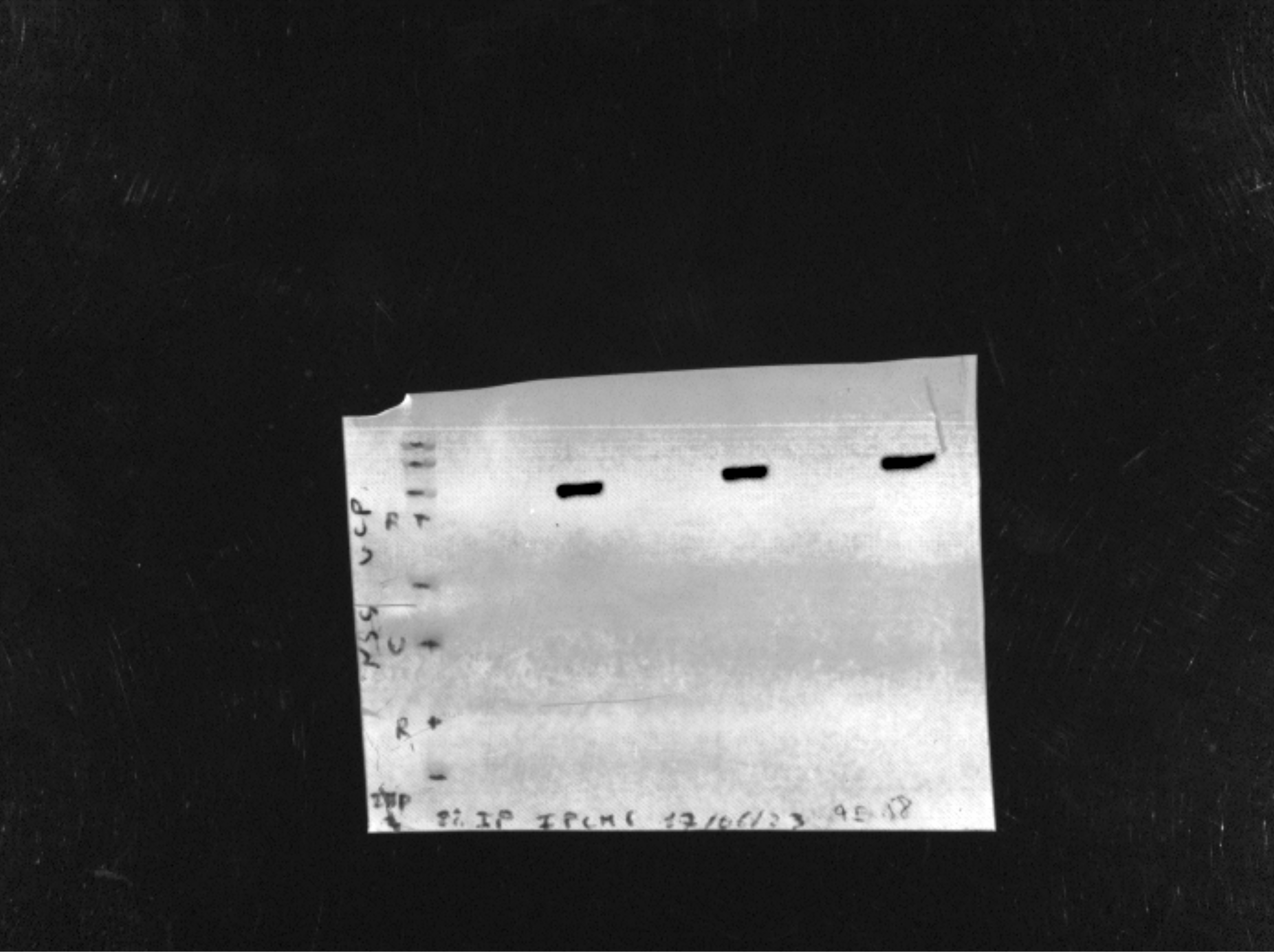

Supplement: Figure 9—figure supplement 2—source data 3. [file elife-94347-fig9-figsupp2-data3.zip › figure 9-figure supplement 2A raw data/VCP/IP anti-HA/Merge IP.tif]

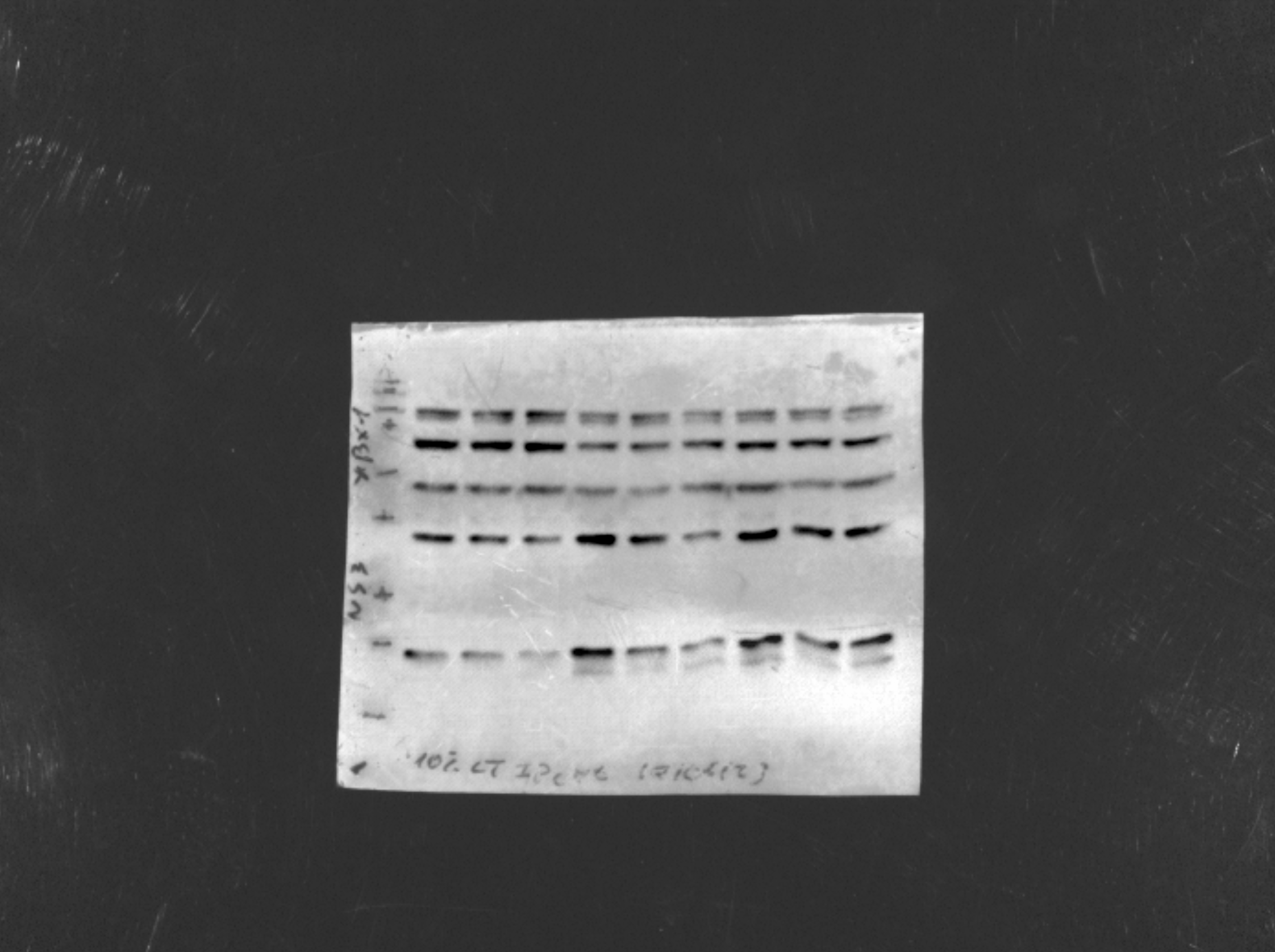

Supplement: Figure 9—figure supplement 2—source data 3. [file elife-94347-fig9-figsupp2-data3.zip › figure 9-figure supplement 2A raw data/YBX1/cell extracts/Melany Juarez 2023-06-21 17hr 02min Merge.tif]

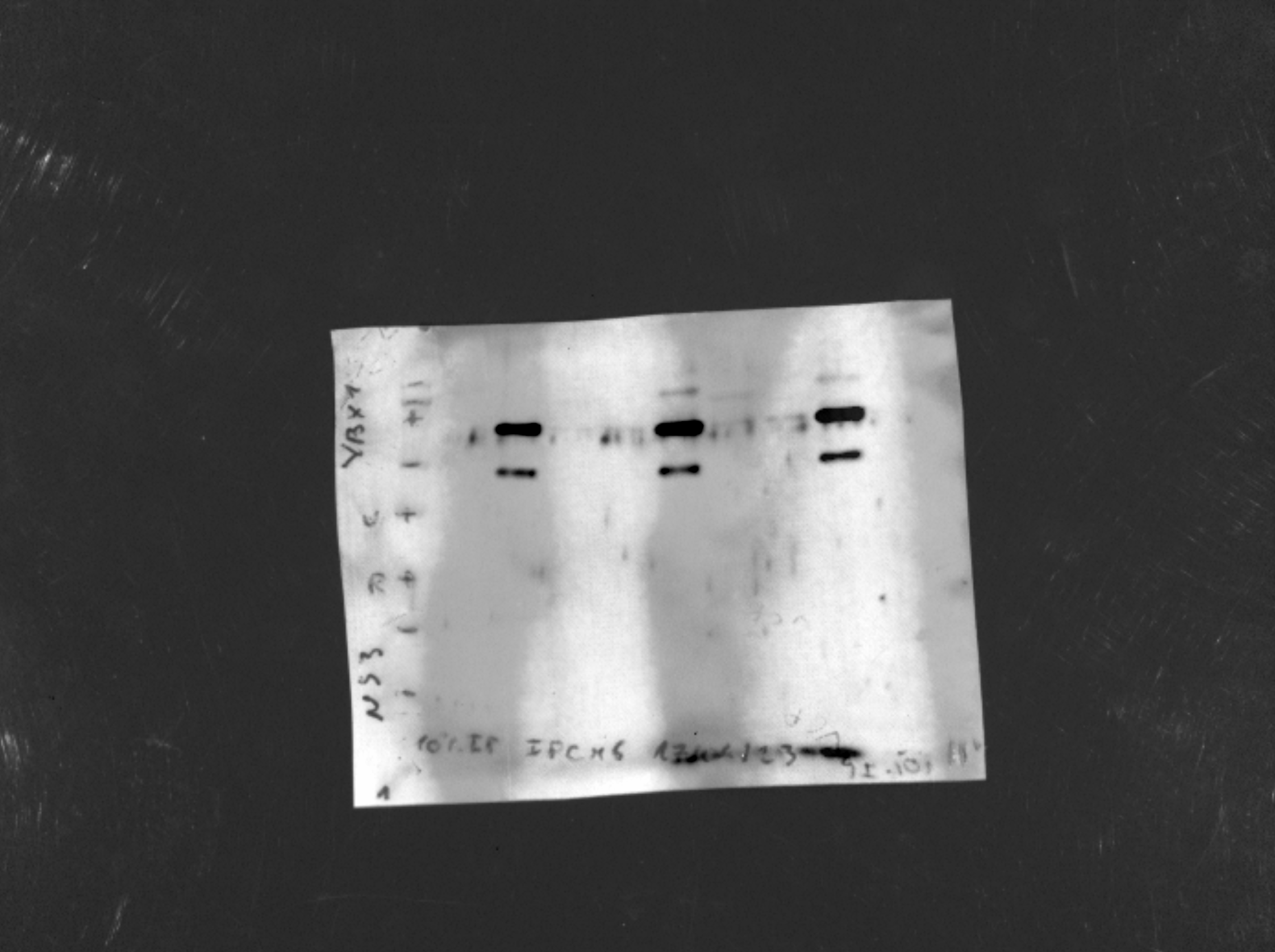

Supplement: Figure 9—figure supplement 2—source data 3. [file elife-94347-fig9-figsupp2-data3.zip › figure 9-figure supplement 2A raw data/YBX1/IP anti-HA/Melany Juarez 2023-06-21 17hr 12min Merge.tif]
