# Supplementary material for: Zika virus remodels and hijacks IGF2BP2 ribonucleoprotein complex to promote viral replication organelle biogenesis
Source: eLife. 2024 Nov 20;13:RP94347. doi: 10.7554/eLife.94347 (PMC11578589; doi:10.7554/eLife.94347)

Figure 4A

Anti-IGF2BP2

1-
2- Mock 48hpi
3- Mock 72hpi
4-
5- ZIKV 48hpi
6- ZIKV 72hpi
7-
8-
9-

Use for figure (2, 3)

Use for figure (5, 6)

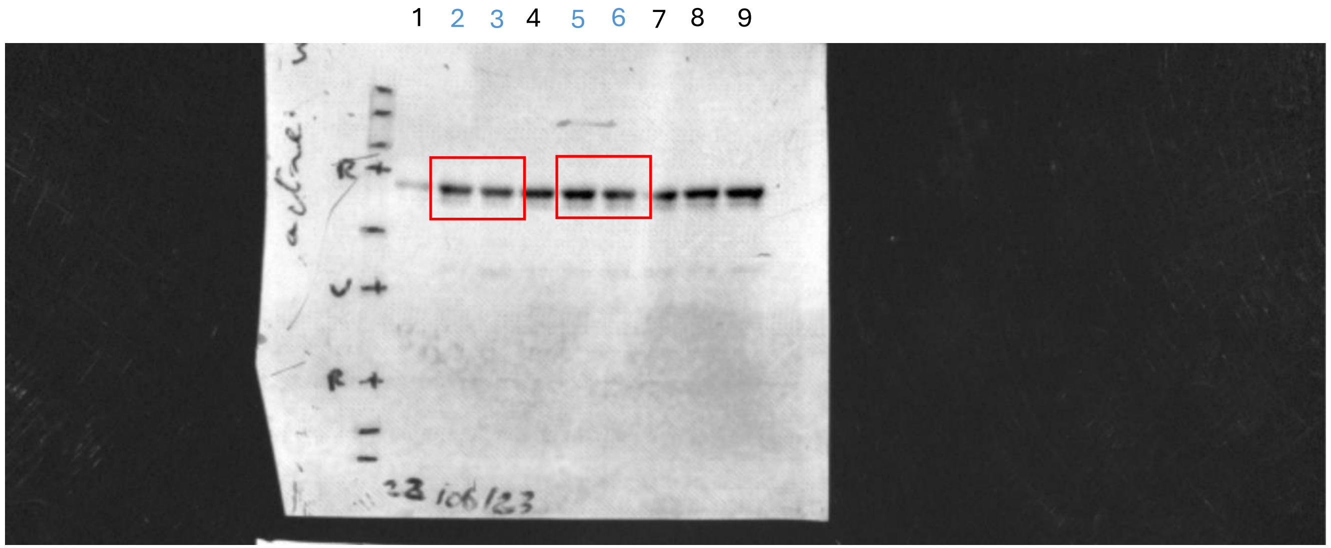

Anti-IGF2BP1

1-
2- Mock 48hpi
3- Mock 72hpi
4-
5- ZIKV 48hpi
6- ZIKV 72hpi
7-
8-
9-

2–3: Use for figure

5–6: Use for figure

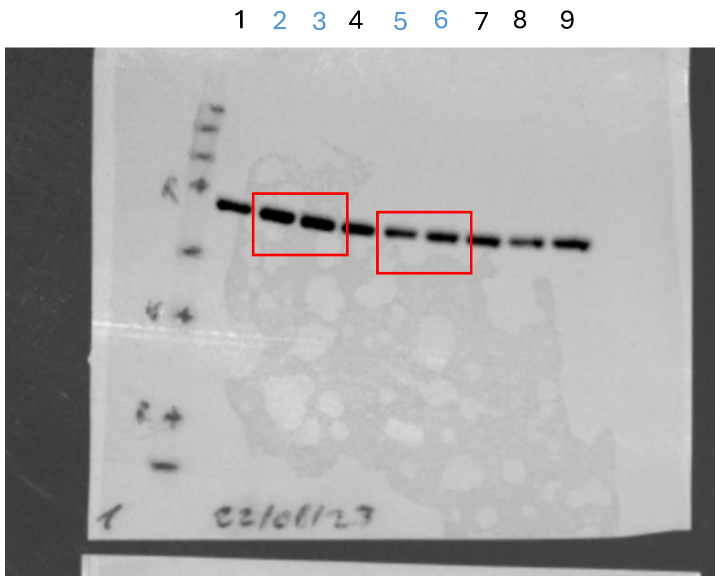

Anti-IGF2BP3

1-
2- Mock 48hpi } Use for figure
3- Mock 72hpi
4-
5- ZIKV 48hpi } Use for figure
6- ZIKV 72hpi
7-
8-
9-

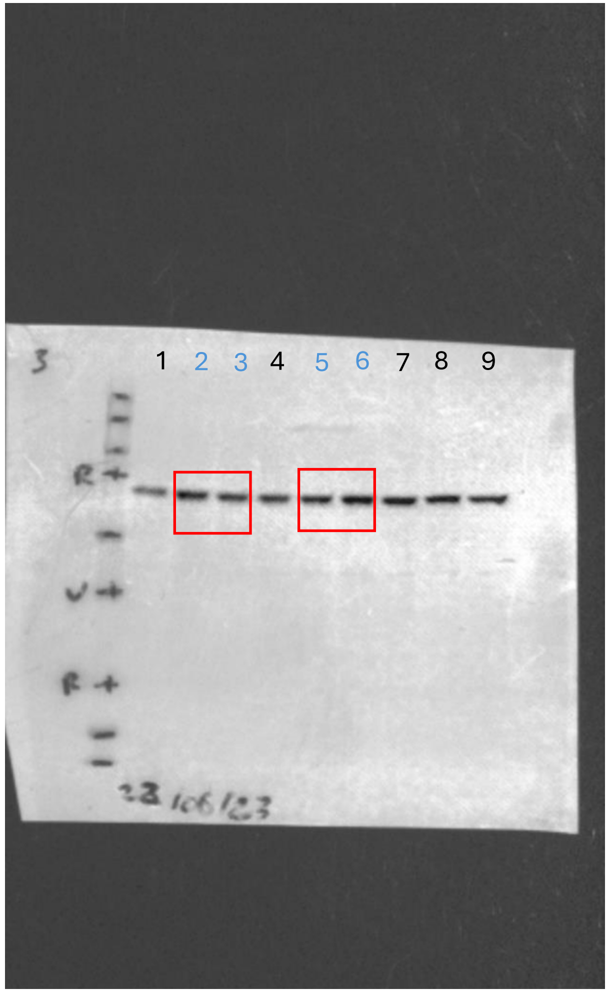

Anti-DDX3

1-
2- Mock 48hpi
3- Mock 72hpi
Use for figure
4-
5- ZIKV 48hpi
6- ZIKV 72hpi
Use for figure
7-
8-
9-

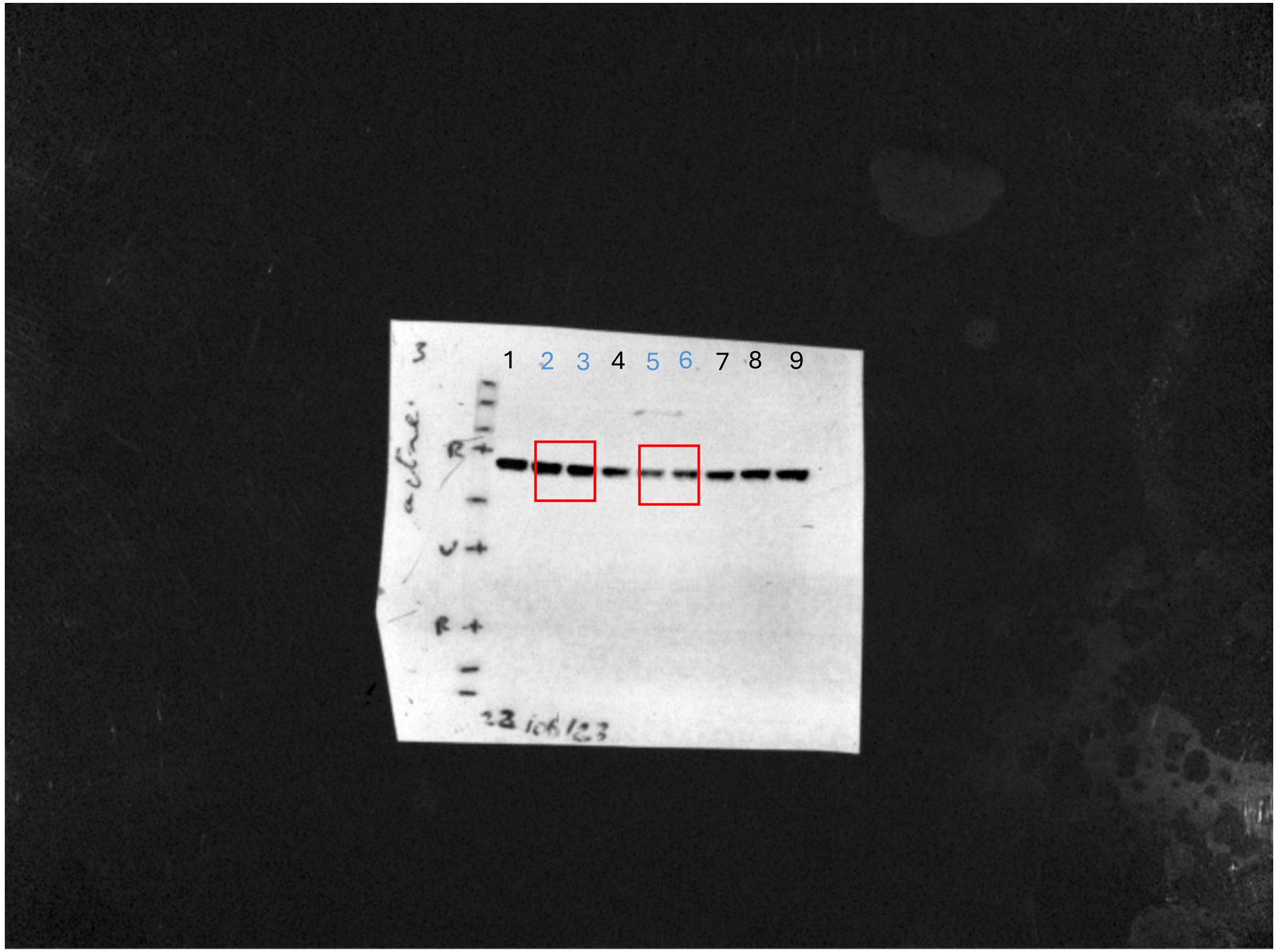

Anti-DDX5

1-
2- Mock 48hpi
3- Mock 72hpi
Use for figure
4-
5- ZIKV 48hpi
6- ZIKV 72hpi
Use for figure
7-
8-
9-

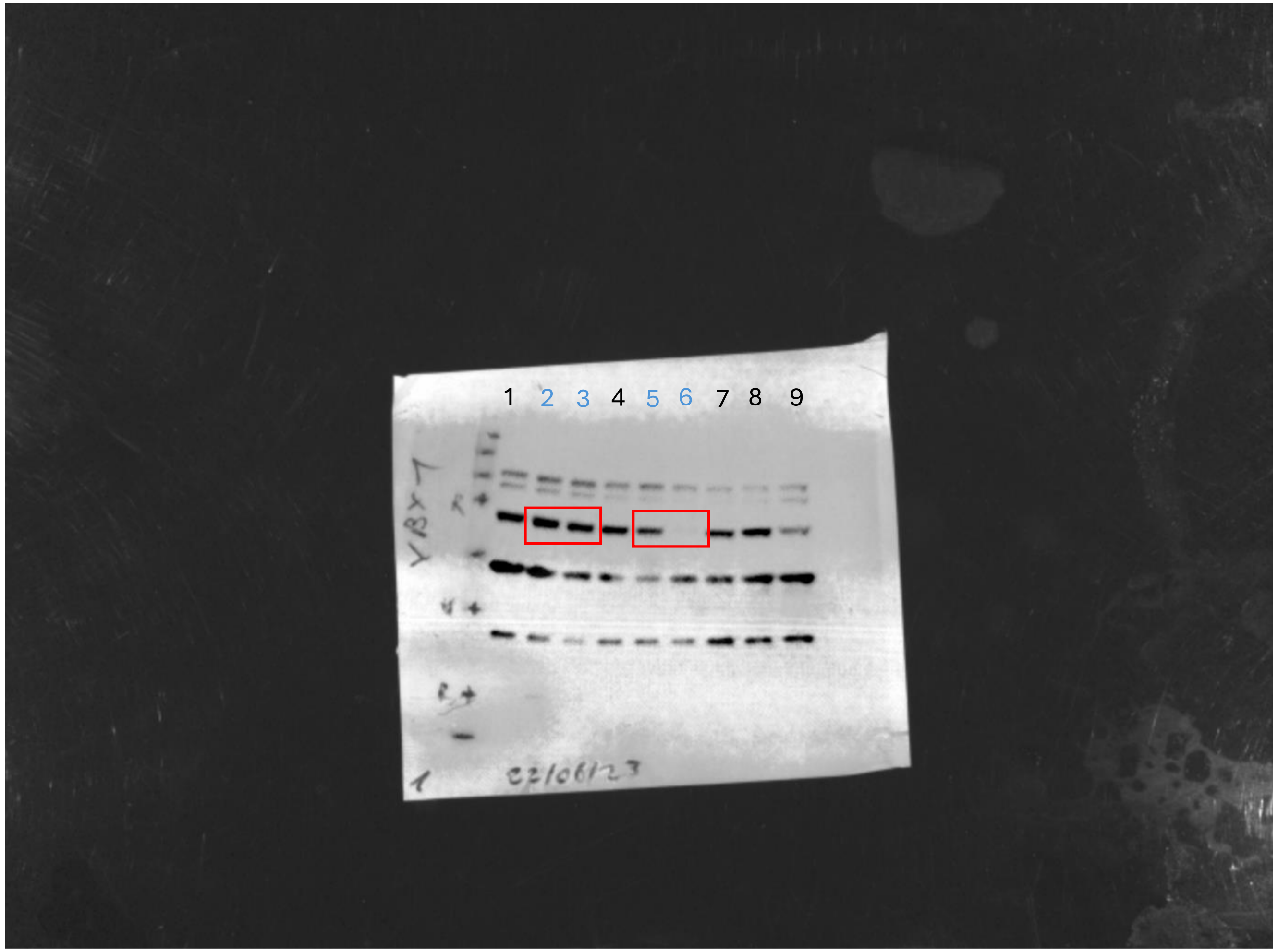

Anti-NS3

1-
2- Mock 48hpi
3- Mock 72hpi
Use for figure
4-
5- ZIKV 48hpi
6- ZIKV 72hpi
Use for figure
7-
8-
9-

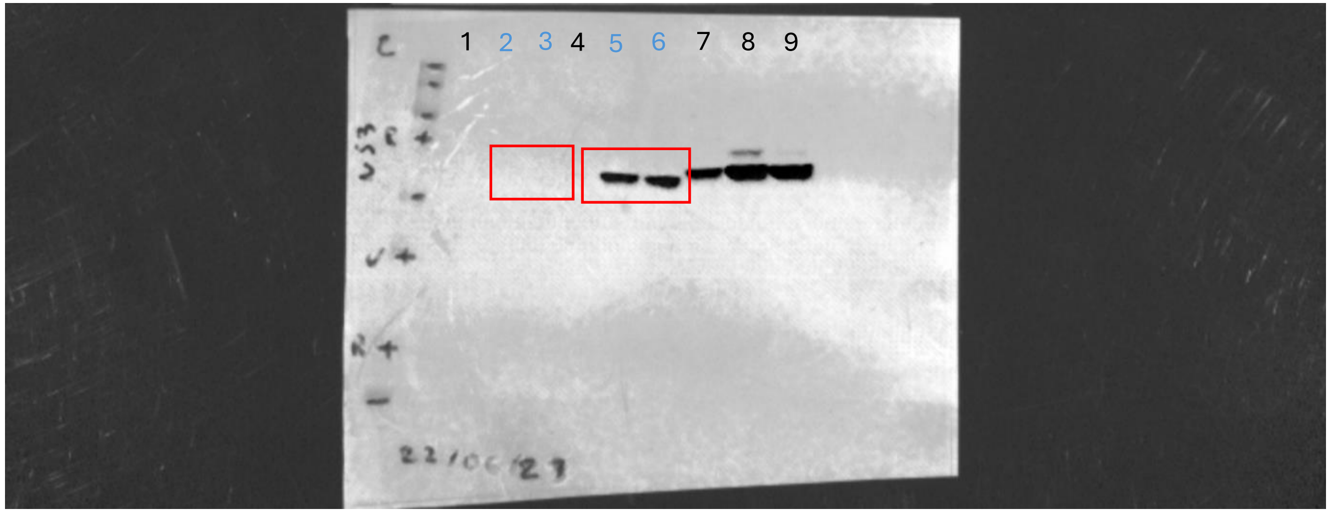

Anti-actin

1-
2- Mock 48hpi
3- Mock 72hpi
4-
5- ZIKV 48hpi
6- ZIKV 72hpi
7-
8-
9-

Use for figure (2, 3)

Use for figure (5, 6)

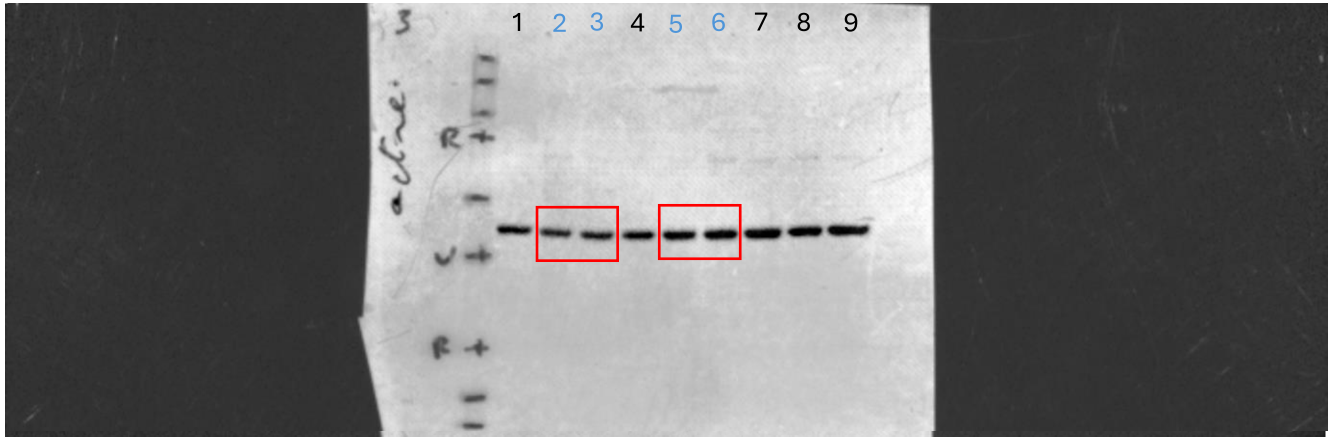

Figure 4C

Anti-HA

Cell extracts

1- Mock IGF2BP2-HA (-)
2- Mock IGF2BP2-HA (+)
3- ZIKV H/PF/2013 IGF2BP2-HA (-)
4- ZIKV H/PF/2013 IGF2BP2-HA (+)
5- ZIKV MR766 IGF2BP2-HA (-)
6- ZIKV MR766 IGF2BP2-HA (+)

Use for figure

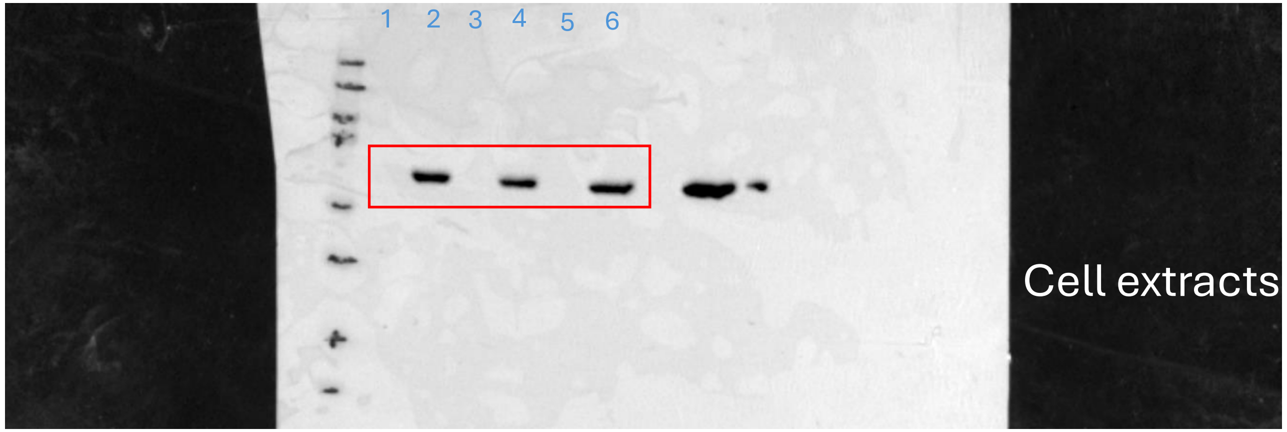

Anti-IGF2BP2

Cell extracts

1- Mock IGF2BP2-HA (-)
2- Mock IGF2BP2-HA (+)
3- ZIKV H/PF/2013 IGF2BP2-HA (-)
4- ZIKV H/PF/2013 IGF2BP2-HA (+)
5- ZIKV MR766 IGF2BP2-HA (-)
6- ZIKV MR766 IGF2BP2-HA (+)

Use for figure

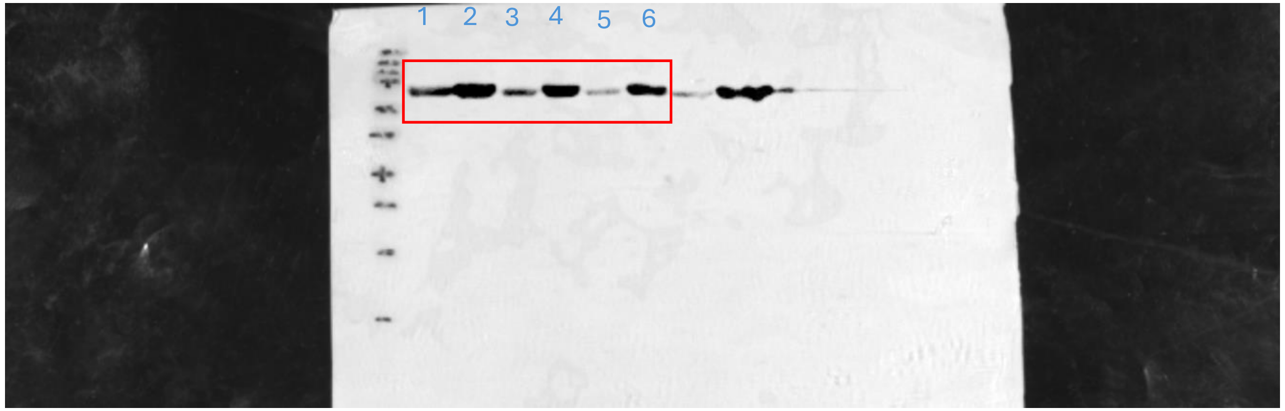

Anti-NS5 ZIKV

Cell extracts

1- Mock IGF2BP2-HA (-)
2- Mock IGF2BP2-HA (+)
3- ZIKV H/PF/2013 IGF2BP2-HA (-)
4- ZIKV H/PF/2013 IGF2BP2-HA (+)
5- ZIKV MR766 IGF2BP2-HA (-)
6- ZIKV MR766 IGF2BP2-HA (+)

Use for figure

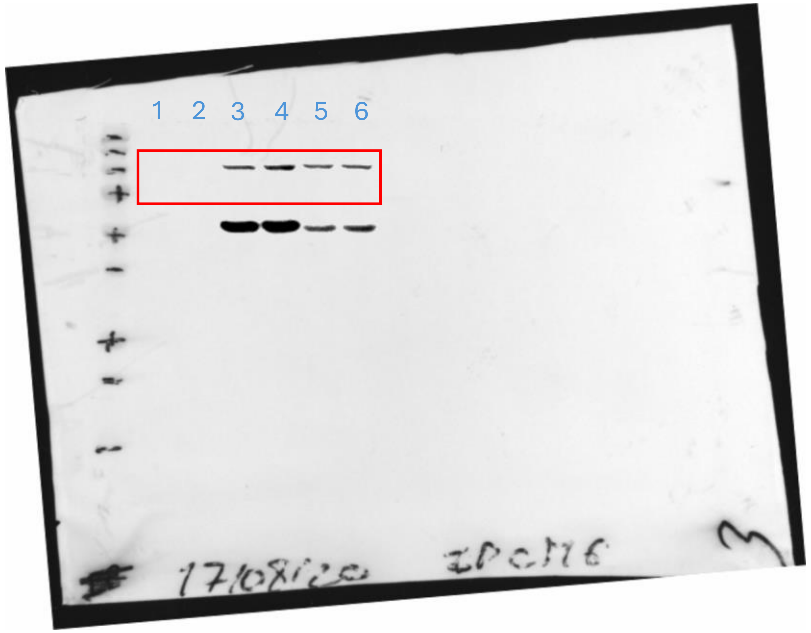

Anti-NS3

Cell extracts

1- Mock IGF2BP2-HA (-)
2- Mock IGF2BP2-HA (+)
3- ZIKV H/PF/2013 IGF2BP2-HA (-)
4- ZIKV H/PF/2013 IGF2BP2-HA (+)
5- ZIKV MR766 IGF2BP2-HA (-)
6- ZIKV MR766 IGF2BP2-HA (+)

Use for figure

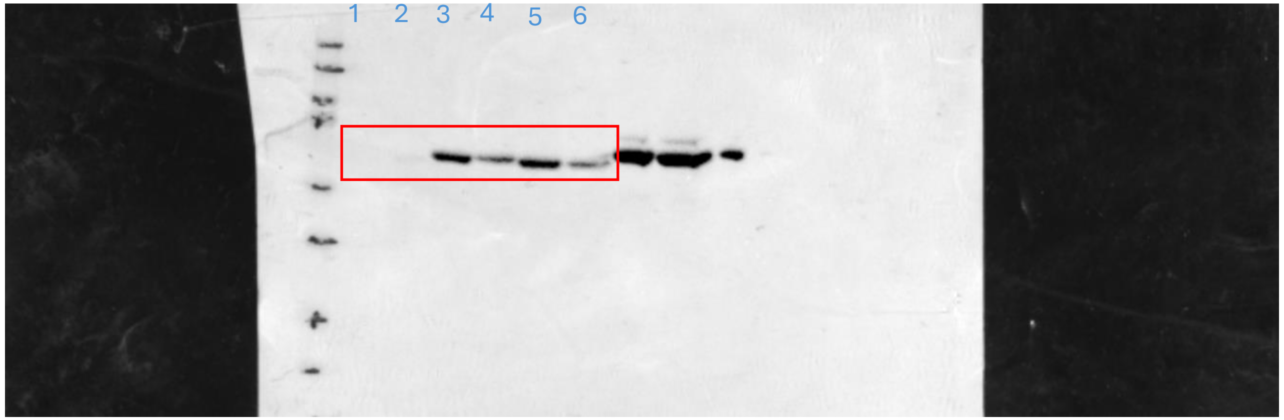

Anti-NS5 ZIKV

Cell extracts

1- Mock IGF2BP2-HA (-)
2- Mock IGF2BP2-HA (+)
3- ZIKV H/PF/2013 IGF2BP2-HA (-)
4- ZIKV H/PF/2013 IGF2BP2-HA (+)
5- ZIKV MR766 IGF2BP2-HA (-)
6- ZIKV MR766 IGF2BP2-HA (+)

Use for figure

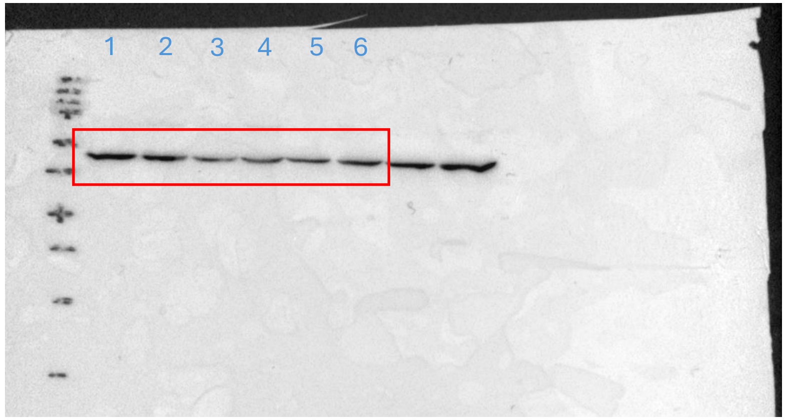

Anti-HA

IP anti-HA

1- Mock IGF2BP2-HA (-)
2- Mock IGF2BP2-HA (+)
3- ZIKV H/PF/2013 IGF2BP2-HA (-)
4- ZIKV H/PF/2013 IGF2BP2-HA (+)
5- ZIKV MR766 IGF2BP2-HA (-)
6- ZIKV MR766 IGF2BP2-HA (+)

Use for figure

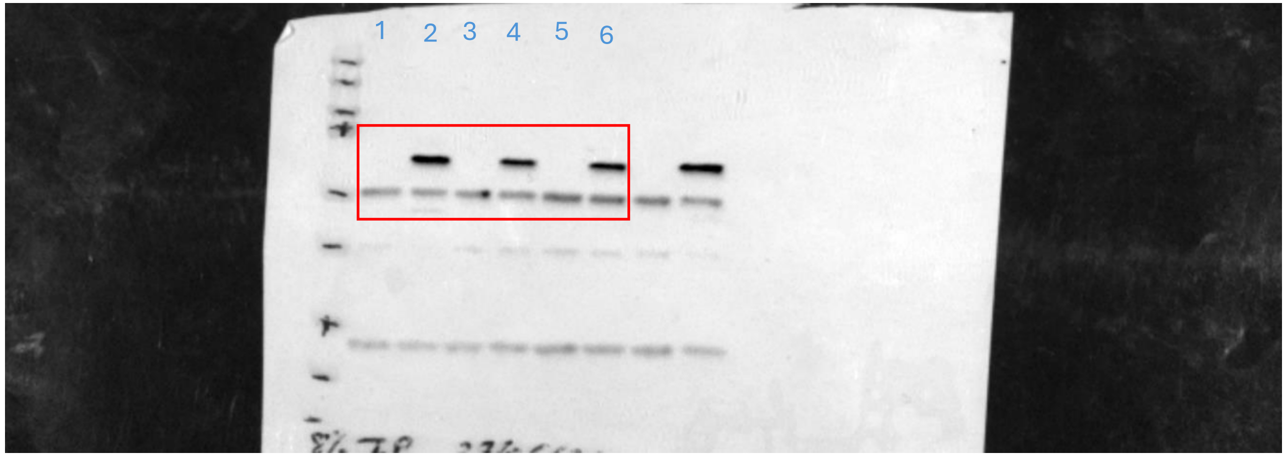

Anti-IGF2BP2

IP anti-HA

1- Mock IGF2BP2-HA (-)
2- Mock IGF2BP2-HA (+)
3- ZIKV H/PF/2013 IGF2BP2-HA (-)
4- ZIKV H/PF/2013 IGF2BP2-HA (+)
5- ZIKV MR766 IGF2BP2-HA (-)
6- ZIKV MR766 IGF2BP2-HA (+)

Use for figure

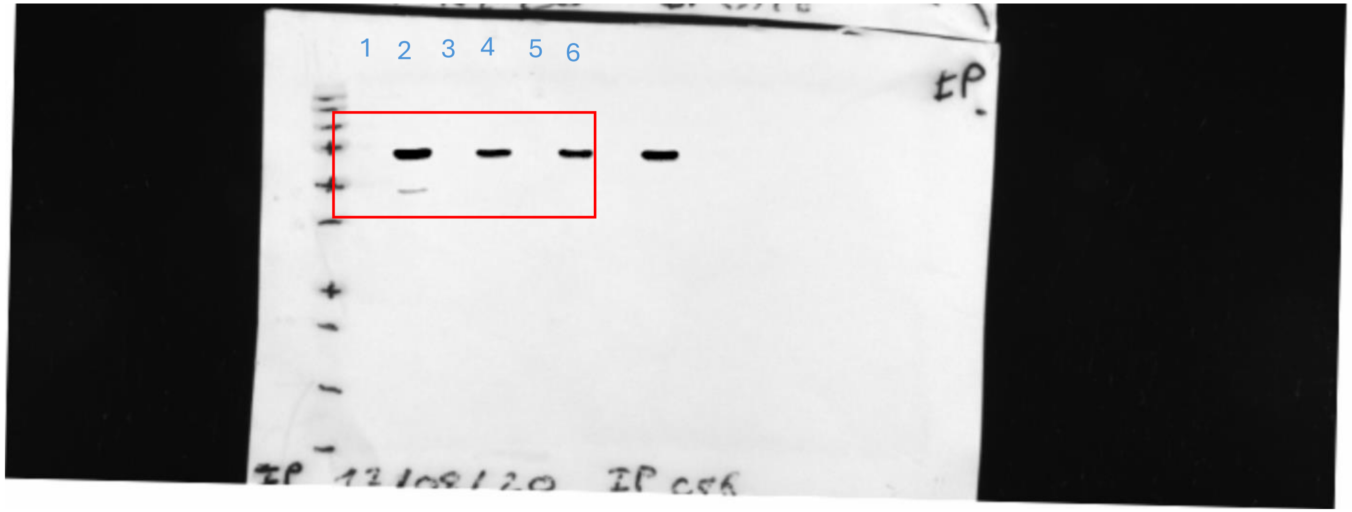

Anti-NS5

IP anti-HA

1- Mock IGF2BP2-HA (-)
2- Mock IGF2BP2-HA (+)
3- ZIKV H/PF/2013 IGF2BP2-HA (-)
4- ZIKV H/PF/2013 IGF2BP2-HA (+)
5- ZIKV MR766 IGF2BP2-HA (-)
6- ZIKV MR766 IGF2BP2-HA (+)

Use for figure

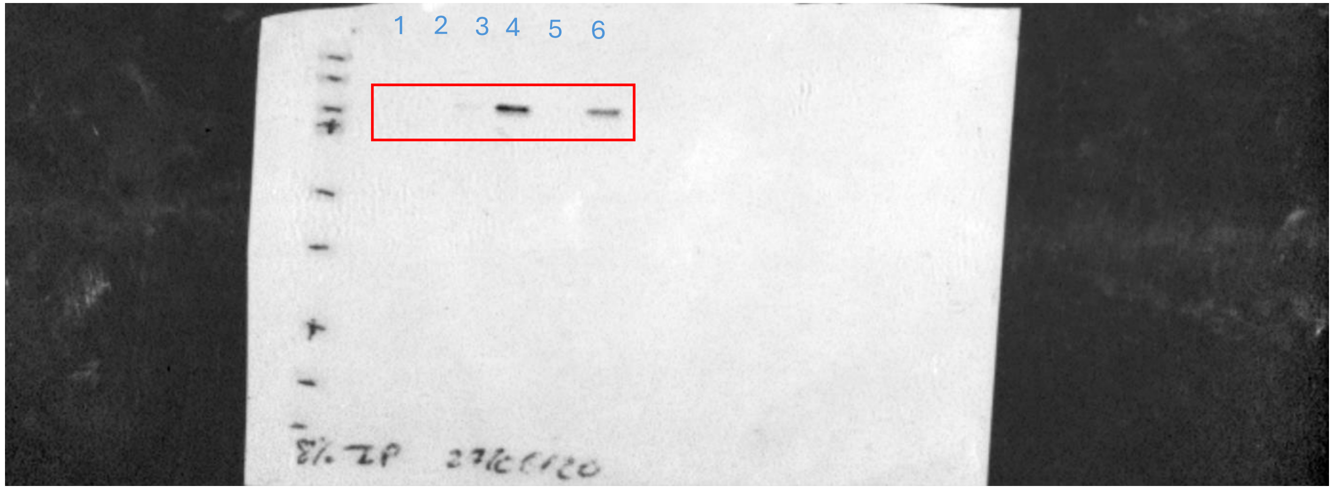

Anti-NS3

IP anti-HA

1- Mock IGF2BP2-HA (-)
2- Mock IGF2BP2-HA (+)
3- ZIKV H/PF/2013 IGF2BP2-HA (-)
4- ZIKV H/PF/2013 IGF2BP2-HA (+)
5- ZIKV MR766 IGF2BP2-HA (-)
6- ZIKV MR766 IGF2BP2-HA (+)

Use for figure

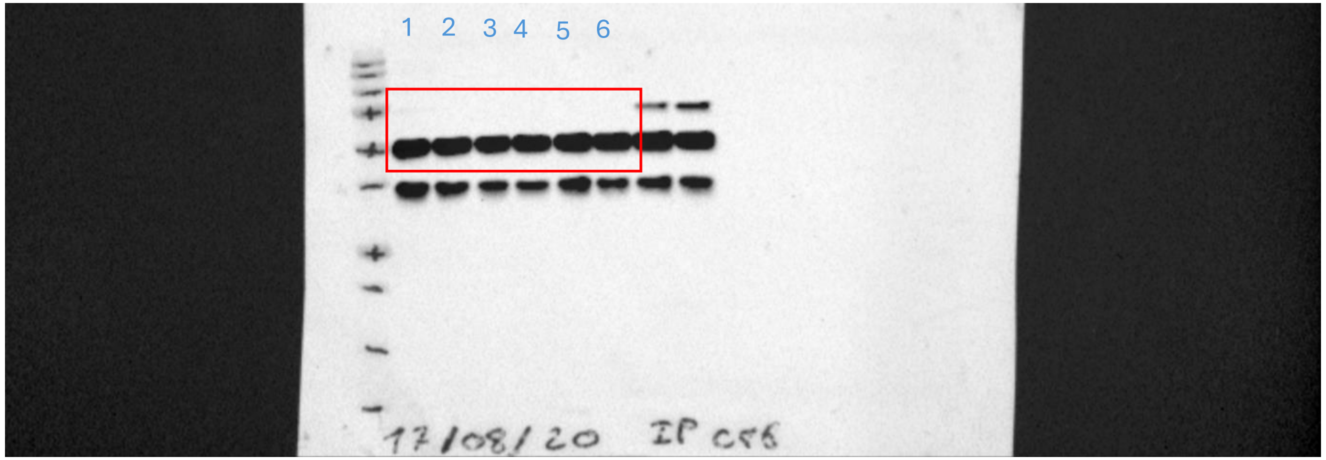

Supplement: Figure 4—source data 2. [file elife-94347-fig4-data2.zip › Figure 4-source data 2.pdf]
