# Supplementary material for: Zika virus remodels and hijacks IGF2BP2 ribonucleoprotein complex to promote viral replication organelle biogenesis
Source: eLife. 2024 Nov 20;13:RP94347. doi: 10.7554/eLife.94347 (PMC11578589; doi:10.7554/eLife.94347)

# Figure 8A

Anti-HA

Cell extracts

1- Mock IGF2BP2-HA (-)
2- Mock IGF2BP2-HA (+)
3- ZIKV H/PF/2013 IGF2BP2-HA (-)
4- ZIKV H/PF/2013 IGF2BP2-HA (+)

Use for figure

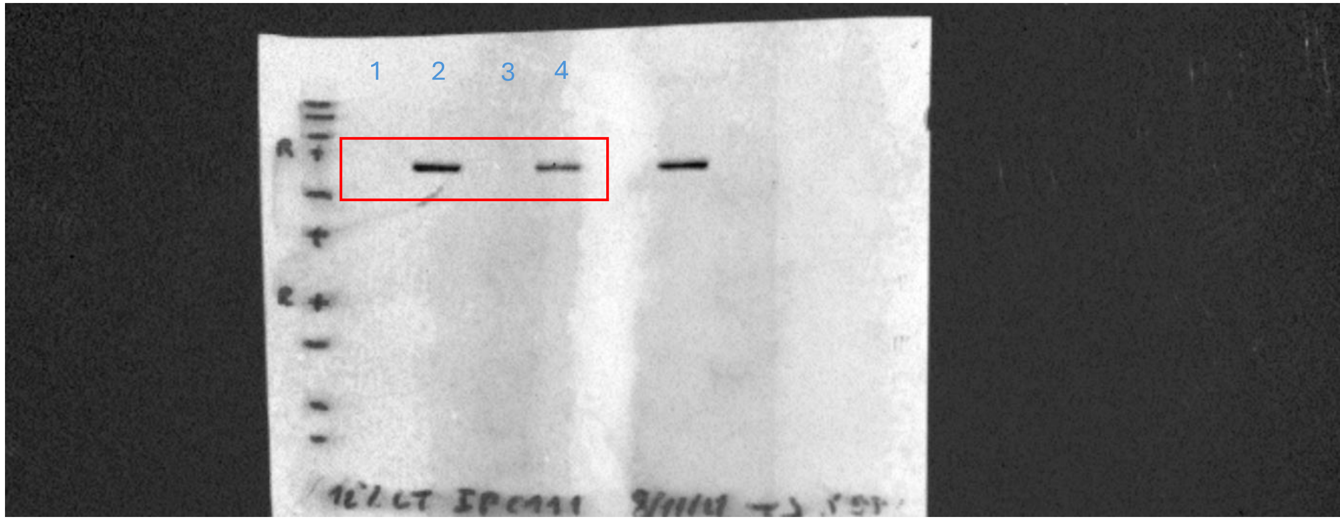

## Anti-IGF2BP2

Cell extracts

1- Mock IGF2BP2-HA (-)
2- Mock IGF2BP2-HA (+)
3- ZIKV H/PF/2013 IGF2BP2-HA (-)
4- ZIKV H/PF/2013 IGF2BP2-HA (+)

Use for figure

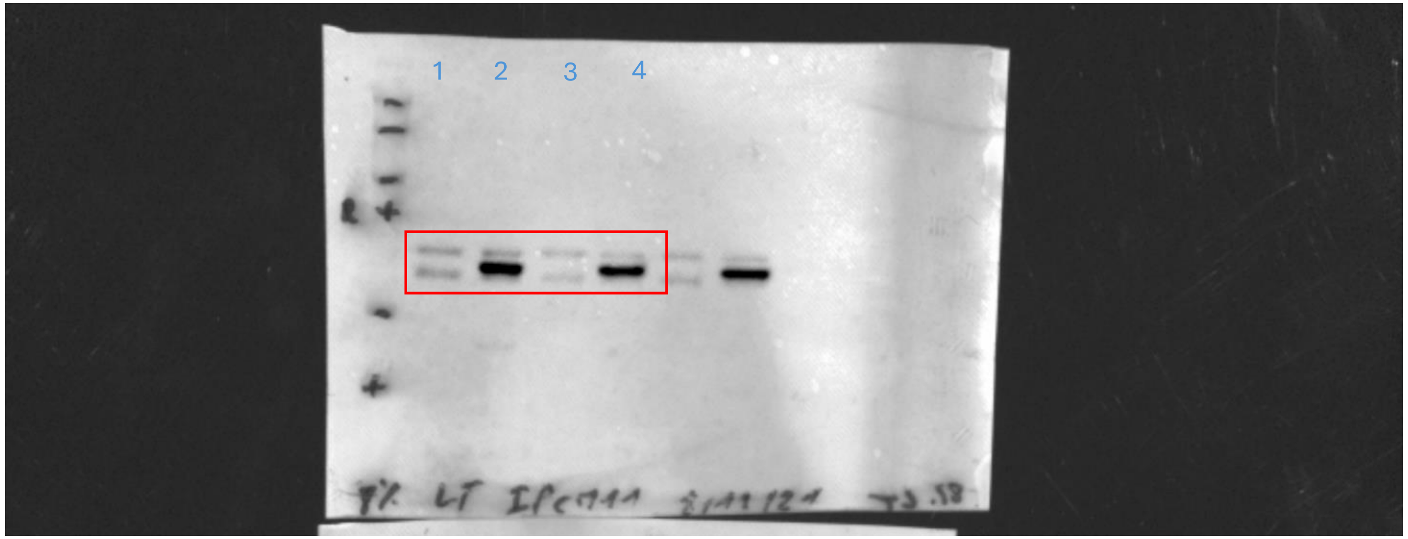

Anti-IGF2BP1

Cell extracts

1- Mock IGF2BP2-HA (-)
2- Mock IGF2BP2-HA (+)
3- ZIKV H/PF/2013 IGF2BP2-HA (-)
4- ZIKV H/PF/2013 IGF2BP2-HA (+)

Use for figure (lanes 1–4)

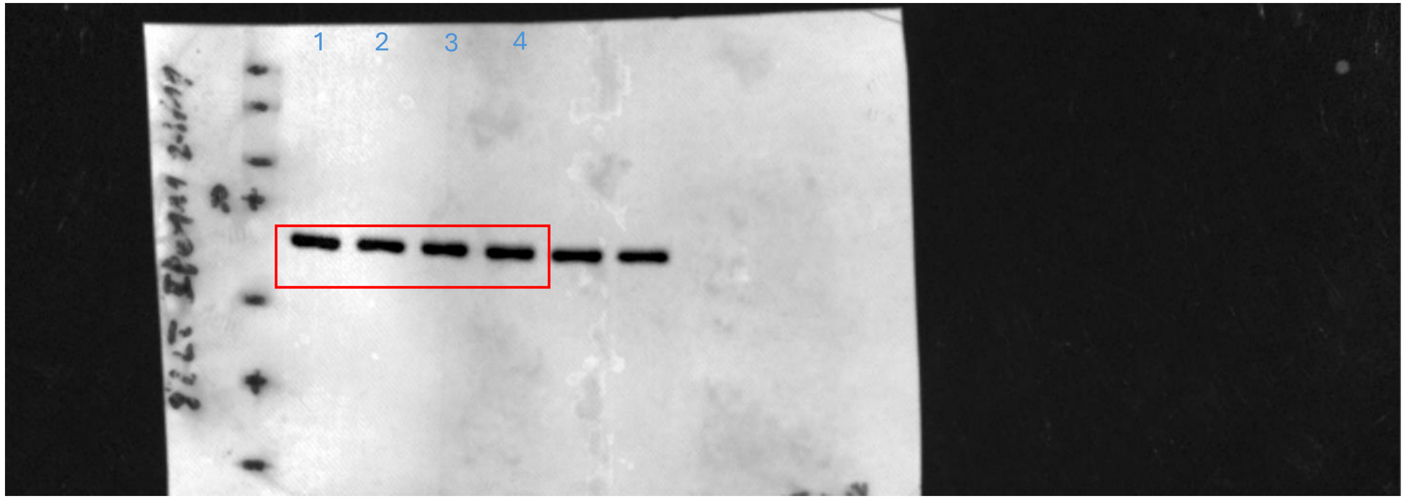

Anti-IGF2BP3

Cell extracts

1- Mock IGF2BP2-HA (-)
2- Mock IGF2BP2-HA (+)
3- ZIKV H/PF/2013 IGF2BP2-HA (-)
4- ZIKV H/PF/2013 IGF2BP2-HA (+)

Use for figure

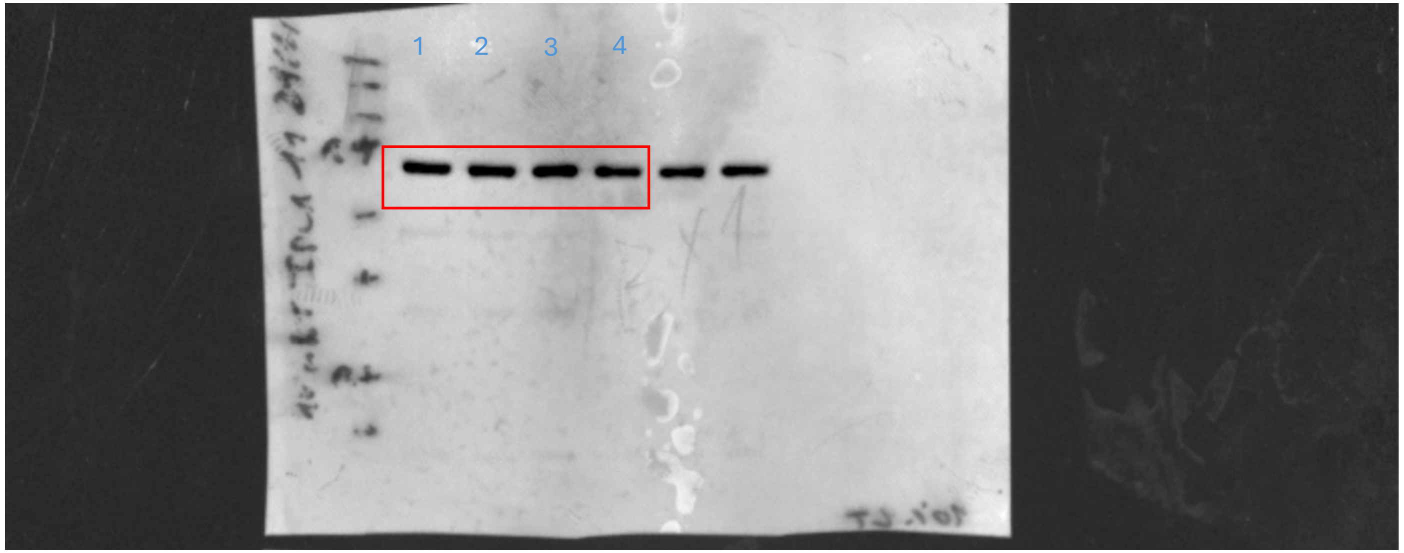

Anti-YBX1

Cell extracts

1- Mock IGF2BP2-HA (-)
2- Mock IGF2BP2-HA (+)
3- ZIKV H/PF/2013 IGF2BP2-HA (-)
4- ZIKV H/PF/2013 IGF2BP2-HA (+)

Use for figure

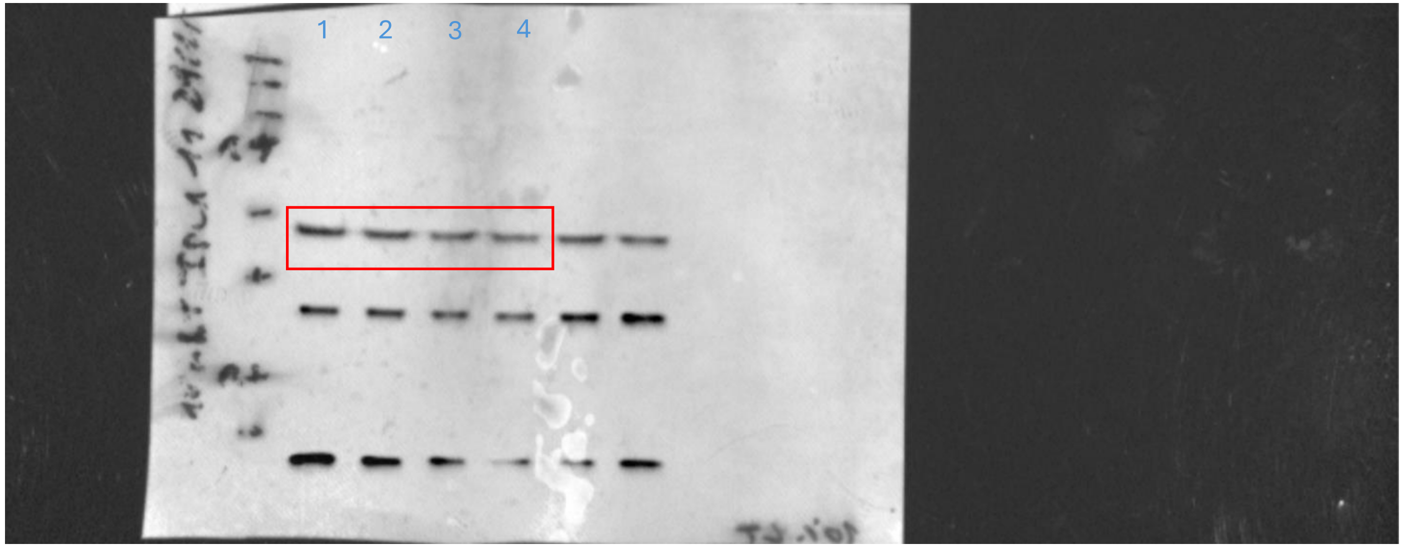

Anti-NS5 ZIKV

Cell extracts

1- Mock IGF2BP2-HA (-)
2- Mock IGF2BP2-HA (+)
3- ZIKV H/PF/2013 IGF2BP2-HA (-)
4- ZIKV H/PF/2013 IGF2BP2-HA (+)

Use for figure

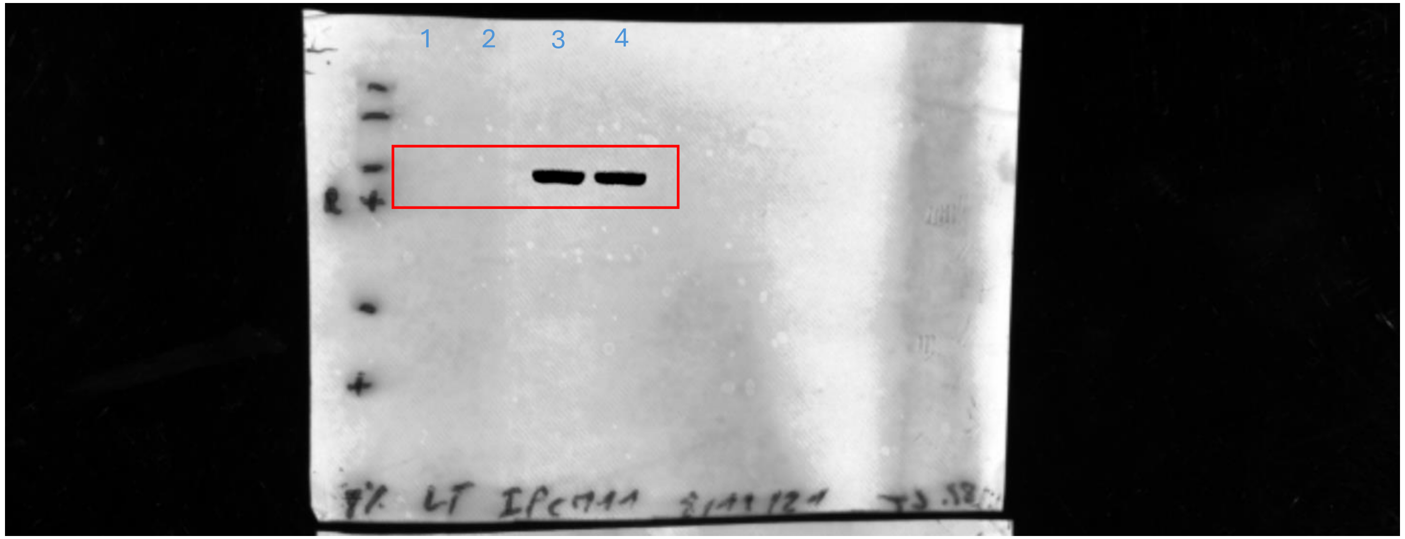

## Anti-actin

Cell extracts

1- Mock IGF2BP2-HA (-)
2- Mock IGF2BP2-HA (+)
3- ZIKV H/PF/2013 IGF2BP2-HA (-)
4- ZIKV H/PF/2013 IGF2BP2-HA (+)

Use for figure

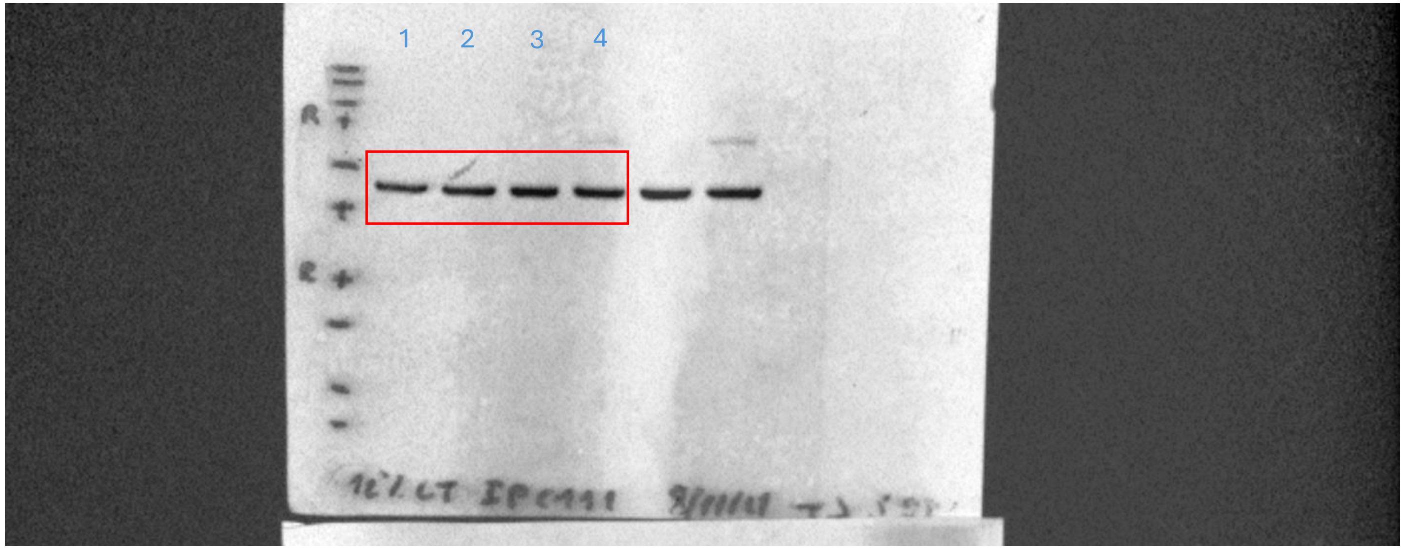

Anti-HA

IP anti HA

1- Mock IGF2BP2-HA (-)
2- Mock IGF2BP2-HA (+)
3- ZIKV H/PF/2013 IGF2BP2-HA (-)
4- ZIKV H/PF/2013 IGF2BP2-HA (+)

Use for figure

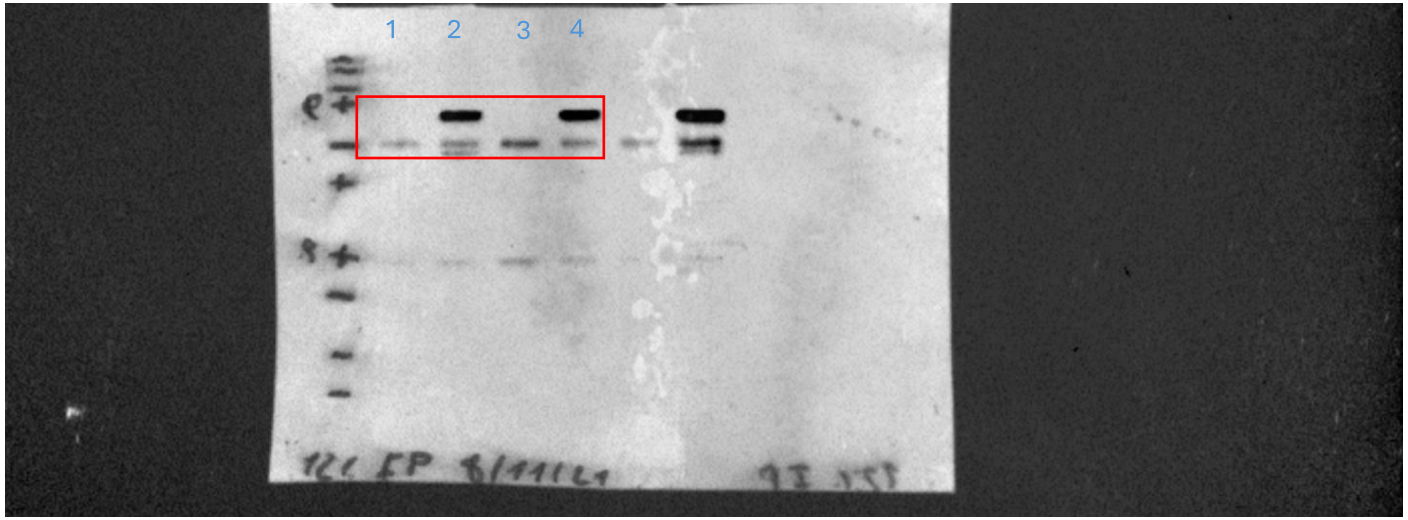

Anti-IGF2BP2

IP anti-HA

1- Mock IGF2BP2-HA (-)
2- Mock IGF2BP2-HA (+)
3- ZIKV H/PF/2013 IGF2BP2-HA (-)
4- ZIKV H/PF/2013 IGF2BP2-HA (+)

Use for figure

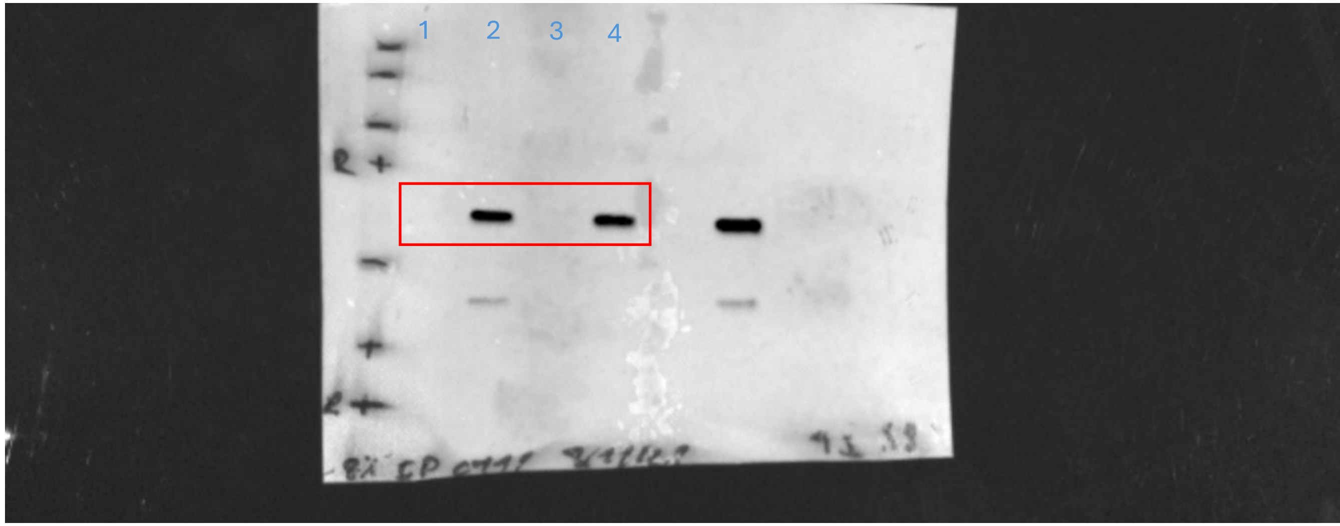

Anti-IGF2BP1

IP anti-HA

1- Mock IGF2BP2-HA (-)
2- Mock IGF2BP2-HA (+)
3- ZIKV H/PF/2013 IGF2BP2-HA (-)
4- ZIKV H/PF/2013 IGF2BP2-HA (+)

Use for figure

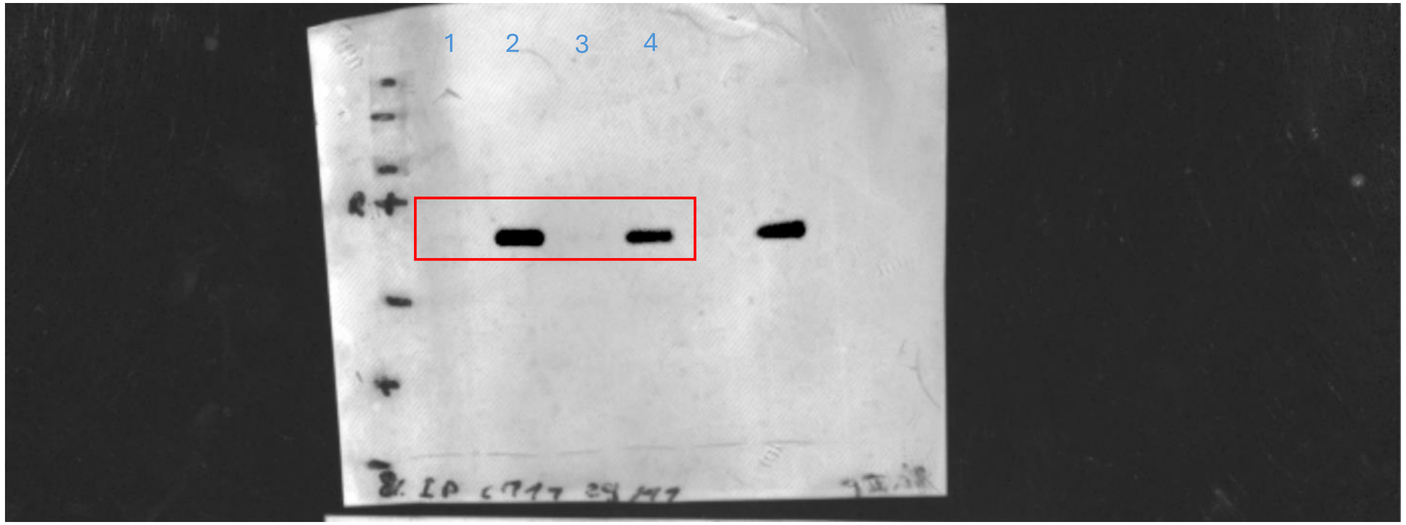

Anti-IGF2BP3

IP anti-HA

1- Mock IGF2BP2-HA (-)
2- Mock IGF2BP2-HA (+)
3- ZIKV H/PF/2013 IGF2BP2-HA (-)
4- ZIKV H/PF/2013 IGF2BP2-HA (+)

Use for figure

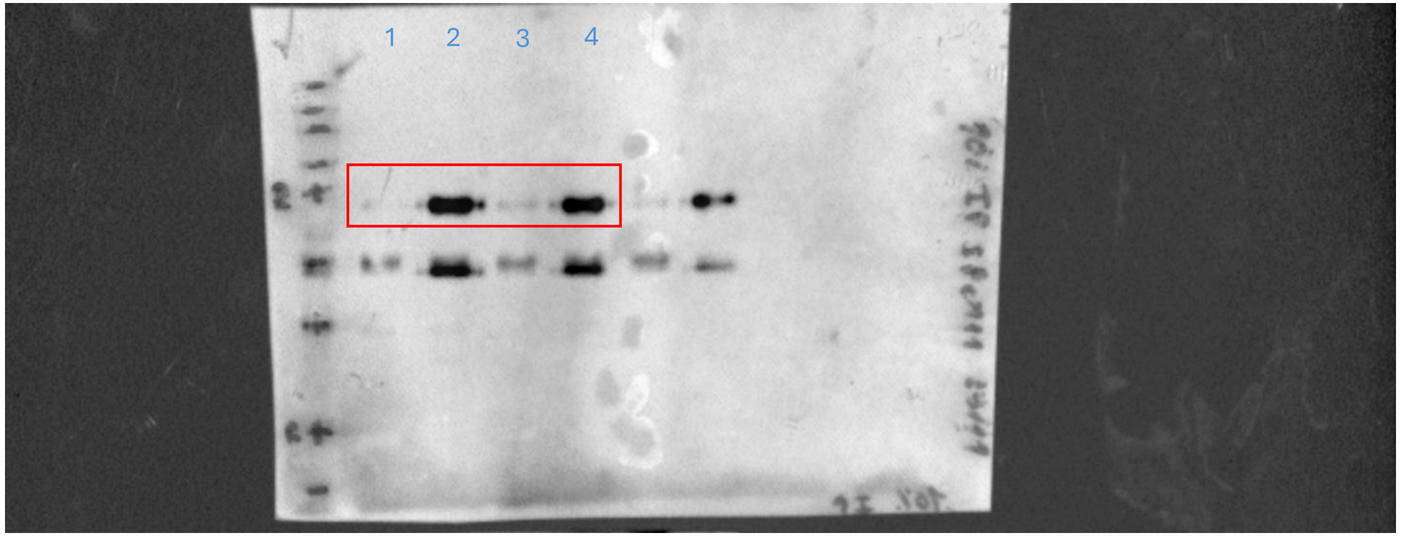

Anti-YBX1

IP anti-HA

1- Mock IGF2BP2-HA (-)
2- Mock IGF2BP2-HA (+)
3- ZIKV H/PF/2013 IGF2BP2-HA (-)
4- ZIKV H/PF/2013 IGF2BP2-HA (+)

Use for figure

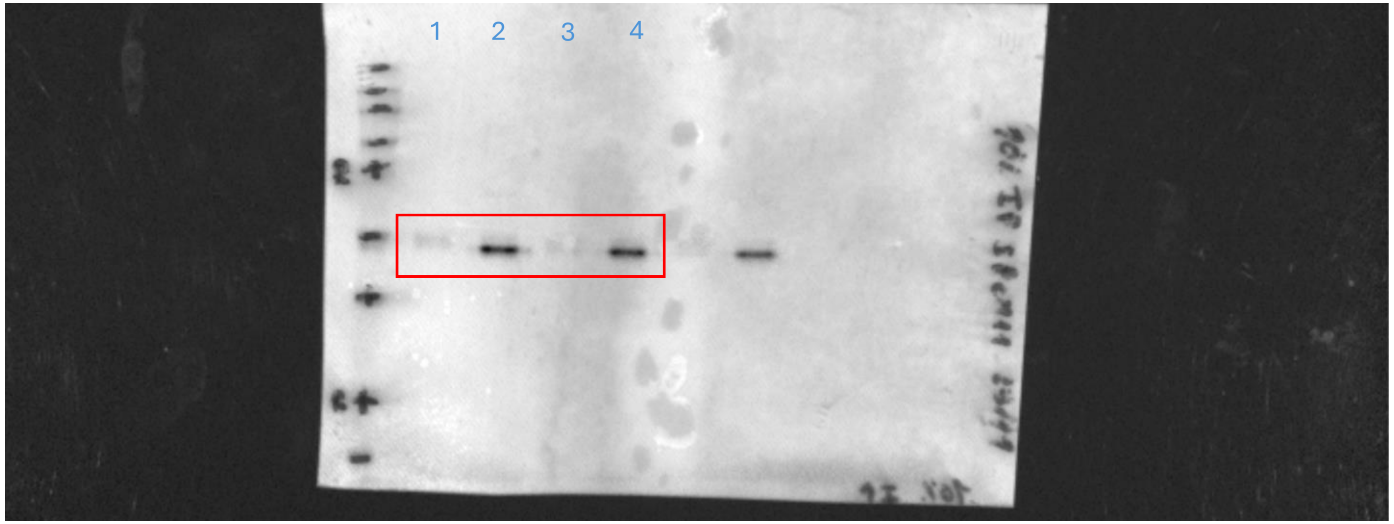

Anti-NS5 ZIKV

IP anti-HA

1- Mock IGF2BP2-HA (-)
2- Mock IGF2BP2-HA (+)
3- ZIKV H/PF/2013 IGF2BP2-HA (-)
4- ZIKV H/PF/2013 IGF2BP2-HA (+)

Use for figure

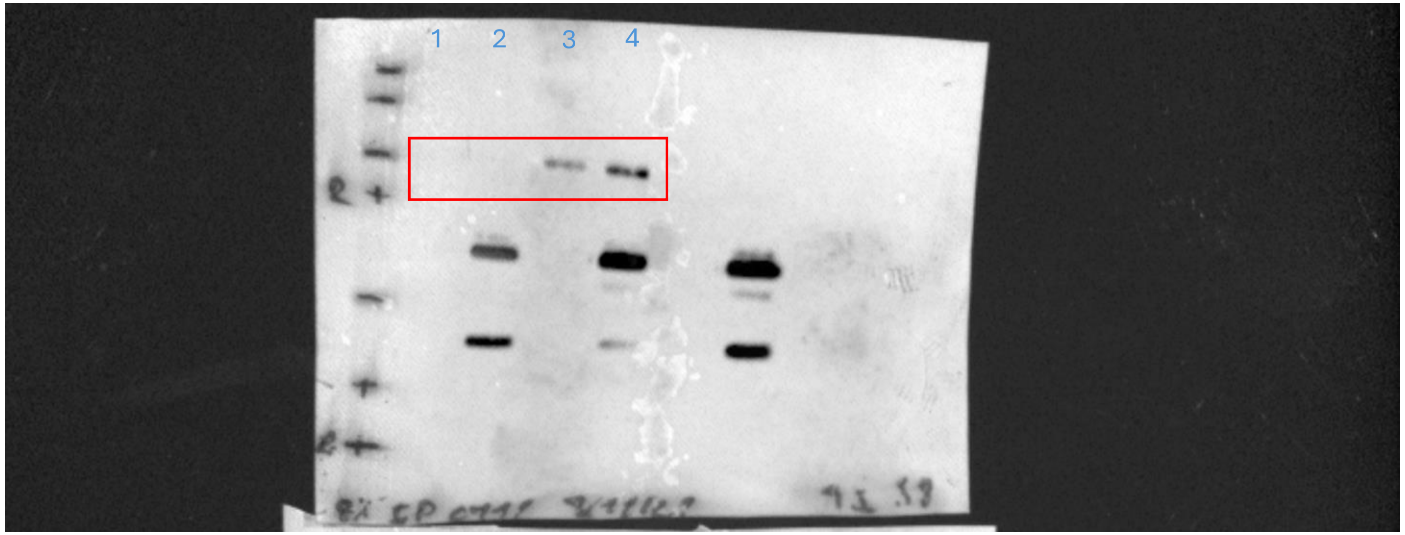

Supplement: Figure 8—source data 2. [file elife-94347-fig8-data2.zip › Figure 8-source data 2.pdf]
