# Supplementary material for: Zika virus remodels and hijacks IGF2BP2 ribonucleoprotein complex to promote viral replication organelle biogenesis
Source: eLife. 2024 Nov 20;13:RP94347. doi: 10.7554/eLife.94347 (PMC11578589; doi:10.7554/eLife.94347)

Figure 8-fig supplement 3A

Anti-IGF2BP2

Cell extracts

1- Mock IGF2BP2-HA (-) Rnase A(-)
2- Mock IGF2BP2-HA (-) RNase A (+)
3- Mock IGF2BP2-HA (+) Rnase A(-)
4- Mock IGF2BP2-HA (+) Rnase A(+)
5- ZIKV IGF2BP2-HA (-) Rnase A(-)
6- ZIKV IGF2BP2-HA (-) RNase A (+)
7- ZIKV IGF2BP2-HA (+) Rnase A(-)
8- ZIKV IGF2BP2-HA (+) Rnase A(+)

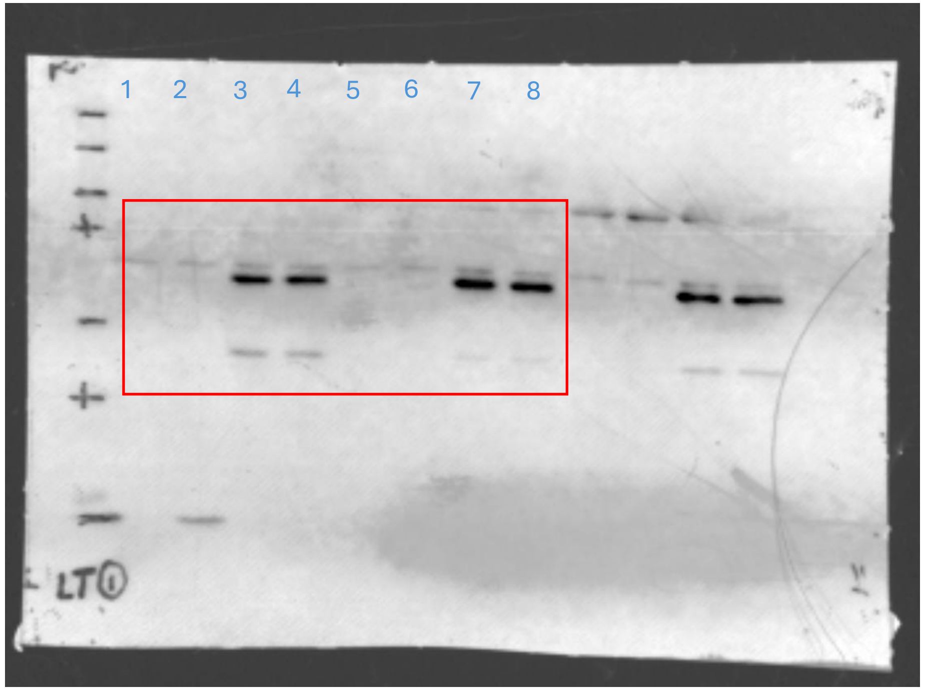

Anti-HA

Cell extracts

1- Mock IGF2BP2-HA (-) Rnase A(-)
2- Mock IGF2BP2-HA (-) RNase A (+)
3- Mock IGF2BP2-HA (+) Rnase A(-)
4- Mock IGF2BP2-HA (+) Rnase A(+)
5- ZIKV IGF2BP2-HA (-) Rnase A(-)
6- ZIKV IGF2BP2-HA (-) RNase A (+)
7- ZIKV IGF2BP2-HA (+) Rnase A(-)
8- ZIKV IGF2BP2-HA (+) Rnase A(+)

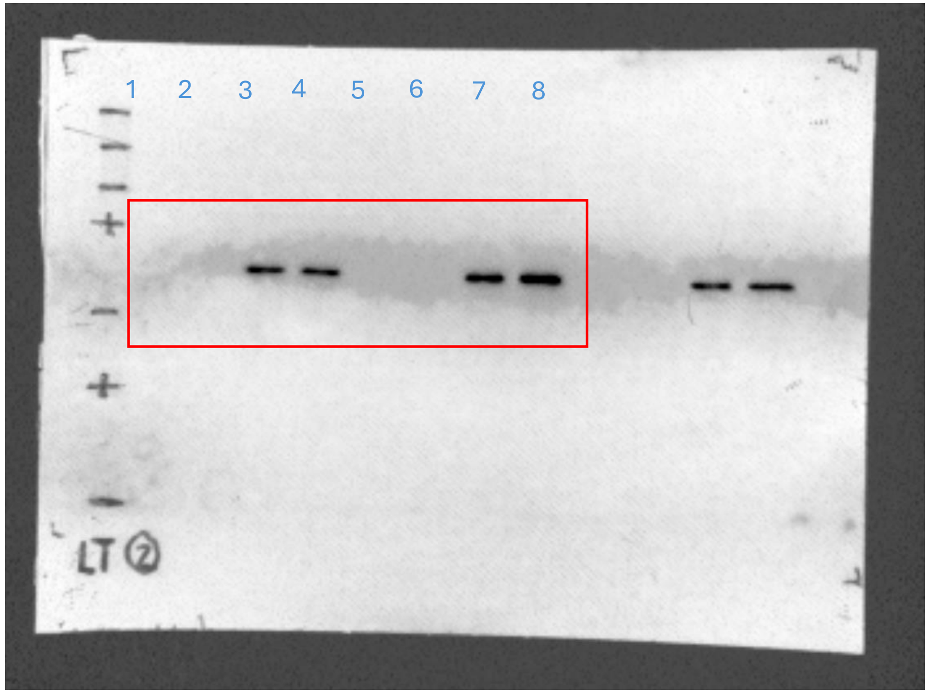

Anti-ZIKV NS5

Cell extracts

1- Mock IGF2BP2-HA (-) Rnase A(-)
2- Mock IGF2BP2-HA (-) RNase A (+)
3- Mock IGF2BP2-HA (+) Rnase A(-)
4- Mock IGF2BP2-HA (+) Rnase A(+)
5- ZIKV IGF2BP2-HA (-) Rnase A(-)
6- ZIKV IGF2BP2-HA (-) RNase A (+)
7- ZIKV IGF2BP2-HA (+) Rnase A(-)
8- ZIKV IGF2BP2-HA (+) Rnase A(+)

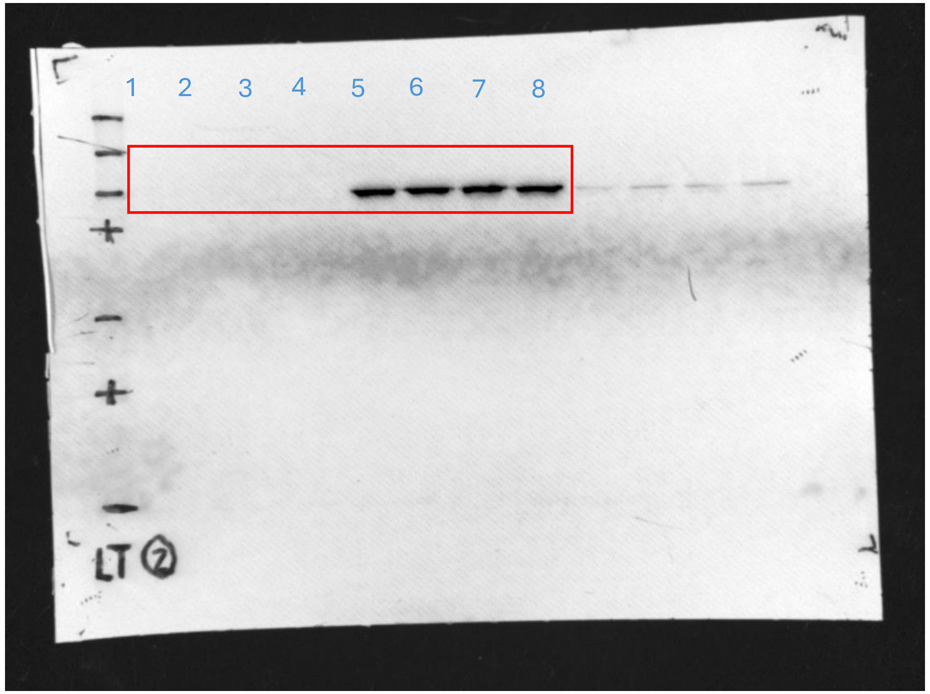

Anti-ATL2

Cell extracts

1- Mock IGF2BP2-HA (-) Rnase A(-)
2- Mock IGF2BP2-HA (-) RNase A (+)
3- Mock IGF2BP2-HA (+) Rnase A(-)
4- Mock IGF2BP2-HA (+) Rnase A(+)
5- ZIKV IGF2BP2-HA (-) Rnase A(-)
6- ZIKV IGF2BP2-HA (-) RNase A (+)
7- ZIKV IGF2BP2-HA (+) Rnase A(-)
8- ZIKV IGF2BP2-HA (+) Rnase A(+)

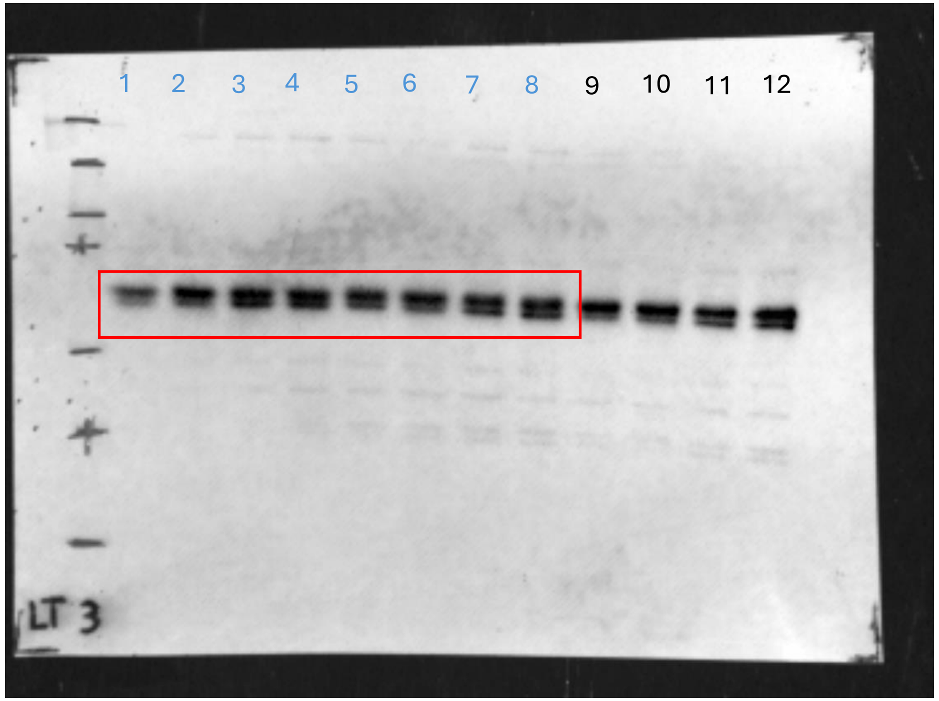

Anti-IGF2BP1

Cell extracts

1- Mock IGF2BP2-HA (-) Rnase A(-)
2- Mock IGF2BP2-HA (-) RNase A (+)
3- Mock IGF2BP2-HA (+) Rnase A(-)
4- Mock IGF2BP2-HA (+) Rnase A(+)
5- ZIKV IGF2BP2-HA (-) Rnase A(-)
6- ZIKV IGF2BP2-HA (-) RNase A (+)
7- ZIKV IGF2BP2-HA (+) Rnase A(-)
8- ZIKV IGF2BP2-HA (+) Rnase A(+)

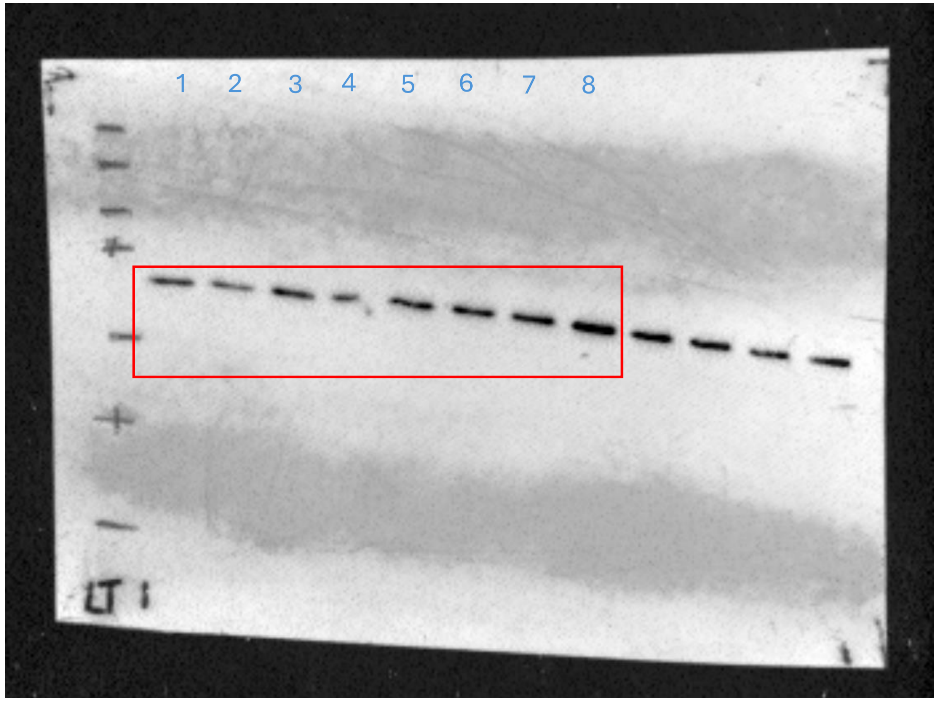

Anti-IGF2BP3

Cell extracts

1- Mock IGF2BP2-HA (-) Rnase A(-)
2- Mock IGF2BP2-HA (-) RNase A (+)
3- Mock IGF2BP2-HA (+) Rnase A(-)
4- Mock IGF2BP2-HA (+) Rnase A(+)
5- ZIKV IGF2BP2-HA (-) Rnase A(-)
6- ZIKV IGF2BP2-HA (-) RNase A (+)
7- ZIKV IGF2BP2-HA (+) Rnase A(-)
8- ZIKV IGF2BP2-HA (+) Rnase A(+)

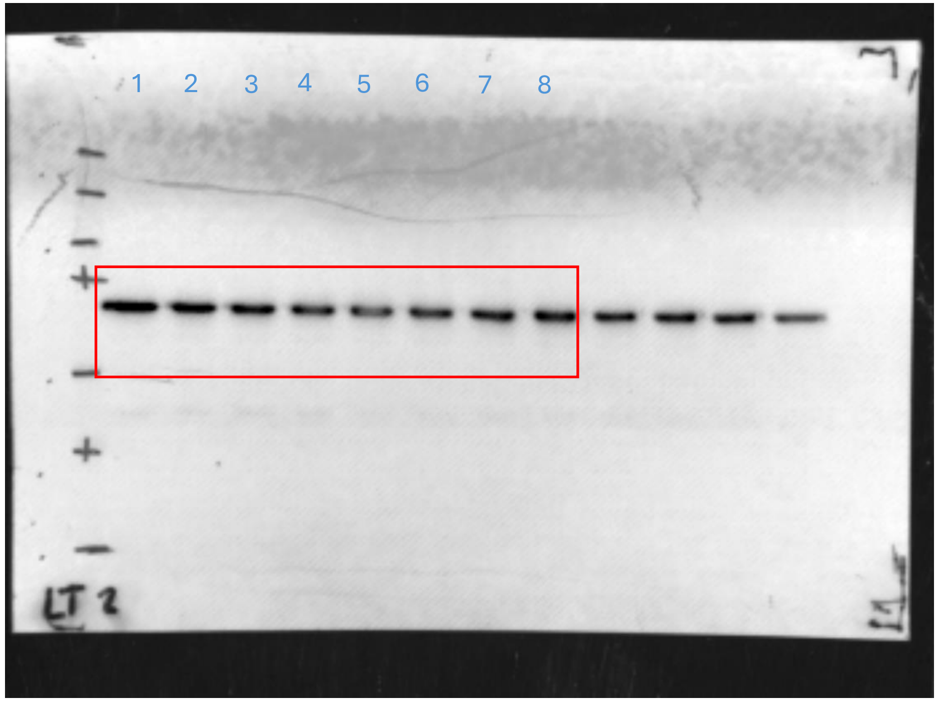

Anti-actin

Cell extracts

1- Mock IGF2BP2-HA (-) Rnase A(-)
2- Mock IGF2BP2-HA (-) RNase A (+)
3- Mock IGF2BP2-HA (+) Rnase A(-)
4- Mock IGF2BP2-HA (+) Rnase A(+)
5- ZIKV IGF2BP2-HA (-) Rnase A(-)
6- ZIKV IGF2BP2-HA (-) RNase A (+)
7- ZIKV IGF2BP2-HA (+) Rnase A(-)
8- ZIKV IGF2BP2-HA (+) Rnase A(+)

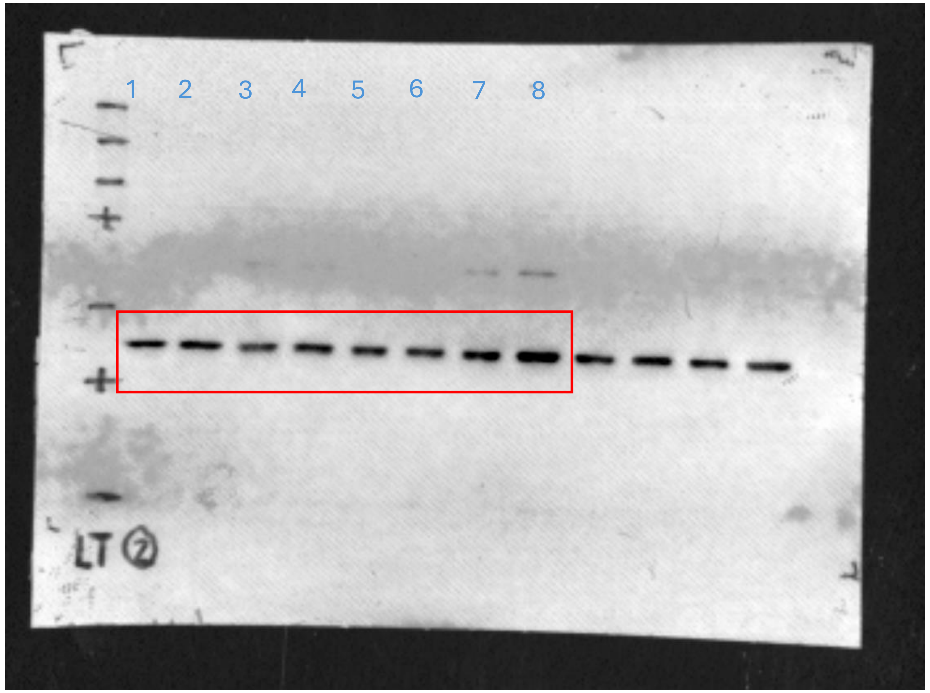

Anti-IGF2BP2

IP anti-HA

1- Mock IGF2BP2-HA (-) Rnase A(-)
2- Mock IGF2BP2-HA (-) RNase A (+)
3- Mock IGF2BP2-HA (+) Rnase A(-)
4- Mock IGF2BP2-HA (+) Rnase A(+)
5- ZIKV IGF2BP2-HA (-) Rnase A(-)
6- ZIKV IGF2BP2-HA (-) RNase A (+)
7- ZIKV IGF2BP2-HA (+) Rnase A(-)
8- ZIKV IGF2BP2-HA (+) Rnase A(+)

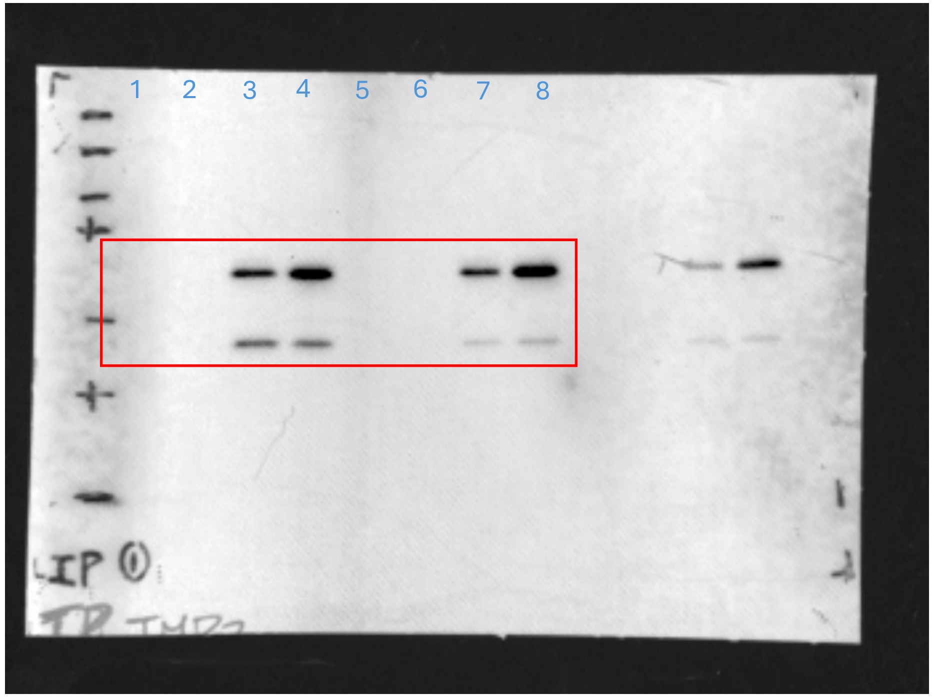

Anti-HA

IP anti-HA

1- Mock IGF2BP2-HA (-) Rnase A(-)
2- Mock IGF2BP2-HA (-) RNase A (+)
3- Mock IGF2BP2-HA (+) Rnase A(-)
4- Mock IGF2BP2-HA (+) Rnase A(+)
5- ZIKV IGF2BP2-HA (-) Rnase A(-)
6- ZIKV IGF2BP2-HA (-) RNase A (+)
7- ZIKV IGF2BP2-HA (+) Rnase A(-)
8- ZIKV IGF2BP2-HA (+) Rnase A(+)

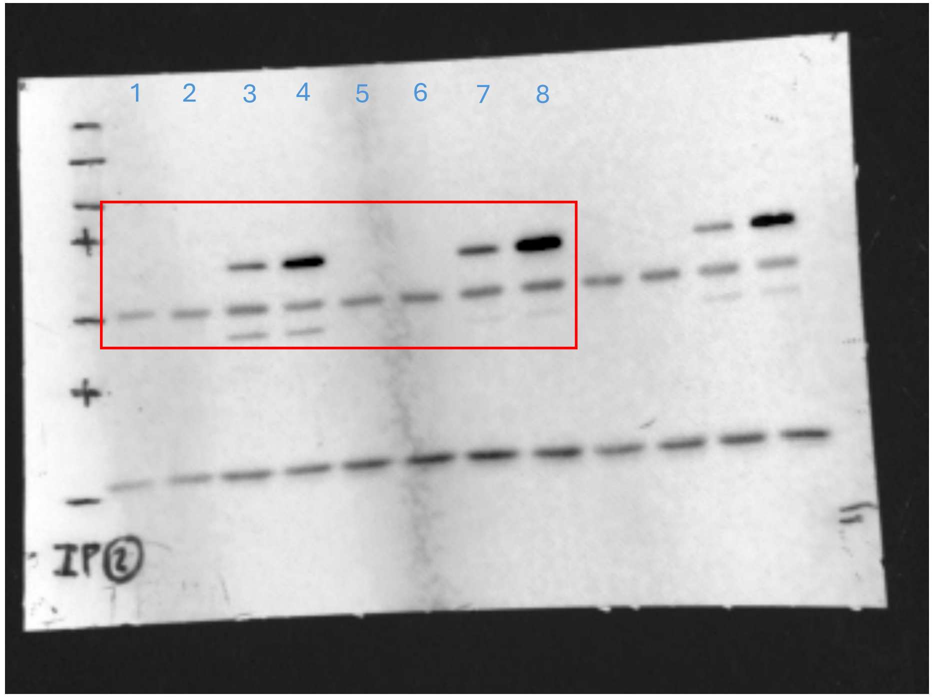

Anti-ZIKV NS5

IP anti-HA

1- Mock IGF2BP2-HA (-) Rnase A(-)
2- Mock IGF2BP2-HA (-) RNase A (+)
3- Mock IGF2BP2-HA (+) Rnase A(-)
4- Mock IGF2BP2-HA (+) Rnase A(+)
5- ZIKV IGF2BP2-HA (-) Rnase A(-)
6- ZIKV IGF2BP2-HA (-) RNase A (+)
7- ZIKV IGF2BP2-HA (+) Rnase A(-)
8- ZIKV IGF2BP2-HA (+) Rnase A(+)

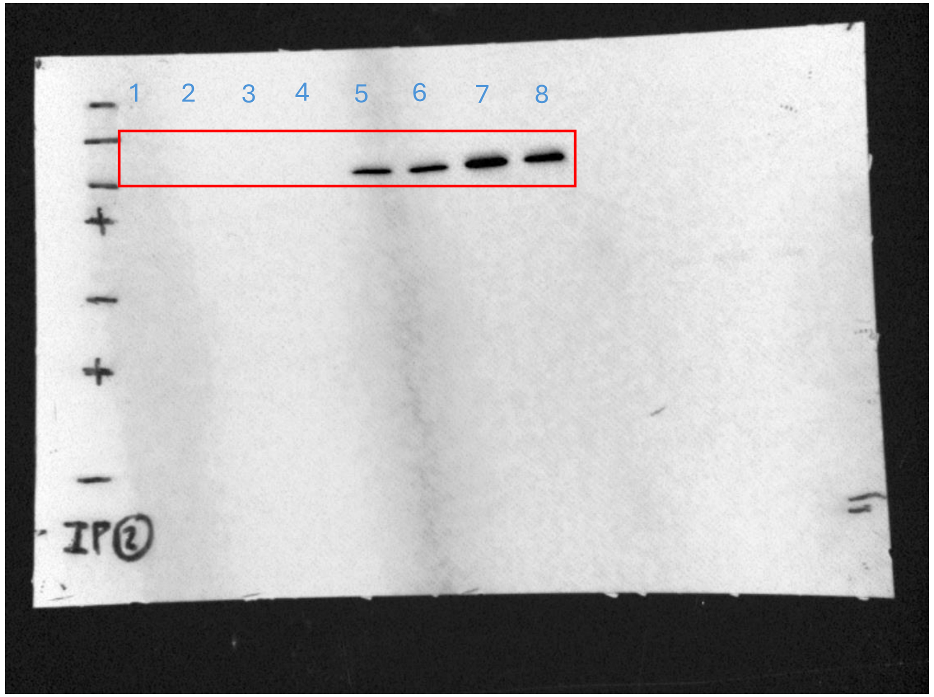

Anti-ATL2

IP anti-HA

1- Mock IGF2BP2-HA (-) Rnase A(-)
2- Mock IGF2BP2-HA (-) RNase A (+)
3- Mock IGF2BP2-HA (+) Rnase A(-)
4- Mock IGF2BP2-HA (+) Rnase A(+)
5- ZIKV IGF2BP2-HA (-) Rnase A(-)
6- ZIKV IGF2BP2-HA (-) RNase A (+)
7- ZIKV IGF2BP2-HA (+) Rnase A(-)
8- ZIKV IGF2BP2-HA (+) Rnase A(+)

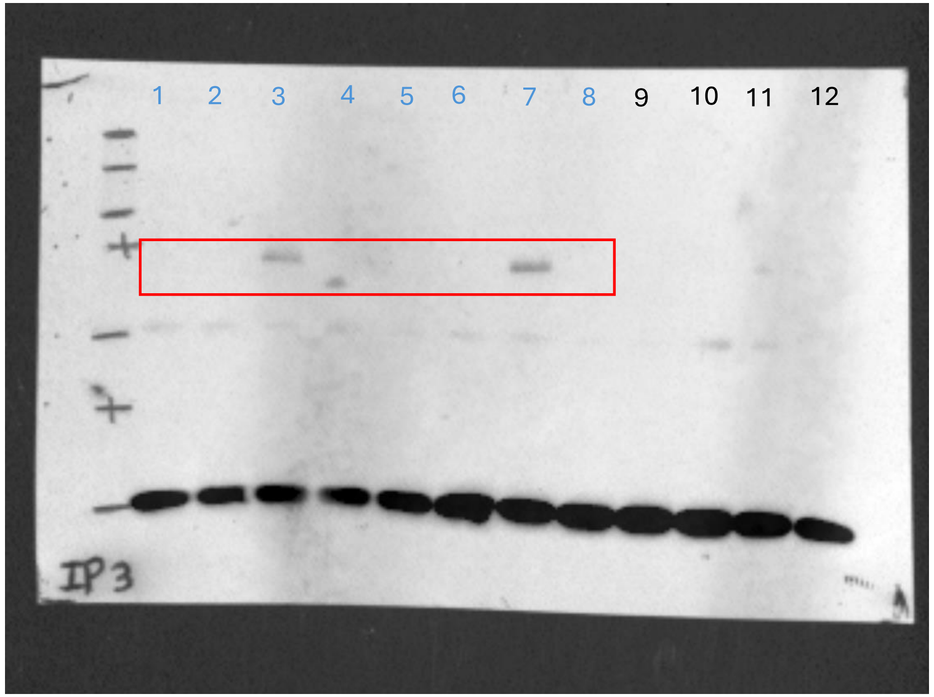

Anti-IGF2BP1

IP anti-HA

1- Mock IGF2BP2-HA (-) Rnase A(-)
2- Mock IGF2BP2-HA (-) RNase A (+)
3- Mock IGF2BP2-HA (+) Rnase A(-)
4- Mock IGF2BP2-HA (+) Rnase A(+)
5- ZIKV IGF2BP2-HA (-) Rnase A(-)
6- ZIKV IGF2BP2-HA (-) RNase A (+)
7- ZIKV IGF2BP2-HA (+) Rnase A(-)
8- ZIKV IGF2BP2-HA (+) Rnase A(+)

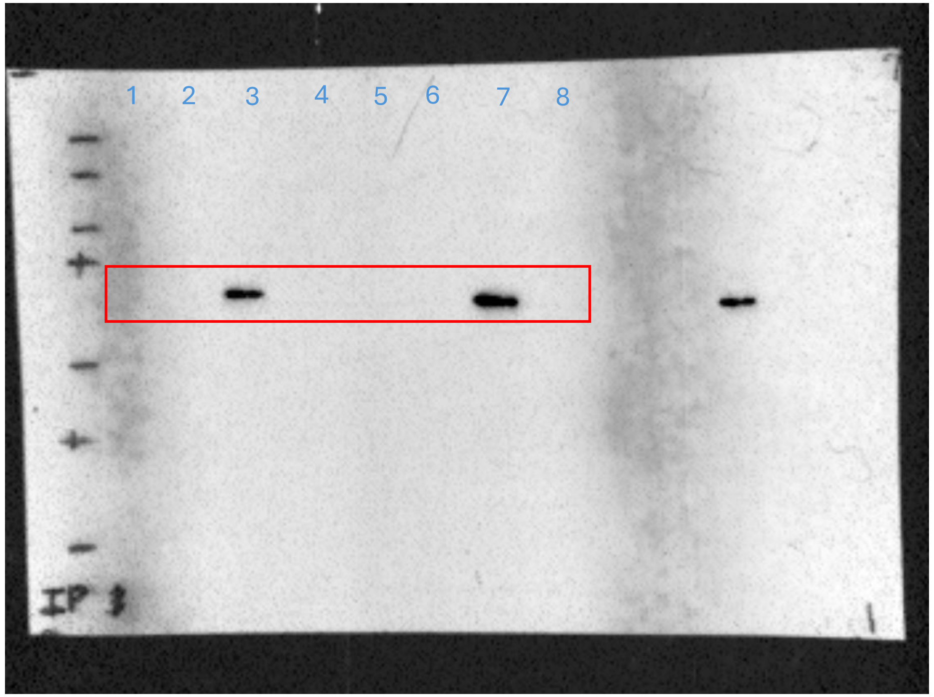

Anti-IGF2BP3

IP anti-HA

1- Mock IGF2BP2-HA (-) Rnase A(-)
2- Mock IGF2BP2-HA (-) RNase A (+)
3- Mock IGF2BP2-HA (+) Rnase A(-)
4- Mock IGF2BP2-HA (+) Rnase A(+)
5- ZIKV IGF2BP2-HA (-) Rnase A(-)
6- ZIKV IGF2BP2-HA (-) RNase A (+)
7- ZIKV IGF2BP2-HA (+) Rnase A(-)
8- ZIKV IGF2BP2-HA (+) Rnase A(+)

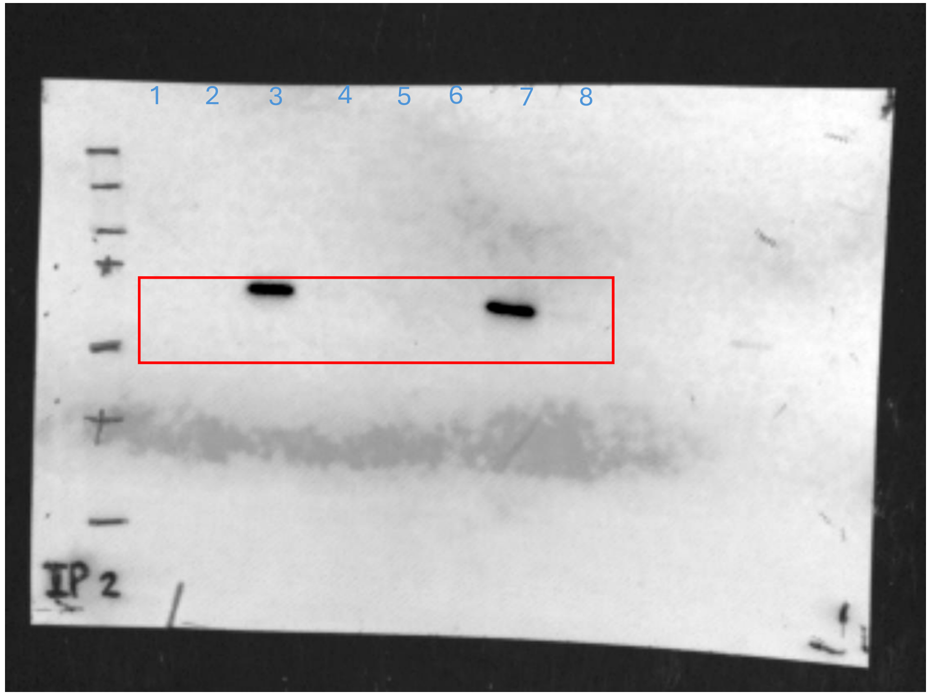

Supplement: Figure 8—figure supplement 3—source data 2. [file elife-94347-fig8-figsupp3-data2.zip › Figure 8-figure supplement 3-source data 2.pdf]
