# Supplementary material for: Zika virus remodels and hijacks IGF2BP2 ribonucleoprotein complex to promote viral replication organelle biogenesis
Source: eLife. 2024 Nov 20;13:RP94347. doi: 10.7554/eLife.94347 (PMC11578589; doi:10.7554/eLife.94347)

Figure 9-figure supplement 2A

Anti-HA

Cell extracts

1- Mock IGF2BP2-HA (-) VCP-HA(-)
2- Mock IGF2BP2-HA (+) VCP-HA(-)
3- Mock IGF2BP2-HA (-) VCP-HA(+)
4- ZIKV IGF2BP2-HA (-) VCP-HA(-)
5- ZIKV IGF2BP2-HA (+) VCP-HA(-)
6- ZIKV IGF2BP2-HA (-) VCP-HA (+)
7- DENV IGF2BP2-HA (-) VCP-HA(-)
8- DENV IGF2BP2-HA (+) VCP-HA (-)
9- DENV IGF2BP2-HA (-) VCP-HA(+)

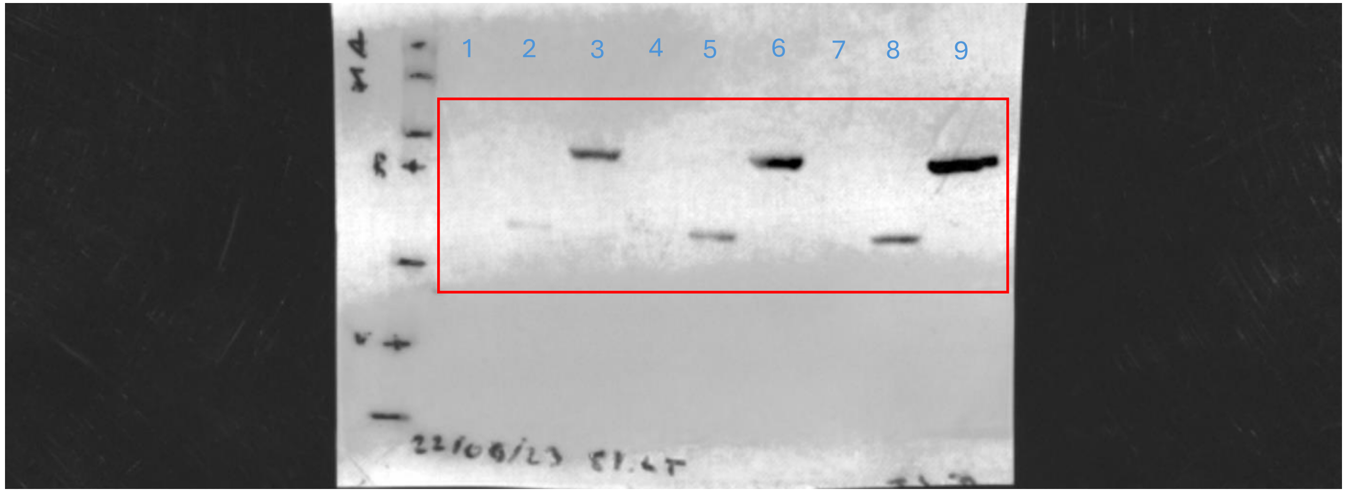

Anti-VCP

Cell extracts

1- Mock IGF2BP2-HA (-) VCP-HA(-)
2- Mock IGF2BP2-HA (+) VCP-HA(-)
3- Mock IGF2BP2-HA (-) VCP-HA(+)
4- ZIKV IGF2BP2-HA (-) VCP-HA(-)
5- ZIKV IGF2BP2-HA (+) VCP-HA(-)
6- ZIKV IGF2BP2-HA (-) VCP-HA (+)
7- DENV IGF2BP2-HA (-) VCP-HA(-)
8- DENV IGF2BP2-HA (+) VCP-HA (-)
9- DENV IGF2BP2-HA (-) VCP-HA(+)

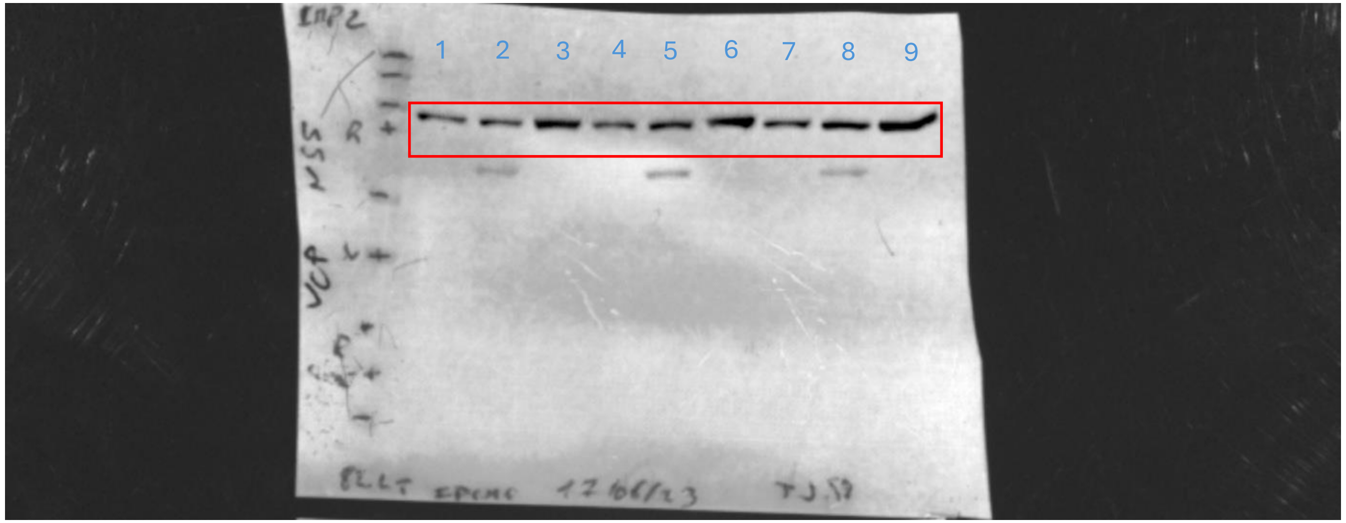

Anti-IGF2BP2

Cell extracts

1- Mock IGF2BP2-HA (-) VCP-HA(-)
2- Mock IGF2BP2-HA (+) VCP-HA(-)
3- Mock IGF2BP2-HA (-) VCP-HA(+)
4- ZIKV IGF2BP2-HA (-) VCP-HA(-)
5- ZIKV IGF2BP2-HA (+) VCP-HA(-)
6- ZIKV IGF2BP2-HA (-) VCP-HA (+)
7- DENV IGF2BP2-HA (-) VCP-HA(-)
8- DENV IGF2BP2-HA (+) VCP-HA (-)
9- DENV IGF2BP2-HA (-) VCP-HA(+)

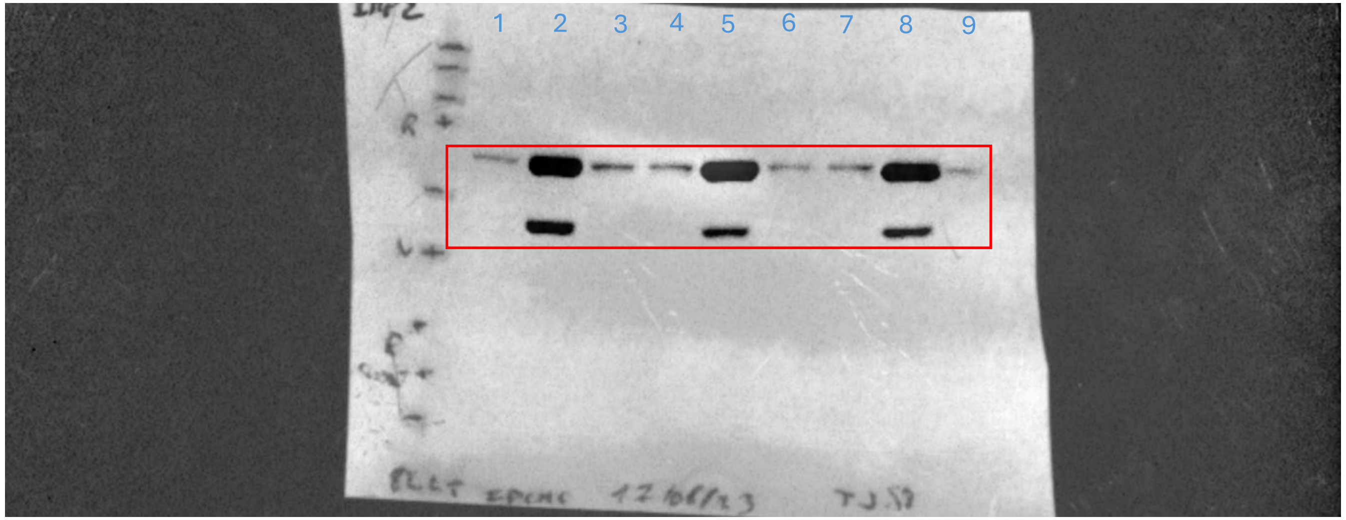

Anti-NS5

Cell extracts

1- Mock IGF2BP2-HA (-) VCP-HA(-)
2- Mock IGF2BP2-HA (+) VCP-HA(-)
3- Mock IGF2BP2-HA (-) VCP-HA(+)
4- ZIKV IGF2BP2-HA (-) VCP-HA(-)
5- ZIKV IGF2BP2-HA (+) VCP-HA(-)
6- ZIKV IGF2BP2-HA (-) VCP-HA (+)
7- DENV IGF2BP2-HA (-) VCP-HA(-)
8- DENV IGF2BP2-HA (+) VCP-HA (-)
9- DENV IGF2BP2-HA (-) VCP-HA(+)

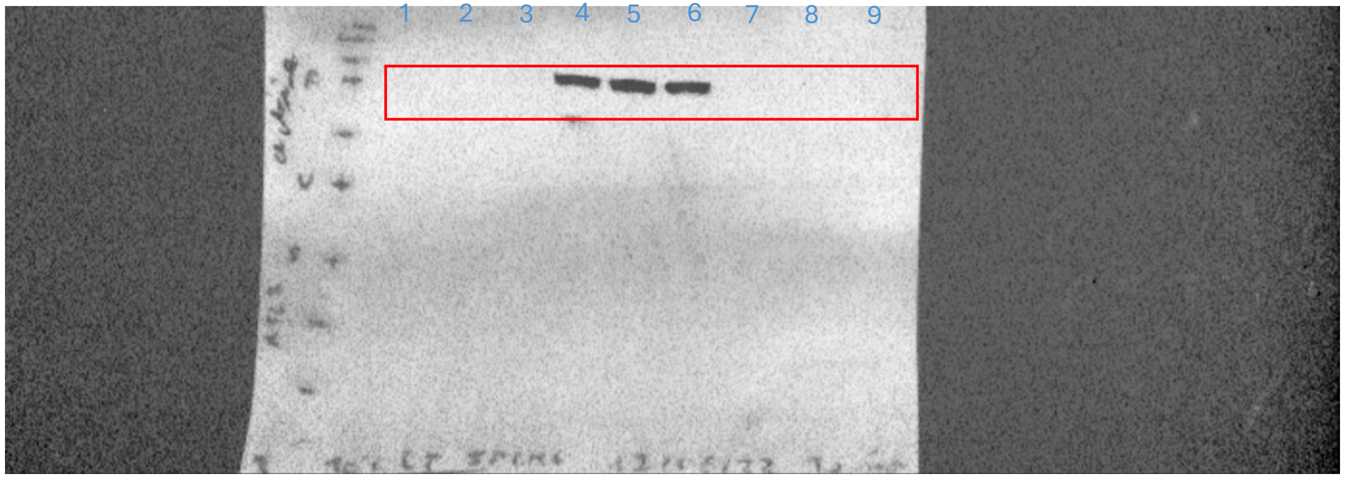

Anti-ATL2

Cell extracts

1- Mock IGF2BP2-HA (-) VCP-HA(-)
2- Mock IGF2BP2-HA (+) VCP-HA(-)
3- Mock IGF2BP2-HA (-) VCP-HA(+)
4- ZIKV IGF2BP2-HA (-) VCP-HA(-)
5- ZIKV IGF2BP2-HA (+) VCP-HA(-)
6- ZIKV IGF2BP2-HA (-) VCP-HA (+)
7- DENV IGF2BP2-HA (-) VCP-HA(-)
8- DENV IGF2BP2-HA (+) VCP-HA (-)
9- DENV IGF2BP2-HA (-) VCP-HA(+)

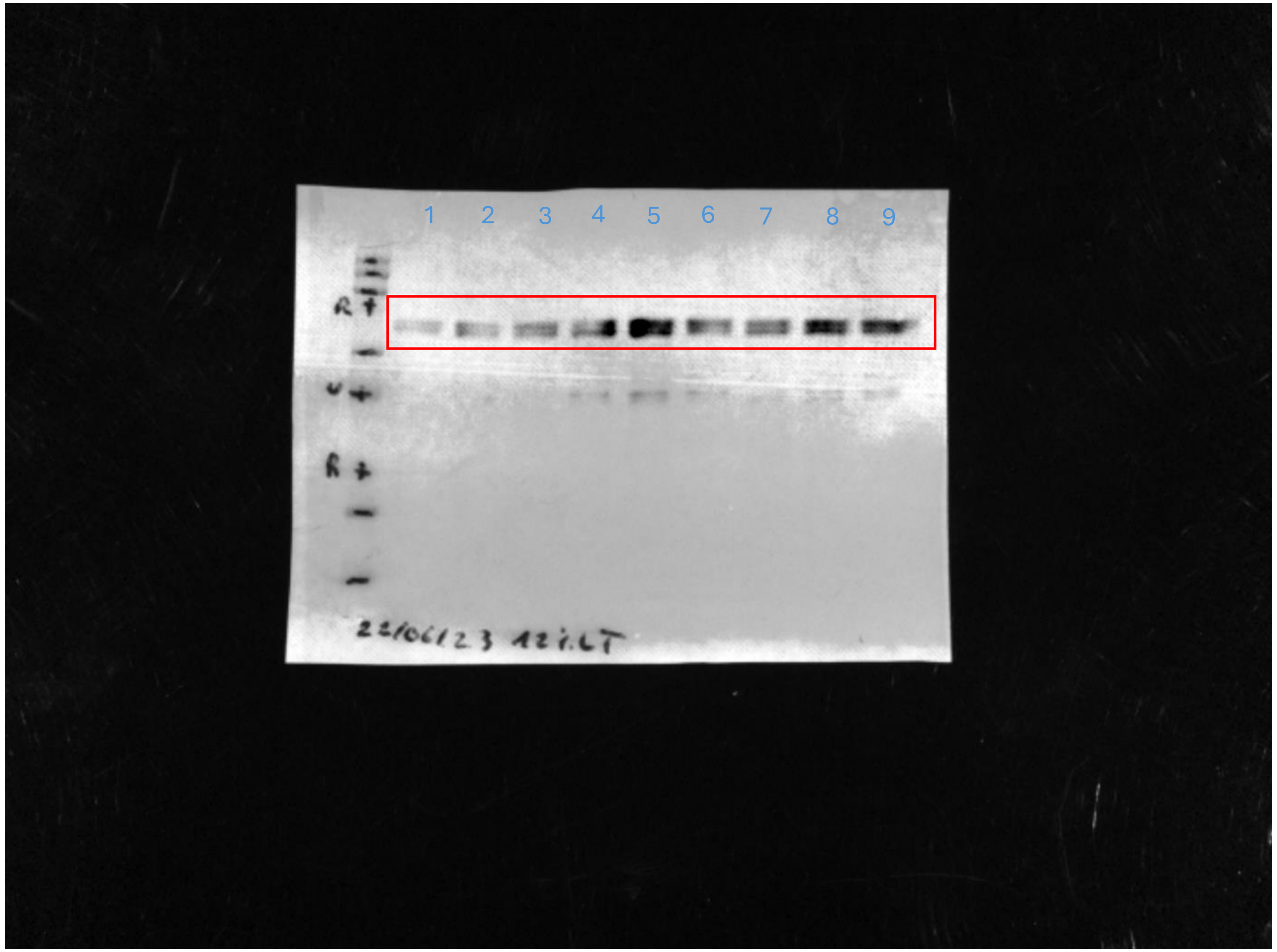

Anti-IGF2BP1

Cell extracts

1- Mock IGF2BP2-HA (-) VCP-HA(-)
2- Mock IGF2BP2-HA (+) VCP-HA(-)
3- Mock IGF2BP2-HA (-) VCP-HA(+)
4- ZIKV IGF2BP2-HA (-) VCP-HA(-)
5- ZIKV IGF2BP2-HA (+) VCP-HA(-)
6- ZIKV IGF2BP2-HA (-) VCP-HA (+)
7- DENV IGF2BP2-HA (-) VCP-HA(-)
8- DENV IGF2BP2-HA (+) VCP-HA (-)
9- DENV IGF2BP2-HA (-) VCP-HA(+)

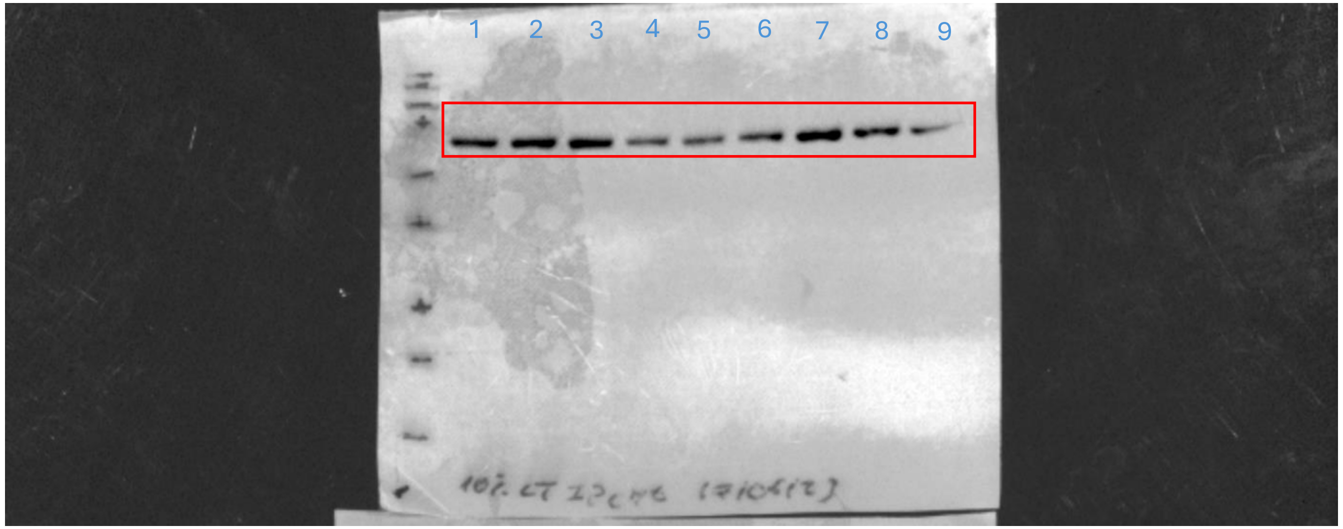

Anti-IGF2BP3

Cell extracts

1- Mock IGF2BP2-HA (-) VCP-HA(-)
2- Mock IGF2BP2-HA (+) VCP-HA(-)
3- Mock IGF2BP2-HA (-) VCP-HA(+)
4- ZIKV IGF2BP2-HA (-) VCP-HA(-)
5- ZIKV IGF2BP2-HA (+) VCP-HA(-)
6- ZIKV IGF2BP2-HA (-) VCP-HA (+)
7- DENV IGF2BP2-HA (-) VCP-HA(-)
8- DENV IGF2BP2-HA (+) VCP-HA (-)
9- DENV IGF2BP2-HA (-) VCP-HA(+)

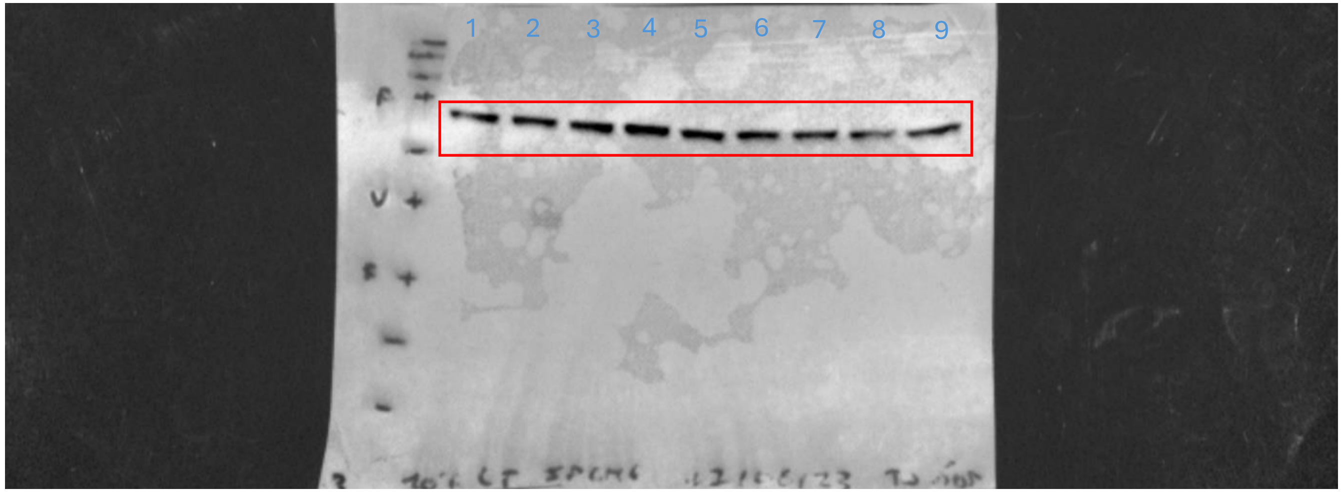

Anti-YBX1

Cell extracts

1- Mock IGF2BP2-HA (-) VCP-HA(-)
2- Mock IGF2BP2-HA (+) VCP-HA(-)
3- Mock IGF2BP2-HA (-) VCP-HA(+)
4- ZIKV IGF2BP2-HA (-) VCP-HA(-)
5- ZIKV IGF2BP2-HA (+) VCP-HA(-)
6- ZIKV IGF2BP2-HA (-) VCP-HA (+)
7- DENV IGF2BP2-HA (-) VCP-HA(-)
8- DENV IGF2BP2-HA (+) VCP-HA (-)
9- DENV IGF2BP2-HA (-) VCP-HA(+)

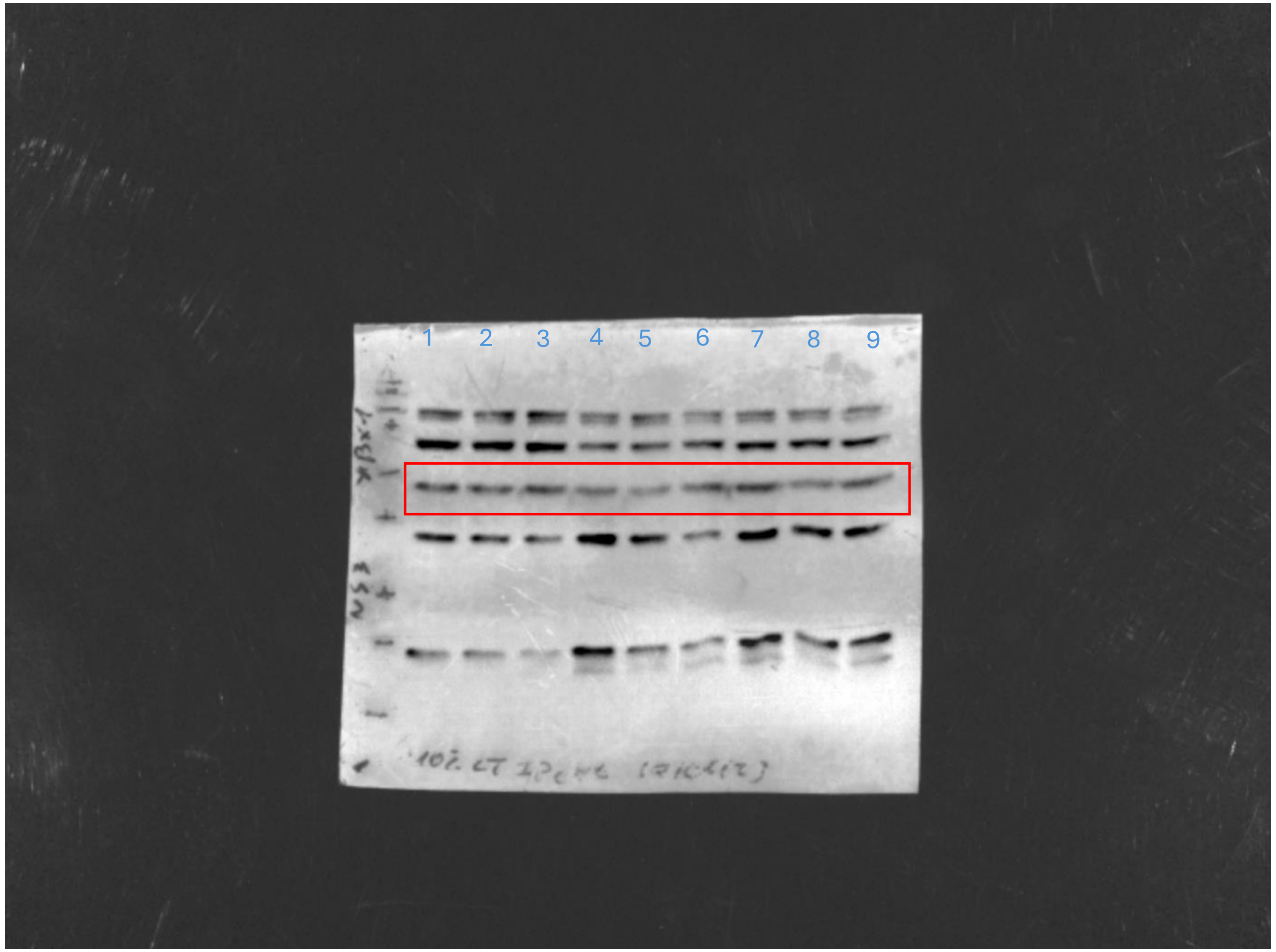

Anti-actin

Cell extracts

1- Mock IGF2BP2-HA (-) VCP-HA(-)
2- Mock IGF2BP2-HA (+) VCP-HA(-)
3- Mock IGF2BP2-HA (-) VCP-HA(+)
4- ZIKV IGF2BP2-HA (-) VCP-HA(-)
5- ZIKV IGF2BP2-HA (+) VCP-HA(-)
6- ZIKV IGF2BP2-HA (-) VCP-HA (+)
7- DENV IGF2BP2-HA (-) VCP-HA(-)
8- DENV IGF2BP2-HA (+) VCP-HA (-)
9- DENV IGF2BP2-HA (-) VCP-HA(+)

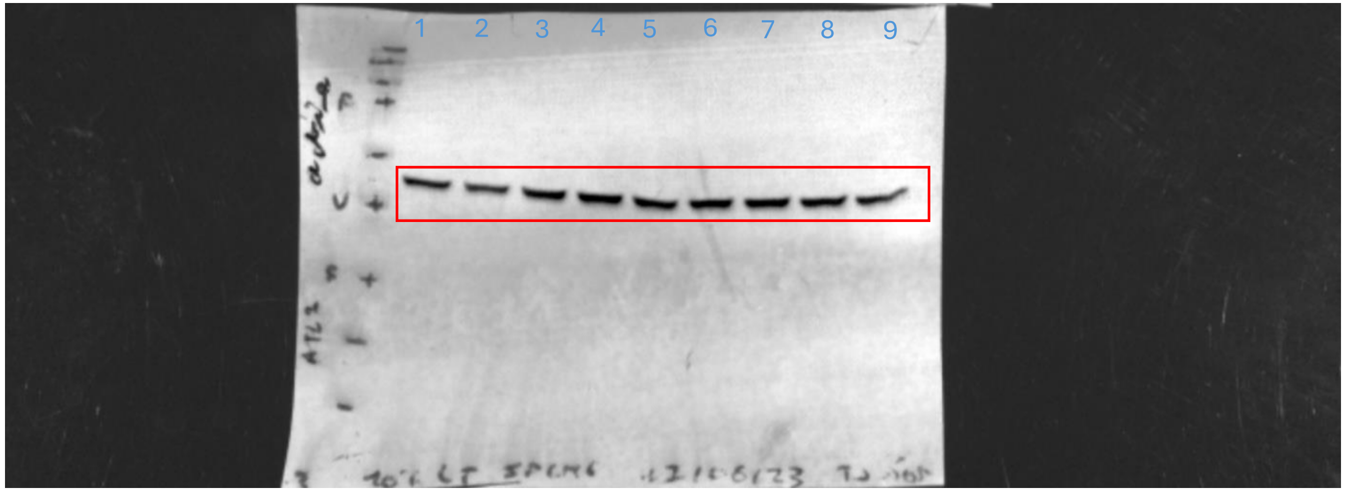

Anti-HA

IP anti-HA

1- Mock IGF2BP2-HA (-) VCP-HA(-)
2- Mock IGF2BP2-HA (+) VCP-HA(-)
3- Mock IGF2BP2-HA (-) VCP-HA(+)
4- ZIKV IGF2BP2-HA (-) VCP-HA(-)
5- ZIKV IGF2BP2-HA (+) VCP-HA(-)
6- ZIKV IGF2BP2-HA (-) VCP-HA (+)
7- DENV IGF2BP2-HA (-) VCP-HA(-)
8- DENV IGF2BP2-HA (+) VCP-HA (-)
9- DENV IGF2BP2-HA (-) VCP-HA(+)

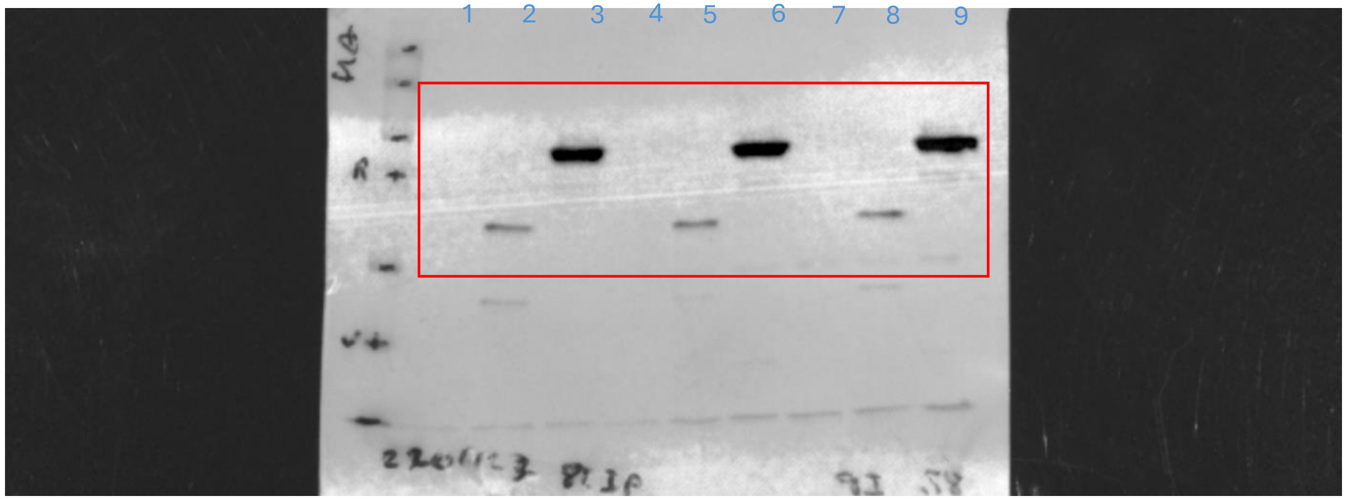

Anti-VCP

IP anti-HA

1- Mock IGF2BP2-HA (-) VCP-HA(-)
2- Mock IGF2BP2-HA (+) VCP-HA(-)
3- Mock IGF2BP2-HA (-) VCP-HA(+)
4- ZIKV IGF2BP2-HA (-) VCP-HA(-)
5- ZIKV IGF2BP2-HA (+) VCP-HA(-)
6- ZIKV IGF2BP2-HA (-) VCP-HA (+)
7- DENV IGF2BP2-HA (-) VCP-HA(-)
8- DENV IGF2BP2-HA (+) VCP-HA (-)
9- DENV IGF2BP2-HA (-) VCP-HA(+)

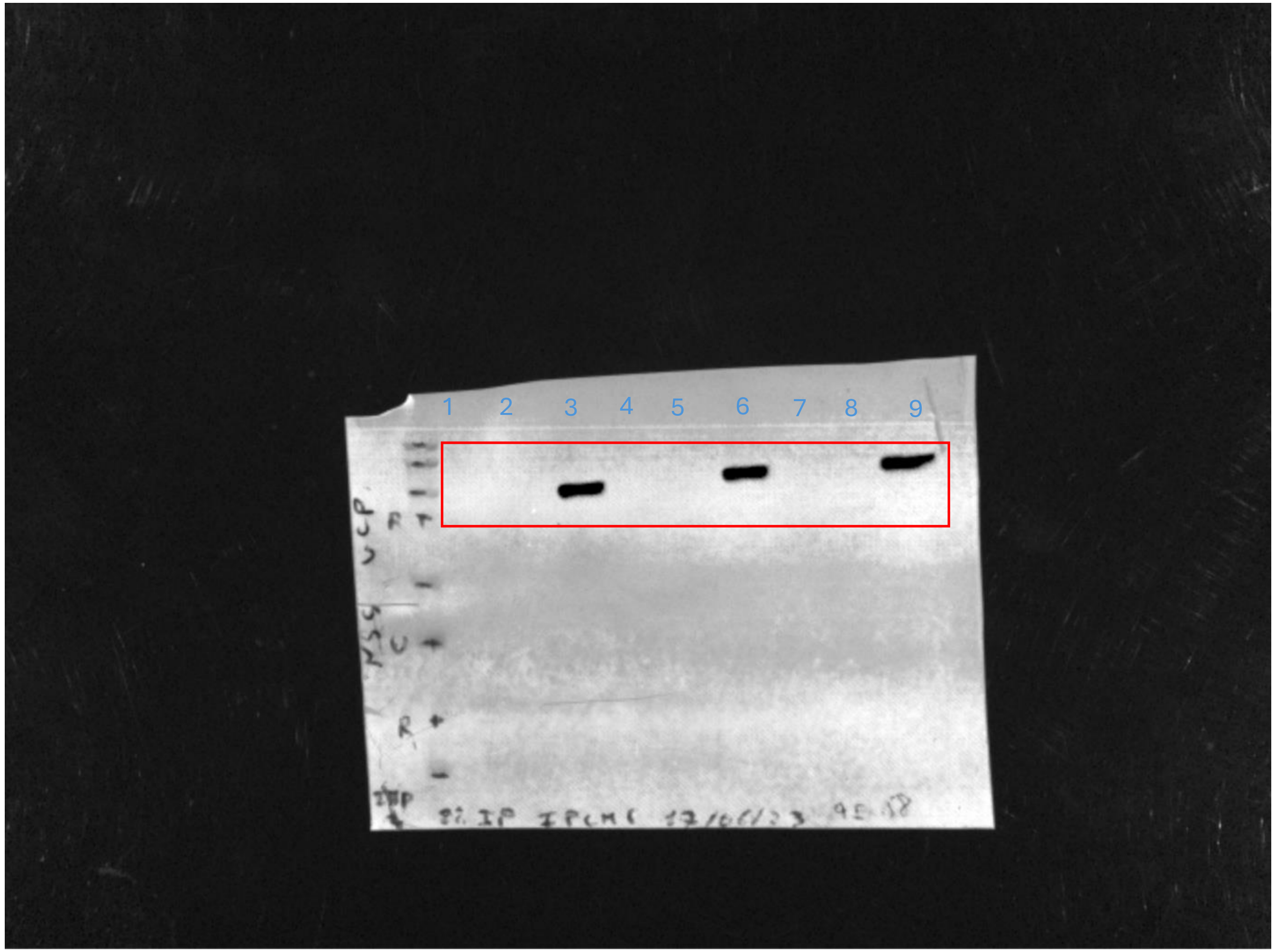

Anti-IGF2BP2

IP anti-HA

1- Mock IGF2BP2-HA (-) VCP-HA(-)
2- Mock IGF2BP2-HA (+) VCP-HA(-)
3- Mock IGF2BP2-HA (-) VCP-HA(+)
4- ZIKV IGF2BP2-HA (-) VCP-HA(-)
5- ZIKV IGF2BP2-HA (+) VCP-HA(-)
6- ZIKV IGF2BP2-HA (-) VCP-HA (+)
7- DENV IGF2BP2-HA (-) VCP-HA(-)
8- DENV IGF2BP2-HA (+) VCP-HA (-)
9- DENV IGF2BP2-HA (-) VCP-HA(+)

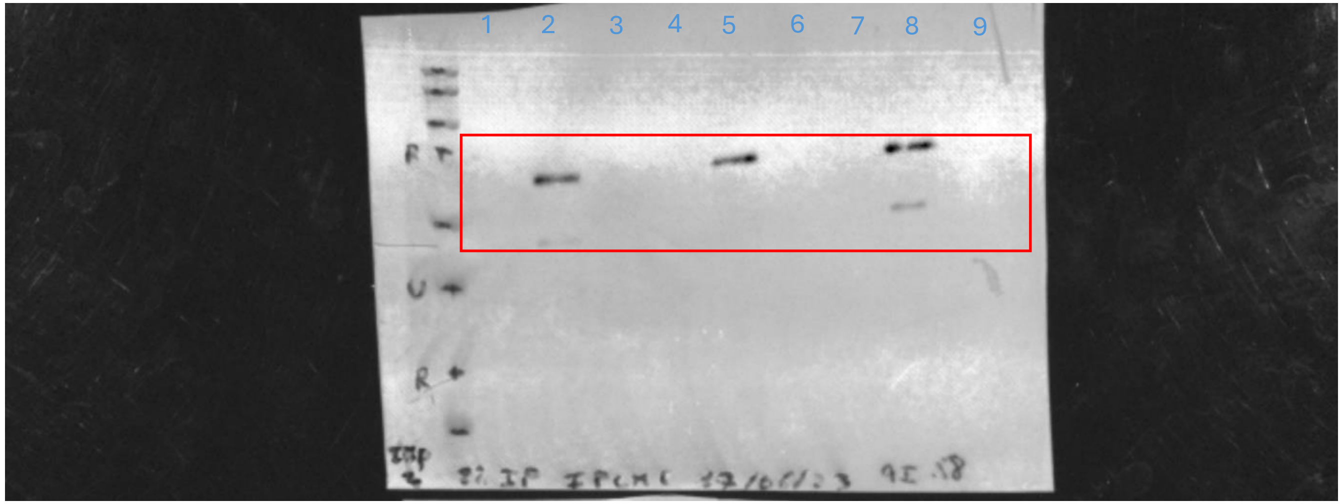

Anti-NS5

IP anti-HA

1- Mock IGF2BP2-HA (-) VCP-HA(-)
2- Mock IGF2BP2-HA (+) VCP-HA(-)
3- Mock IGF2BP2-HA (-) VCP-HA(+)
4- ZIKV IGF2BP2-HA (-) VCP-HA(-)
5- ZIKV IGF2BP2-HA (+) VCP-HA(-)
6- ZIKV IGF2BP2-HA (-) VCP-HA (+)
7- DENV IGF2BP2-HA (-) VCP-HA(-)
8- DENV IGF2BP2-HA (+) VCP-HA (-)
9- DENV IGF2BP2-HA (-) VCP-HA(+)

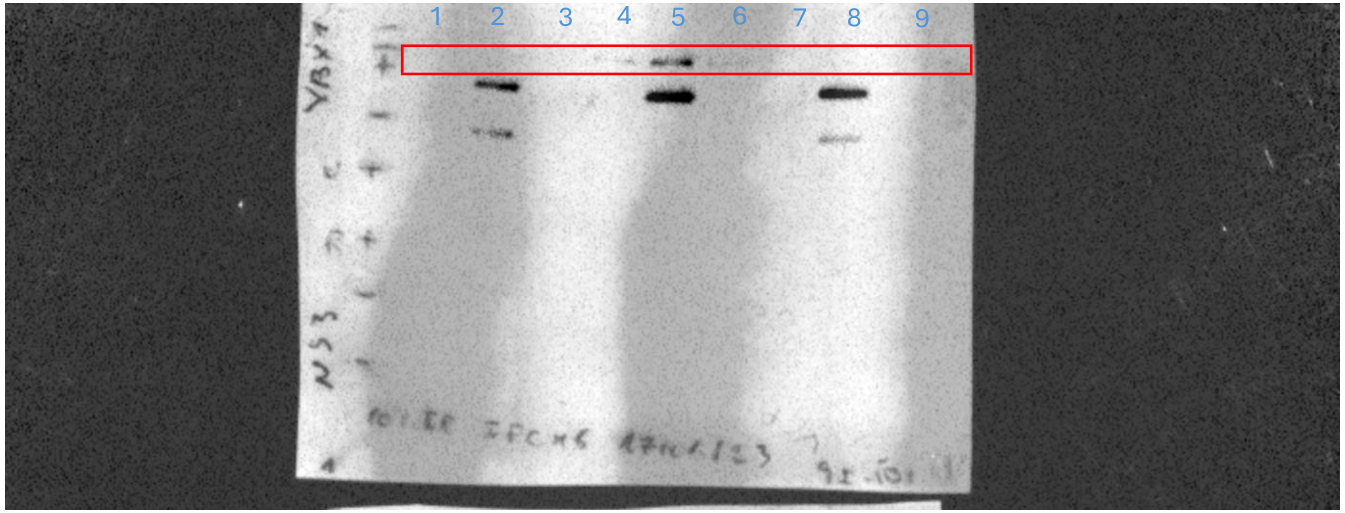

Anti-ATL2

IP anti-HA

1- Mock IGF2BP2-HA (-) VCP-HA(-)
2- Mock IGF2BP2-HA (+) VCP-HA(-)
3- Mock IGF2BP2-HA (-) VCP-HA(+)
4- ZIKV IGF2BP2-HA (-) VCP-HA(-)
5- ZIKV IGF2BP2-HA (+) VCP-HA(-)
6- ZIKV IGF2BP2-HA (-) VCP-HA (+)
7- DENV IGF2BP2-HA (-) VCP-HA(-)
8- DENV IGF2BP2-HA (+) VCP-HA (-)
9- DENV IGF2BP2-HA (-) VCP-HA(+)

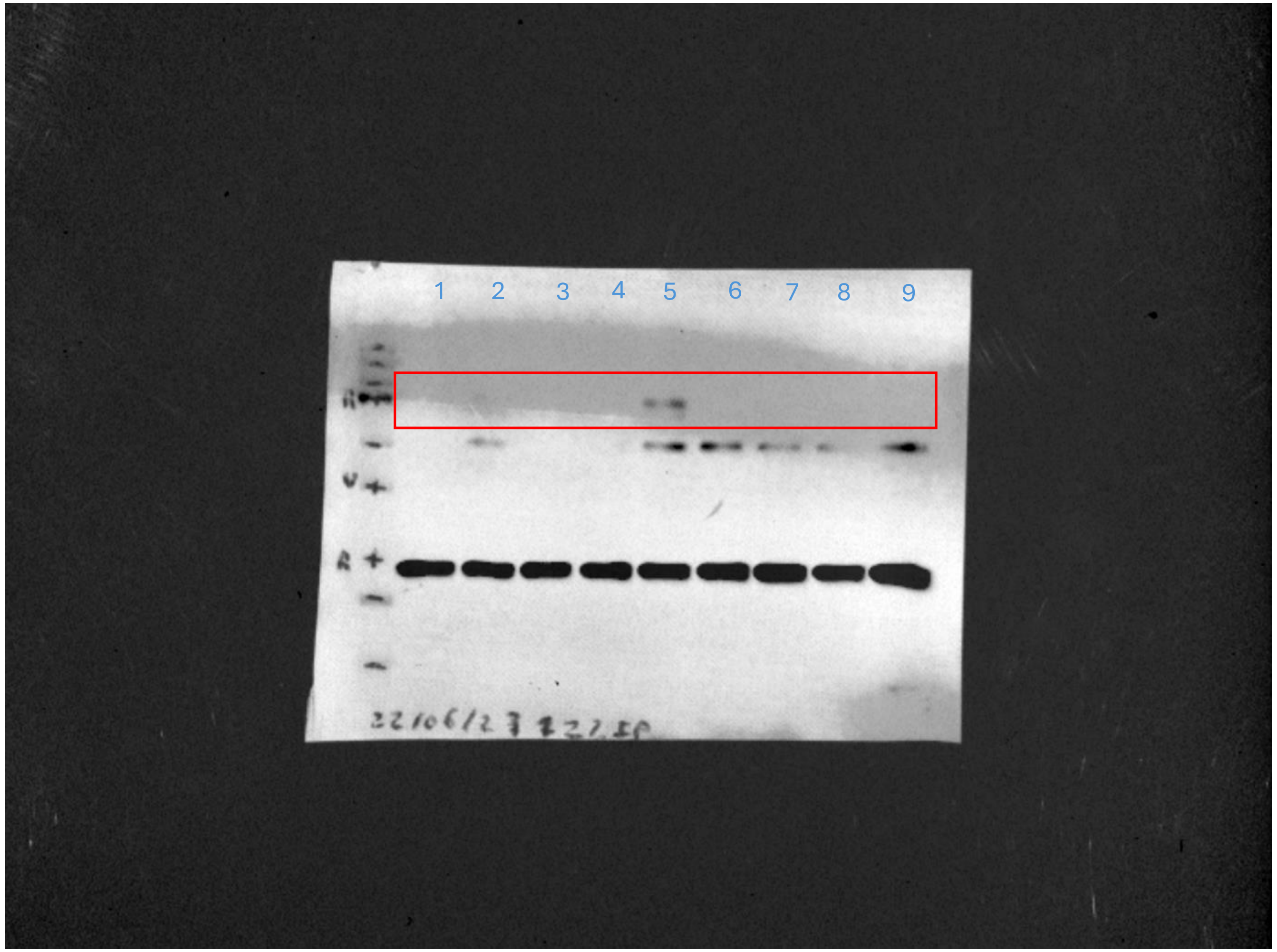

Anti-IGF2BP1

IP anti-HA

1- Mock IGF2BP2-HA (-) VCP-HA(-)
2- Mock IGF2BP2-HA (+) VCP-HA(-)
3- Mock IGF2BP2-HA (-) VCP-HA(+)
4- ZIKV IGF2BP2-HA (-) VCP-HA(-)
5- ZIKV IGF2BP2-HA (+) VCP-HA(-)
6- ZIKV IGF2BP2-HA (-) VCP-HA (+)
7- DENV IGF2BP2-HA (-) VCP-HA(-)
8- DENV IGF2BP2-HA (+) VCP-HA (-)
9- DENV IGF2BP2-HA (-) VCP-HA(+)

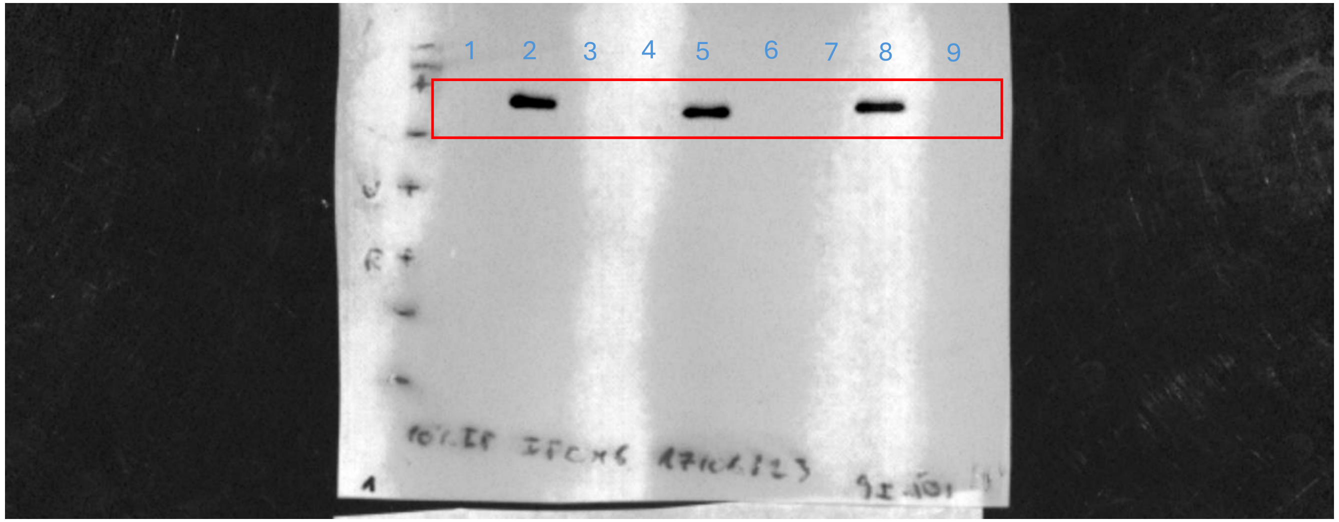

Anti-IGF2BP3

IP anti-HA

1- Mock IGF2BP2-HA (-) VCP-HA(-)
2- Mock IGF2BP2-HA (+) VCP-HA(-)
3- Mock IGF2BP2-HA (-) VCP-HA(+)
4- ZIKV IGF2BP2-HA (-) VCP-HA(-)
5- ZIKV IGF2BP2-HA (+) VCP-HA(-)
6- ZIKV IGF2BP2-HA (-) VCP-HA (+)
7- DENV IGF2BP2-HA (-) VCP-HA(-)
8- DENV IGF2BP2-HA (+) VCP-HA (-)
9- DENV IGF2BP2-HA (-) VCP-HA(+)

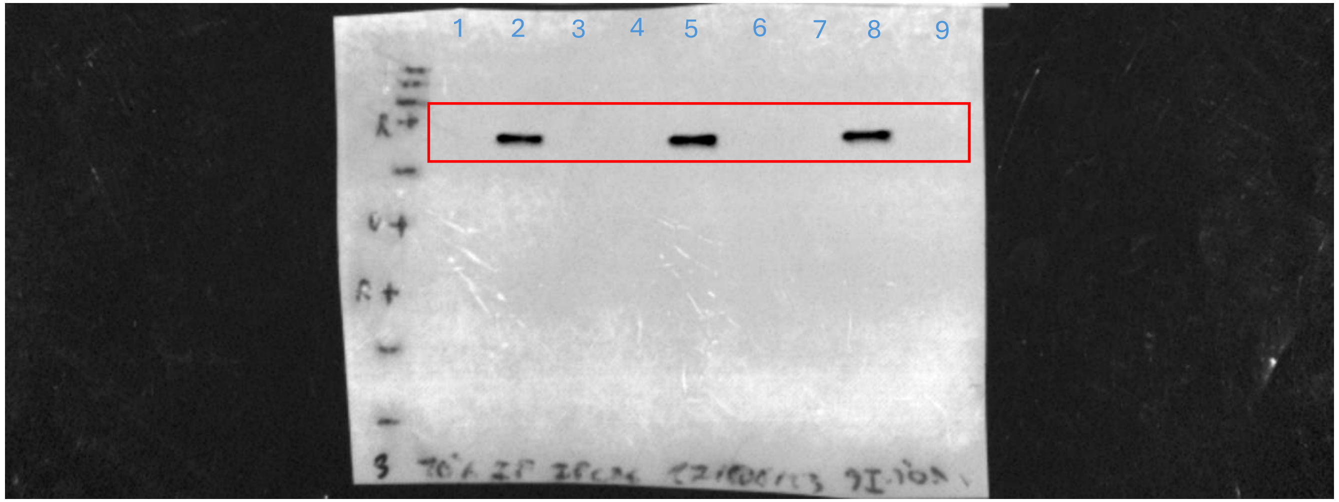

Anti-YBX1

IP anti-HA

1- Mock IGF2BP2-HA (-) VCP-HA(-)
2- Mock IGF2BP2-HA (+) VCP-HA(-)
3- Mock IGF2BP2-HA (-) VCP-HA(+)
4- ZIKV IGF2BP2-HA (-) VCP-HA(-)
5- ZIKV IGF2BP2-HA (+) VCP-HA(-)
6- ZIKV IGF2BP2-HA (-) VCP-HA (+)
7- DENV IGF2BP2-HA (-) VCP-HA(-)
8- DENV IGF2BP2-HA (+) VCP-HA (-)
9- DENV IGF2BP2-HA (-) VCP-HA(+)

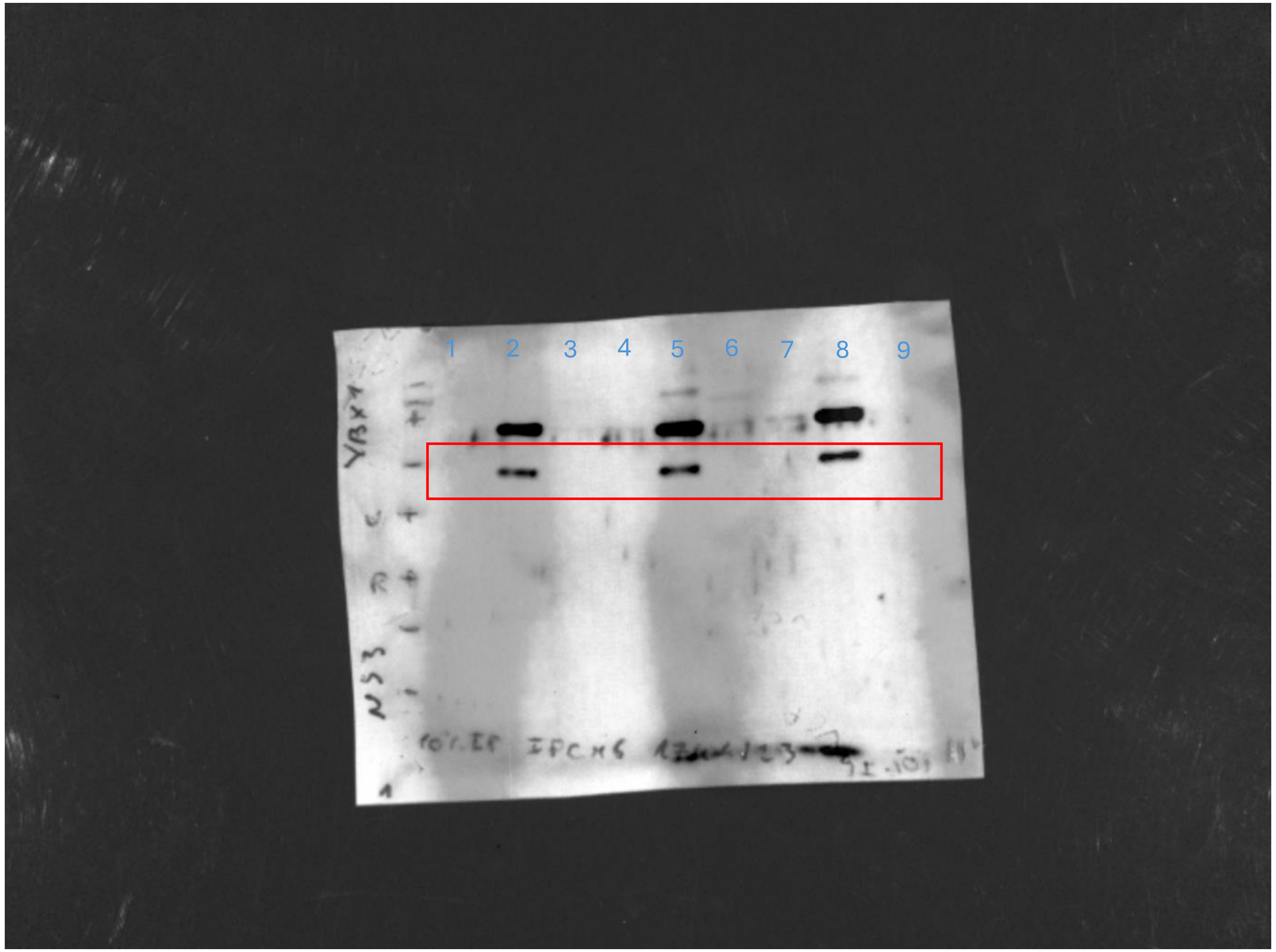

Supplement: Figure 9—figure supplement 2—source data 2. [file elife-94347-fig9-figsupp2-data2.zip › Figure 9-figure supplement 2-source data 2.pdf]
